# Gefitinib metabolism-related lncRNAs for the prediction of prognosis, tumor microenvironment and drug sensitivity in lung adenocarcinoma

**DOI:** 10.1038/s41598-024-61175-3

**Published:** 2024-05-06

**Authors:** Zishun Guo, Xin Zhang, Dingtao Yang, Zhuozheng Hu, Jiajun Wu, Weijun Zhou, Shuoming Wu, Wenxiong Zhang

**Affiliations:** 1 Department of Thoracic Surgery, The Second Affiliated Hospital, Jiangxi Medical College , Nanchang University, 1 Minde Road, Nanchang, 330006 China; 2 Department of Thoracic Surgery, The First People’s Hospital of Lianyungang, No. 6, Zhenhua East Road, Lianyungang, 222000 China

**Keywords:** Gefitinib, LncRNA, Lung adenocarcinoma, Drug sensitivity, Immunotherapy, Cancer, Cancer

## Abstract

The complete compound of gefitinib is effective in the treatment of lung adenocarcinoma. However, the effect on lung adenocarcinoma (LUAD) during its catabolism has not yet been elucidated. We carried out this study to examine the predictive value of gefitinib metabolism-related long noncoding RNAs (GMLncs) in LUAD patients. To filter GMLncs and create a prognostic model, we employed Pearson correlation, Lasso, univariate Cox, and multivariate Cox analysis. We combined risk scores and clinical features to create nomograms for better application in clinical settings. According to the constructed prognostic model, we performed GO/KEGG and GSEA enrichment analysis, tumor immune microenvironment analysis, immune evasion and immunotherapy analysis, somatic cell mutation analysis, drug sensitivity analysis, IMvigor210 immunotherapy validation, stem cell index analysis and real-time quantitative PCR (RT-qPCR) analysis. We built a predictive model with 9 GMLncs, which showed good predictive performance in validation and training sets. The calibration curve demonstrated excellent agreement between the expected and observed survival rates, for which the predictive performance was better than that of the nomogram without a risk score. The metabolism of gefitinib is related to the cytochrome P450 pathway and lipid metabolism pathway, and may be one of the causes of gefitinib resistance, according to analyses from the Gene Set Enrichment Analysis (GSEA), Gene Ontology (GO) and Kyoto Encyclopedia of Genes and Genomes (KEGG). Immunological evasion and immunotherapy analysis revealed that the likelihood of immune evasion increased with risk score. Tumor microenvironment analysis found most immune cells at higher concentrations in the low-risk group. Drug sensitivity analysis found 23 sensitive drugs. Twenty-one of these drugs exhibited heightened sensitivity in the high-risk group. RT-qPCR analysis validated the characteristics of 9 GMlncs. The predictive model and nomogram that we constructed have good application value in evaluating the prognosis of patients and guiding clinical treatment.

## Introduction

A malignant tumor that develops in the bronchi and alveoli is called lung cancer and is one of the most prevalent malignancies worldwide. Approximately 1.77 million people die of lung cancer every year. Approximately 50% of all lung tumors are LUADs, a highly heterogeneous subtype of lung cancer. LUAD often presents early in nonsmoking East Asian women, and EGFR mutations in LUAD are generally higher in Asian women^1^. As an inhibitor of EGFR-TK (epidermal growth factor receptor tyrosine kinase), patients with advanced LUAD and an EGFR mutation can benefit from gefitinib^2^. It has demonstrated therapeutic activity against lung cancer as a complete compound and has been shown to be metabolized by cytochrome P450 in the liver, including CYP3A5, CYP3A4 and CYP2D6^3^. However, the impact of gefitinib metabolism on lung cancer is unclear.

Currently, lung cancer patients mainly rely on imaging studies and tissue biopsies to evaluate lesions during treatment. However, imaging cannot evaluate the long-term prognosis of patients, and biopsy will bring pain to patients and even cause distant metastasis of tumors^4^. Therefore, researchers have investigated and discovered that molecular biomarkers are essential for predicting the prognosis of cancer. Related studies have proven that lncRNAs play a significant role in predicting cancer prognosis and guiding cancer treatment^5^. As found in the study of Taoli Wang et al., related lncRNAs may have an impact on gefitinib resistance^6^. Therefore, it is necessary to incorporate lncRNAs into clinical models to explore new prognosis-related biomarkers. In contrast, gefitinib metabolism-related lncRNAs have not been described.

Using gefitinib metabolism-related lncRNAs, we constructed a prognostic model and explored its predictive value for LUAD patient prognosis.

## Materials and methods

### Related genes and patient data downloads

**Figure 1:**
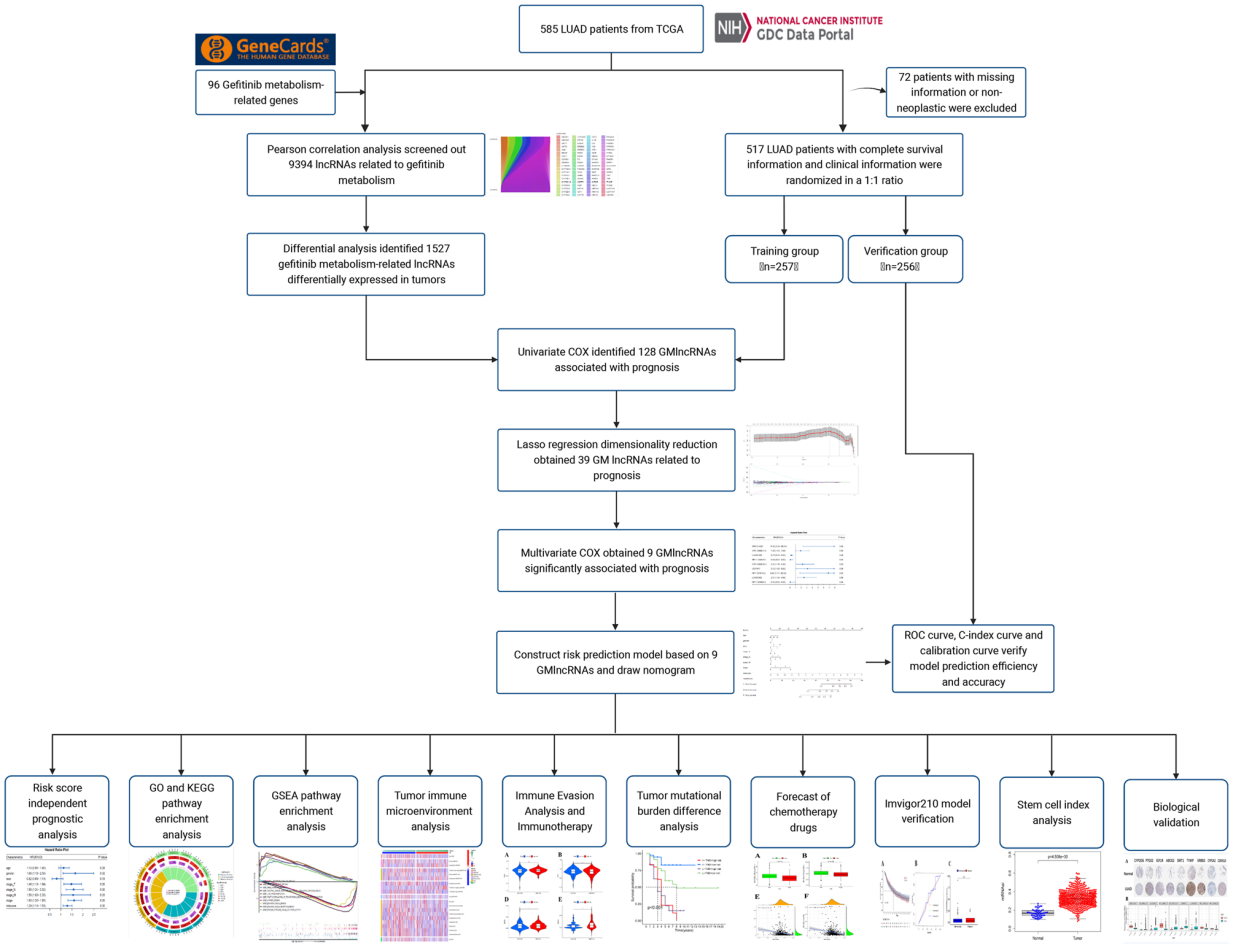
Flowchart.

We obtained 96 genes connected to gefitinib metabolism for this research by using the GeneCards website (https://www.genecards.org/)^7^. Subsequently, we obtained gene expression data and clinicopathological as well as prognostic information of 585 patients diagnosed with lung adenocarcinoma (LUAD) from The Cancer Genome Atlas (TCGA) database, which is publicly accessible at https://portal.gdc.cancer.gov/. Following the exclusion of 72 patients with incomplete data on survival, the analysis comprised a total of 513 patients. Data from the IMVigor210 clinical trial were obtained through the European Genome-Phenome Archive (https://ega-archive.org/) (Table 1). Figure 1 shows our research workflow.

**Table 1:** Clinical information of 513 LUAD samples in the TCGA database.

Features	Train cohort		Test cohort		Entire cohort	
	(n = 257)		(n = 256)		(n = 513)	
	n	%	n	%	n	%
Status						
Alive	166	64.6	162	63.3	328	63.9
Dead	91	35.4	94	36.7	185	36.1
Age						
< 50	18	7.0	17	6.6	35	6.80
50–59	51	19.8	54	21.1	105	20.5
60–69	93	36.2	82	32.0	175	34.1
70–79	73	28.4	84	32.8	157	30.6
≥ 80	16	6.2	15	5.9	31	6
Unknown	6	2.3	4	1.6	10	1.9
Gender						
Female	136	52.9	140	54.7	276	53.8
Male	121	47.1	116	45.3	237	46.2
Race						
White	196	76.3	201	78.5	397	77.4
Asian	2	0.8	6	2.3	8	1.6
Black	33	12.8	21	8.2	54	10.5
Unknown	26	10.1	28	10.9	54	10.5
Stage						
Stage I	136	52.9	144	56.3	280	54.6
Stage II	60	23.3	60	23.4	120	23.4
Stage III	45	17.5	35	13.7	80	15.6
Stage IV	9	3.5	16	6.3	25	4.9
Unknown	7	2.7	1	0.4	8	1.6
T stage						
T1	89	34.6	82	32.0	171	33.3
T2	134	52.1	141	55.1	275	53.6
T3	23	8.9	23	9.0	46	9
T4	10	3.9	8	3.1	18	3.5
Unknown	1	0.4	2	0.8	3	0.6
M stage						
M0	172	66.9	172	67.2	344	67.1
M1	10	3.9	15	5.9	25	4.9
Unknown	76	29.6	68	26.6	144	28.1
N stage						
N0	160	62.3	175	68.4	335	65.3
N1	52	20.2	42	16.4	94	18.3
N2	40	15.6	30	11.7	70	13.6
N3	1	0.4	1	0.4	2	0.4
Unknown	6	2.3	6	2.3	12	2.3

*LUAD* lung adenocarcinoma, *TCGA* The Cancer Genome Atlas, *T* tumor, *N* node, *M* metastasis.

### Defining gefitinib metabolism-related lncRNAs

**Figure 2:**
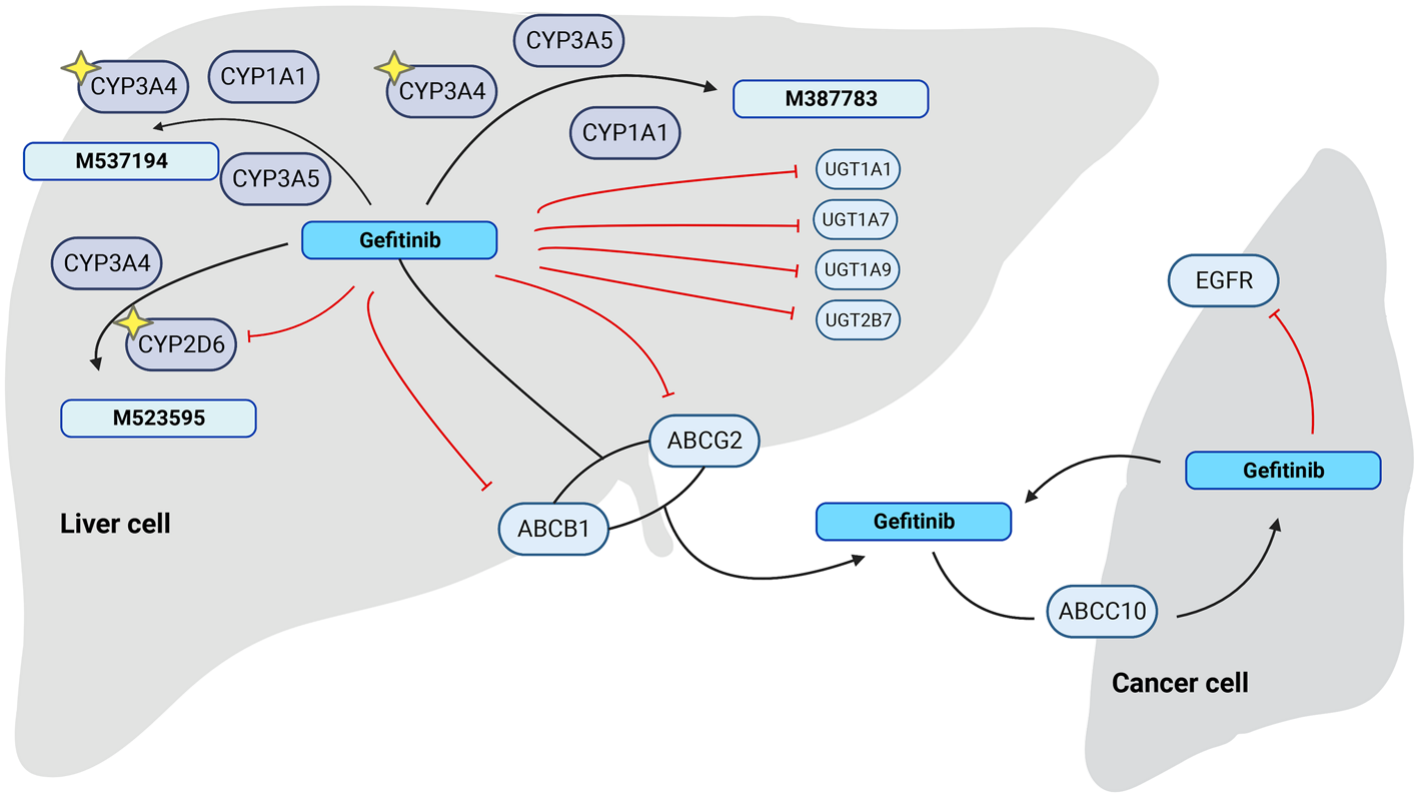
Metabolism of gefitinib.

Based on previous studies, 96 gefitinib metabolism-related genes were obtained. The metabolic mechanism of gefitinib is shown in Fig. 2. We examined the Pearson association between gefitinib metabolism-related genes and lung adenocarcinoma lncRNAs, and GMLncs were determined with |R| > 0.3 and P < 0.001 as the standard.

### Build a predictive model

We randomly divided 513 LUAD patients into a validation group (n = 256) and a training group (n = 257) in a 1:1 ratio. The training group was utilized to create the model, and univariate Cox analysis was applied to identify lncRNAs that exhibited a significant association with prognosis for patients (P < 0.05). Considering the influence of multicollinearity among variables, multivariate Cox regression and LASSO were employed to reduce dimensionality. Finally, 9 GMLncs (GMLncs) (WWC2-AS2, CTD-2066L21.3, LINC00355, RP11-246K15.1, CTD-2555C10.3, OGFRP1, RP11-879F14, LINC00862, RP11.345M22.2) were screened out. The formula for constructing the prediction model is “Riskscore = ∑exp(lncRNAs) p * β”, and “β” is the coefficient of multivariate Cox analysis for each lncRNA. We divided the cohort of samples into low-risk or high-risk subgroups by median risk score.

### Validation model and nomogram

The correctness of the model was assessed by both the verification group and the entire group. OS in the low- and high-risk subgroups was examined by survival curves based on Kaplan‒Meier analysis. The connection of other pathological variables with prognosis was assessed using Cox analysis, both multivariate and univariate. (e.g., age, sex, race, AJCC stage). We employed concordance index curves (C-index) and receiver operating characteristic curves (ROC) to measure the risk score's ability to predict the eventual results^8^. The model was then visualized using a nomogram. To compare the differences in linked genes between subgroups at low and high risk, principal component analysis (PCA) was utilized.

### Analysis of GO/KEGG pathways

To investigate further potential distinctions in biology among the subgroups, we compared the expression levels of all lncRNAs in the patient subgroups of low risk and high risk and identified retrieved lncRNAs with significant differences (P < 0.05). Then, a study of all the various lncRNAs was performed using GO and KEGG analysis^9^. The “Limma” and “ClusterProfiler” R packages were used for pathway analysis (P < 0.05 and FDR < 0.05).

### GSEA enrichment analysis

Using P < 0.05 as the standard, we conducted GSEA on 6 databases^10^, including GO, KEGG, REACTOME, BIOCRATA, PID and WIKIPATHWAYS. The GSEA algorithm is an efficient tool for genomic research and can evaluate microarray data at the gene set level. The analysis was completed with GSEA4.3.2.

### Tumor microenvironment analysis

A vital factor influencing the development of cancer is the tumor microenvironment (TME). Based on single-sample enrichment analysis (ssGSEA)^11^, we used seven different algorithms to analyze immunological cells associated with the low- and high-risk subgroups. Next, according to the CIBERSORT method^12^, we identified 22 types of immunological cells, which varied among both groups. Finally, we analyzed 13 functional pathways involved in immunity in the test, training and entire groups.

### Immune evasion analysis and immunotherapy

A study of related lncRNAs and tumor immune evasion was conducted. A computational method called tumor immune dysfunction and exclusion (TIDE) imitates two key aspects of tumor immunity. Inhibiting T-cell infiltration in cancers which are heavily infiltrated with cytotoxic T lymphocytes (CTLs) and preventing T-cell infiltration in cancers with low levels of CTLs. TIDE provides the data^13^. Additionally, we evaluated CD8, cluster of differentiation 274 (CD274), Merck18, cancer-associated fibroblasts (CAFs), tumor-associated macrophage M2 (TAMM2), myeloid-derived suppressor cells (MDSCs), IFGN and microsatellite instability (MSI) score as additional immunological biomarkers^14–16^.

### Tumor mutational burden analysis

The frequency of gene insertion or deletion errors per million bases identified in a cancer, the number of base substitutions and somatic gene coding errors is known as the tumor mutational burden (TMB). A higher TMB value predicted better immunotherapy outcomes^17^. The “limma” package was utilized to conduct a comparative analysis of TMB between subgroups categorized as low-risk and high-risk, followed by visualization of the results. Mutation data were acquired from TCGA.

### Chemotherapy drug discovery and screening

We used the “OncoPredict” software package and applied The Genomics of Drug Sensitivity in Cancer (GDSC) database for medicine susceptibility analysis to identify potential medicines for LUAD patient treatment^18^.

### Correlation with response to immunotherapy in IMvigor210

IMvigor210 was used to validate the lncRNA’s prognostic value^19^. Regarding the efficacy and safety of atezolizumab, the IMvigor210 study evaluated the efficacy of an antibody that targets programmed cell death of ligand 1 (PD-L1) in patients with advanced local or metastasized urothelial disease who had previously undergone platinum therapy. Data were downloaded by using the IMvigor210CoreBiologies package. After performing LASSO regression analysis, we matched the obtained genes with those present in the above model. Using the identical formula, the risk score was computed for patients belonging to the IMvigor210 cohort. Patients were then separated into low- and high-risk groups.

### Stem cell index clinical correlation (mRNAsi)

The stem cell index is an evaluative metric that delineates the extent of similarity between neoplastic cells and stem cells. The stem cell index can be used as a prognostic tool to evaluate the probability of tumor repetition^20^. mRNAsi is an indicator calculated from the expression data of a gene. We studied the correlation between the stemness index and LUAD patient survival rate. The Wilcoxon test was employed to examine the potential association between the stemness index and the sex, T stage, M stage, and AJCC stage of patients diagnosed with LUAD.

### Biological verification

Human LUAD cell lines (A549, H1650, H1299, H1975, and PC9) and normal lung cells (BEAS-2B) were purchased from Fuheng Biotechnology (Shanghai, China) and underwent authentication via short tandem repeat analysis. Cells were cultured in 1% penicillin/streptomycin (HyClone) and 10% fetal bovine serum (FBS; Sage Creation Science Co. Ltd., Beijing, China) Dulbecco's Modified Eagle Medium (DMEM; HyClone, Logan, UT, USA) for monolayer.

The TRIzol™Plus RNA Purification Kit (Invitrogen, Thermo Fisher Scientific, Inc.) was utilized to extract total RNA from cells according to the manufacturer's instructions. The RNA was then reverse transcribed into cDNA according to PrimeScript RT Master Mix's guidelines (Takala, Japan). The cDNA concentration and purity were assessed. Next, the SYBR Premix Ex Taq II kit (Takala, Japan) was utilized to perform qRT-PCR. The polymerase chain reaction (PCR) was executed using a procedure that involved an initial denaturation step of 30 s at 95 °C, followed by 40 cycles of decomposition at 95 °C for 5 s, annealing for 30 s at 60 °C, extension for 45 s at 72 °C, and a final extension step for 10 min at 72 °C. The study employed the internal reference gene β-actin and the resulting data were analyzed using the prescribed methodology as outlined in Ref.^21^. Table S1 displays the primer sequences.

We analyzed the protein expression changes of the 10 highest gefitinib metabolism-related genes in LUAD and normal cells by employing the Human Protein Atlas (HPA) website.

### Analytical statistics

R program version 4.2.1 was applied to carry out the statistical evaluation. P values are two-way, and “< 0.05” is considered statistically significant.

### Ethics approval and consent to participate

This article does not contain any studies with human participants or animals performed by any of the authors. The data of this paper is extracted from TCGA database, where it is publicly available and unrestricted re-use is permitted via an open license. This study is exempt from ethics committee approval by default.

## Result

### Gefitinib metabolism-related lncRNA identification

**Figure 3:**
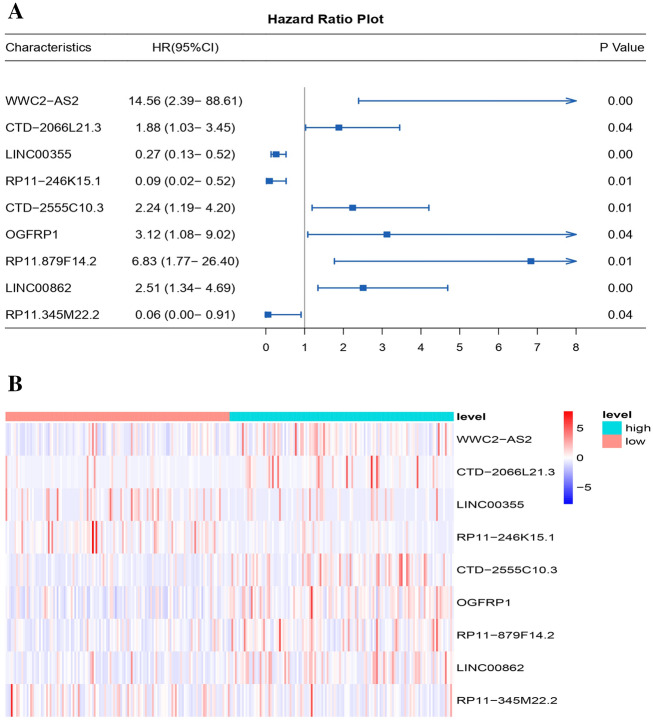
9 GMLncs for building models. (**A**) Forest plot of 9 lncRNAs related to gefitinib metabolism obtained by multivariate COX; (**B**) Heatmap of 9 lncRNAs related to gefitinib metabolism obtained by multivariate COX.

First, the R package “limma” was used. Using P < 0.001 and |R| > 0.3 as criteria, 9396 GMLncs were identified. We illustrate this step in a Sankey diagram in Figure S1A. Next, univariate Cox analysis was performed, and 128 lncRNAs were determined to be strongly correlated with the survival of patients in relation to gefitinib metabolism (Table S2). Based on the abovementioned lncRNAs related to prognosis, LASSO regression was employed to decrease the dimension in the training group. After selecting a suitable λ value, 39 lncRNAs were involved (Fig. S1B,C). Multivariate COX analysis results were further studied, and 9 lncRNAs were determined to be significantly related to gefitinib metabolism (including WWC2-AS2, CTD-2066L21.3, LINC00355, RP11-246K15.1, CTD-2555C10.3, OGFRP1, RP11-879F14.2, LINC00862, and RP11-345M22.2) (Table S3). We visualized these lncRNAs using forest plots and heatmaps (Fig. 3A,B). We summarize the phenotypes and potential target proteins related to 9 GMlncs in Table S4 for subsequent research.

### Prognostic model construction and validation

**Figure 4:**
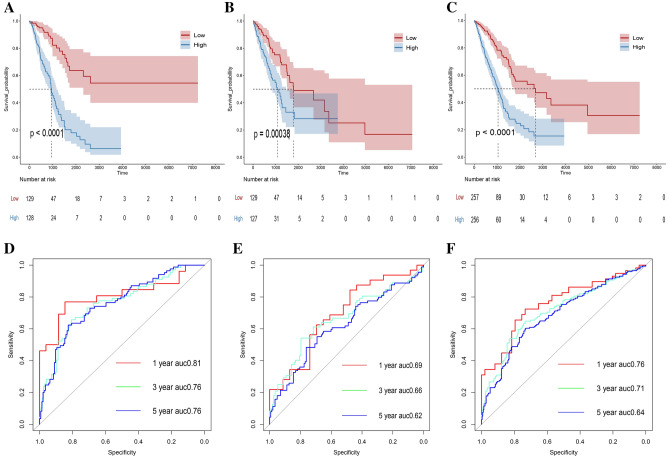
The prediction effect and verification of the model. (**A**) KM curve of training group; (**B**) KM curve of validation group; (**C**) KM curve of entire group; (**D**) 1, 3 and 5-year ROC curves of training group; (**E**) 1, 3 and 5-year ROC curves of validation group; (**F**) 1, 3 and 5-year ROC curves of entire group.

The training group was segregated into two subgroups, namely, the low-risk subgroup (n = 129) and the high-risk subgroup (n = 128), based on the median risk score. Figure S2 illustrates the risk score grouping effect in the training group, validation group and entire group. Figure 4A displays the Kaplan‒Meier survival curves for the training group, showing that patients classified in the high-risk subgroup exhibited a comparatively inferior prognosis in contrast to those in the low-risk subgroup. The curves of the ROC indicated that the model exhibited considerable prediction effectiveness over the course of 1-year, 3-year, and 5-year periods (0.81, 0.76, and 0.76), as illustrated in Fig. 4D. We also observed the same trend in the validation group and entire group (Fig. 4B,C, E,F).

### Nomograms and clinical relevance

**Figure 5:**
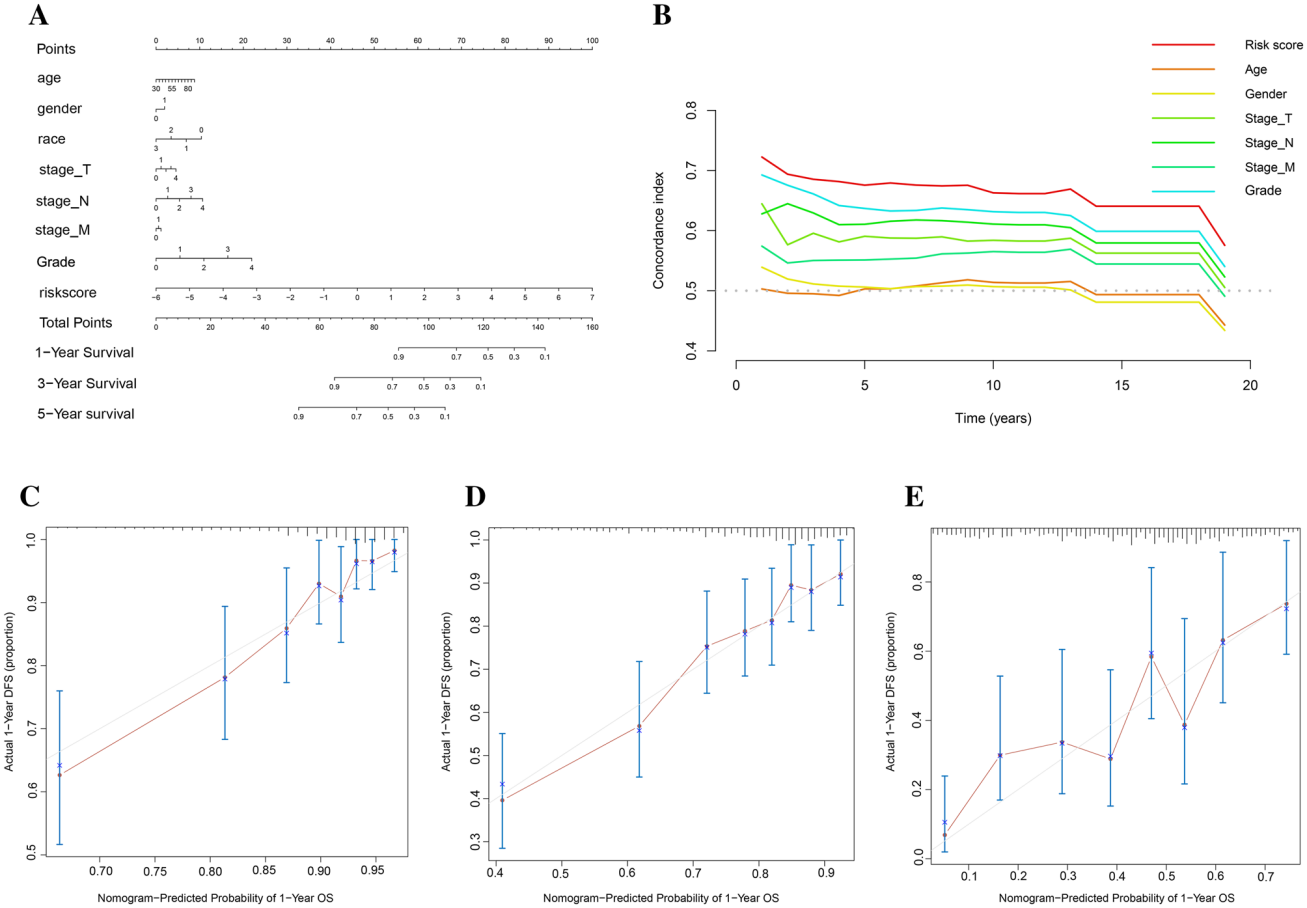
Prognostic Model Construction and Validation. (**A**) A nomogram constructed based on nine gefitinib metabolism-related lncRNAs and clinically relevant features; (**B**) C-index curve of risk model; (**C**) 1-year Calibration curve of the nomogram; (**D**) 3-year Calibration curves of the nomogram; (**E**) 5-year Calibration curve of the nomogram.

Subsequently, the nomogram was developed by integrating the risk score with relevant clinical features (Fig. 5A). The C-index curve indicated that the risk score has a greater capacity for prediction than other clinical parameters (Fig. 5B). The calibration curves exhibited satisfactory alignment among the anticipated outcomes of survival from the nomogram and the actual observed survival at 1 year, 3 years, and 5 years (Fig. 5C–E). The predictive power of the predictive model including the risk score was also higher than that of the predictive model with only clinical parameters (Fig. S3). Clinical correlation analysis showed that risk scores were not correlated with age and sex, but high-risk scores corresponded to poorer clinical features, including AJCC stage, T stage, and N stage (Fig. S4A–E).

### Clinical independent prognostic model analysis

**Figure 6:**
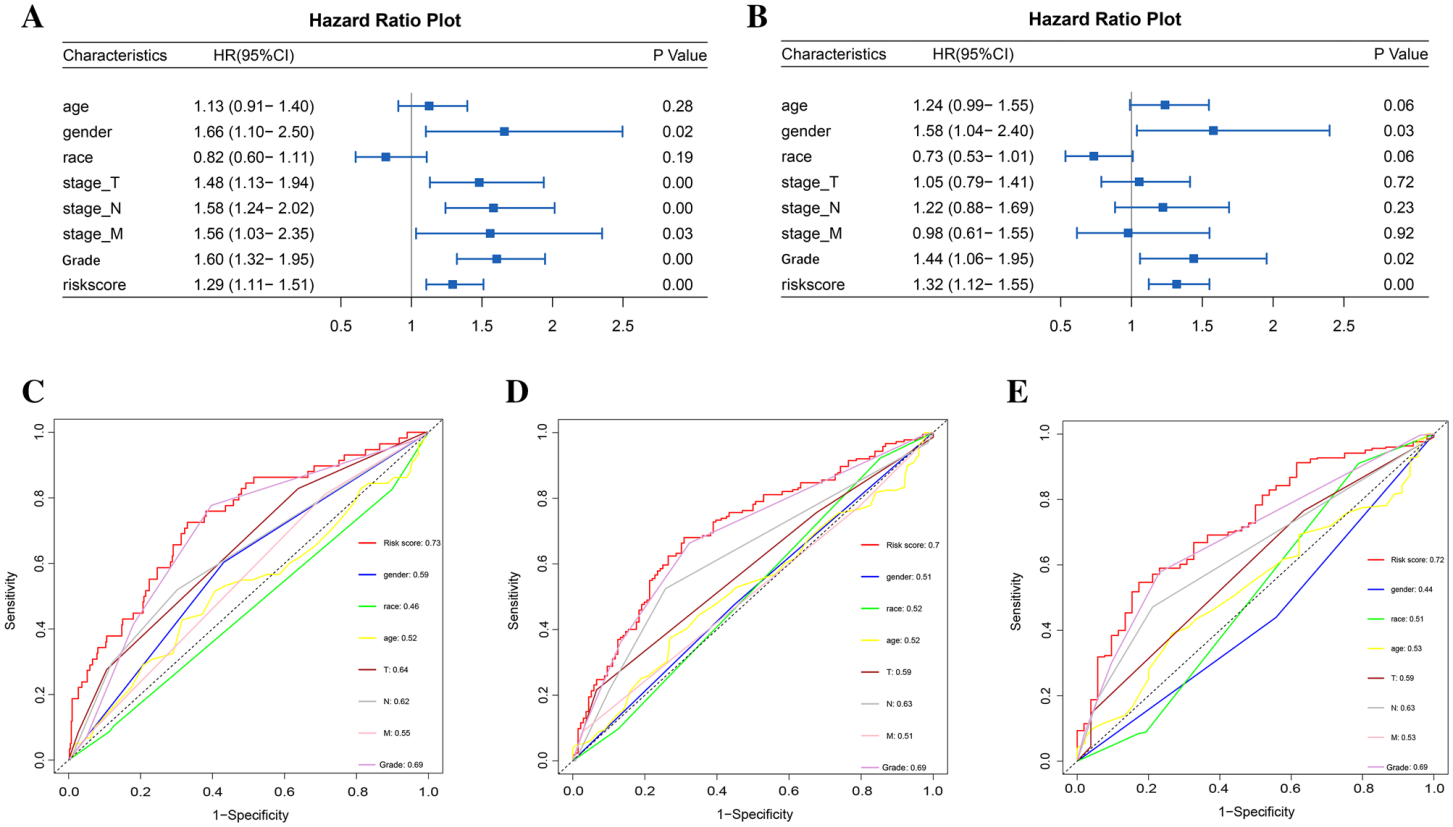
Independent prognostic analysis of risk score. (**A**) Univariate COX analysis of clinicopathological features; (**B**) Multivariate COX analysis of clinicopathological features; (**C**) ROC curve for risk score and clinicopathological factors at 1-year; (**D**) ROC curve for risk score and clinicopathological factors at 3-year; (**E**) ROC curve for risk score and clinicopathological factor at 5-year.

Univariate and multivariate Cox analyses were utilized to evaluate the relationship between clinical and pathological features (gender, age, race, AJCC stage, T stage, N stage, and M stage), risk score, and samples’ survival condition. The outcomes of the univariate Cox analysis revealed significant statistical associations between overall survival (OS) and various factors, such as risk score, sex, T stage, N stage and AJCC stage (Table S5). The diagram is depicted in Fig. 6A. Multivariate Cox analysis demonstrated significant associations between OS and risk score, sex and AJCC stage (Fig. 6B). Risk score is an independent prognostic factor for patient outcome. The quality of the prognostic model that is independent of patient characteristics was evaluated through the utilization of ROC curves. The results demonstrated that AJCC stage (0.77, 0.72, 0.68), T stage (0.71, 0.64, 0.61), N stage (0.70, 0.67, 0.65) and risk score (0.81, 0.76, 0.76) had substantial value in determining the 1-, 3-, and 5-year survival rates of LUAD patients (Fig. 6C–E). Overall, the risk score demonstrated a higher predictive value than other factors, indicating its stronger association with patient outcomes. Other factors, such as AJCC, T, and N stage, can still serve as useful reference indicators in assessing prognosis. Using the KM curve, the prognostic prediction ability of some clinical subgroups has been verified, but the prediction effect of some clinical subgroups is unsatisfactory (Fig. S5). PCA analysis showed that the modeled lncRNAs were able to effectively divide patients into two subgroups (Fig. S6).

### GO/KEGG pathway enrichment analysis

**Figure 7:**
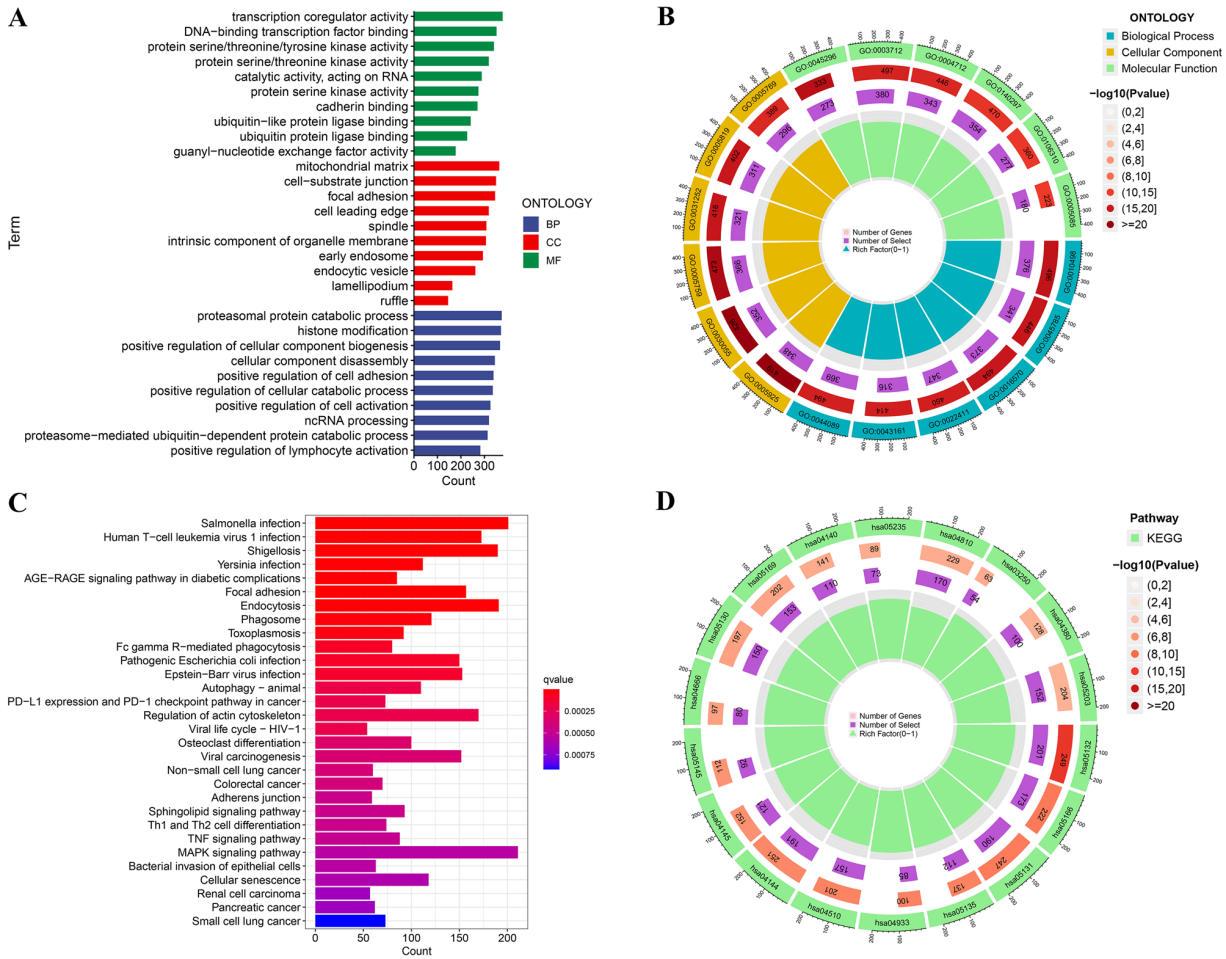
Pathway enrichment analysis. (**A**) Bar plot of top ten pathways in GO pathway enrichment analysis; (**B**) Pie chart of the top ten pathways in GO pathway enrichment analysis; (**C**) Bar plot of top thirty pathways in KEGG pathway enrichment analysis; (**D**) Pie chart of top thirty pathways in KEGG pathway enrichment analysis.

We conducted a differential analysis of lncRNA expression levels among the patient subgroups at low and high risk and extracted lncRNAs with significant differences (FDR < 0.05) for pathway analysis. We visualized the top 10 significantly correlated pathways from the GO enrichment analysis and the top 30 significantly correlated pathways from the KEGG enrichment analysis (Fig. 7A–D). We found that in the GO enrichment analysis, there were many pathways closely related to lung cancer, such as GO:0044782: cilium organization and GO:0060271: cilium assembly. At the same time, there were also differences in pathways related to lipid metabolism, such as GO:0046486: Glycerolipid metabolic process, GO:0006650: Glycerophospholipid metabolic process, and GO:0,009,062: fatty acid catalytic process. It should be noted that GO:0044282: small molecule catalytic process and GO:0004712: protein serine/threonine/tyrosine kinase activity may be closely connected to the cancer suppressor mechanism and metabolic process of gefitinib. We also noticed variations in lipid metabolism-related pathways in the KEGG enrichment study, including hsa00071: Fatty acid degradation and hsa00564: Glycerophospholipid metabolism. In addition, pathways related to drug resistance were observed in both GO and KEGG enriched pathways, such as GO:0051092: positive regulation of NF-kappaB transcription factor activity and hsa01524: Platinum drug resistance, etc. In addition, the differential expression of hsa05171: Coronavirus disease-COVID-19 may suggest that COVID-19 is a new risk factor for LUAD. The results of the enrichment analysis revealed the potential mechanism of gefitinib's impact on cancer during metabolism. The pathways obtained by GO and KEGG enrichment analysis are listed in Tables S6 and S7.

### GSEA enrichment analysis

We present some of the characteristic pathways in Fig. S7. In GO and WP, gefitinib metabolism-related pathways (GOBP Epoxygenase P450 Pathway and Oxidation by Cytochrome P450) were strongly elevated in the high-risk subgroup. These pathways verified the role of cytochrome P450 in the process of gefitinib metabolism. This suggests that patients with high-risk lung cancer may have enhanced metabolism of gefitinib. Multiple pathways related to NF-κB activity were also found in the high-risk group, which is considered to be one of the mechanisms leading to cancer development and EGFR inhibitor resistance. The relationship between gefitinib metabolism and lipid metabolism was also confirmed in GO (GOBP Lipid Phosphorylation). In the GSEA of 6 databases, a variety of pathways that promote the progression of LUAD were strongly expressed in the high-risk subgroup, including GOBP: ERBB Signaling Pathway, Biocarta TGFβ Pathway, Biocarta P38MAPK Pathway, and WP Neovascularization Processes. These pathways are suggested to be related to the high risk of LUAD, which offers a direction for additional investigation. Regrettably, we did not obtain significantly enriched pathways among the KEGG pathways. The pathways obtained by GSEA enrichment analysis are listed in Tables S8 and S9.

### Tumor immune microenvironment analysis

Many immune cells were found in larger densities in the low-risk group, as shown by 7 immunological algorithms (Fig. S8A). Next, the CIBERSOFT algorithm was employed to research the immune microenvironment in the low- and high-risk groups, and the outcomes indicated that monocytes, quiescent dendritic cells, and neutrophils were abundant in the low-risk subgroup. M0 macrophages were abundant in the high-risk subgroup (Fig. S8B). The heatmap displays 13 immune function pathways, showing the differential expression between the two subgroups. The outcomes showed that in the training group, there were no differentially expressed immune function pathways (Fig. S8C). The low-risk subgroup in the verification group exhibited high expression levels of HLA, T-cell costimulation, T-cell coinhibition, and the Checkpoint Marker, while the high-risk subgroup displayed low expression levels of these markers (Fig. S8D) In the entire group, MHC class I was poorly expressed in the low-risk subgroup and strongly expressed in the high-risk subgroup across the board, demonstrating that it is a high-risk route (Fig. S8E).

### Immune evasion analysis and immunotherapy

The present study investigated the impact of immunotherapy and cancer immune evasion on related lncRNAs in both low-risk and high-risk subgroups. However, our findings indicate that the TIDE score did not demonstrate statistical significance in the training, test, or entire groups. The MDSC, CAF, and Exclusion variables exhibited significant differences between the low-risk and high-risk subgroups, with higher scores observed in the latter (Fig. S9). The risk score and IFNG expression were inversely correlated. Other immunological markers, including TAMM2, MSI, Merck18, Dysfunction, CD8, and CD274, are shown in Fig. S10.

### Tumor mutational burden difference analysis

A comparison was made among the TMB of subgroups categorized as low-risk and high-risk. In the training group (P = 0.92), test group (P = 0.61), and entire group (P = 0.94), the results demonstrated that no differences were found in the TMB of the low- and high-risk subgroups (Fig. S11A–C). The training group's and the test group's TMB survival curve results (P = 0.054, P = 0.061) are meaningless. However, the overall group's result (P = 0.017) demonstrates that there is an obvious distinction between the survival curves of the H-TMB subgroup and the L-TMB subgroup, and the OS of the H-TMB subgroup is higher than that of the L-TMB subgroup (Fig. S11D–F). The relationship between TMB, low-risk group, high-risk group, and survival was then examined using a survival curve. The results indicate that the “L-TMB + high-risk” cohort exhibits the most abbreviated survival interval among the entire cohort, while the “H-TMB + low-risk” cohort displays the most prolonged survival duration. The test and training group results were consistent with those of the entire group (Fig. S11G–I).

### Forecast of chemotherapy drugs

The samples’ response to therapy was estimated using the “OncoPredict” algorithm, thereby identifying possible chemotherapy medications for our model. The semi-maximum inhibitory concentration (IC50) from the GDSC database was used by that algorithm. A total of 23 chemical drugs underwent screening, and a significant variation in the estimated semi-inhibitory concentration deposits was observed between the low-risk and high-risk subgroups. Twenty-one drugs had high sensitivity in the high-risk subgroup. The boxplots and their correlation plots for the top 4 sensitive drugs (Fig. S12). Further details are shown in Table 2.

**Table 2:** The 23 chemotherapeutic drugs obtained from drug sensitivity analysis.

Drug	P-value	H.median (25%, 75%)	L.median (25%, 75%)
AZD7762_1022	0.00	0.66 (0.38–1.46)	0.96 (0.50–2.29)
Pevonedistat_1529	0.00	1.36 (0.67–3.16)	2.03 (0.98–4.61)
BMS-754807_2171	0.00	2.18 (0.83–3.93)	1.47 (0.40–2.94)
Luminespib_1559	0.00	0.07 (0.04–0.16)	0.1 (0.05–0.25)
Staurosporine_1034	0.00	0.03 (0.02–0.07)	0.05 (0.02–0.10)
Camptothecin_1003	0.00	0.07 (0.04–0.14)	0.12 (0.05–0.23)
AZD6738_1917	0.00	5.26 (2.75–10.24)	7.62 (3.88–20.31)
Sapitinib_1549	0.00	34.65 (21.69–66.58)	50.78 (28.47–88.84)
Gemcitabine_1190	0.00	0.36 (0.13–0.97)	0.62 (0.23–1.16)
5-Fluorouracil_1073	0.00	72.31 (30.73–186.18)	110.95 (48.31–350.57)
Cytarabine_1006	0.00	4.23 (2.00–8.34)	6.70 (3.18–12.72)
Foretinib_2040	0.00	1.76 (1.03–2.94)	2.43 (1.37–4.70)
Entospletinib_1630	0.00	34.87 (25.40–52.00)	44.21 (30.72–64.04)
VX-11e_2096	0.00	13.56 (7.49–22.56)	17.38 (11.17–32.34)
Trametinib_1372	0.00	1.18 (0.47–3.36)	2.21 (0.99–5.34)
Dasatinib_1079	0.00	3.07 (0.65–10.11)	5.84 (1.91–21.79)
Doramapimod_1042	0.00	101.72 (79.06–124.58)	86.28 (69.21–106.11)
PD0325901_1060	0.00	1.25 (0.68–2.25)	1.78 (1.14–3.24)
ERK_2440_1713	0.00	10.30 (5.82–19.15)	15.14 (8.99–32.21)
Ulixertinib_1908	0.00	13.34 (8.14–19.01)	18.03 (11.91–28.02)
Selumetinib_1736	0.00	44.34 (26.53–88.61)	71.91 (42.00–141.25)
ERK_6604_1714	0.00	25.25 (13.87–44.16)	37.22 (23.14–63.31)
SCH772984_1564	0.00	10.05 (3.40–22.54)	19.86 (10.32–39.52)

*P-value* probability, *H* high, *L* low.

### Imvigor210 model verification

We screened the same genes between the Lasso analysis and the IMVIGOR210 model, namely, LINC00862, WWC2-AS2 and OGFRP1. To verify the predictive value of linked lncRNAs and separate patients into low-risk or high-risk subgroups, the relevant risk score of patients in the Imvigor210 queue was generated. The KM survival curve demonstrates that Imvigor210 bladder cancer target gene expression is not statistically significant in the survival probability of the two subgroups. The ROC curve also indicates that this model's prediction performance is low. The risk scores of the target genes of different drug reactions of Imvigor210 bladder cancer were not statistically significant (P = 0.9) (Fig. S13).

### LUAD patient stem cell index analysis

Stem cell index analysis revealed significant differences in the mRNAsi of non-cancerous and cancerous samples (P < 0.05). Survival analysis, however, revealed that mRNAsi and OS were not significantly associated with low or high expression (Fig. S14A,B). Then, we examined the correlation among the mRNAsi scores and clinical characteristics, including sex, AJCC staging, and T and M stages, and found that there were significant differences in their existence, indicating that the mRNAsi of LUAD patients was highly related to clinical features (Fig. S14C–F).

### Biological validation

**Figure 8:**
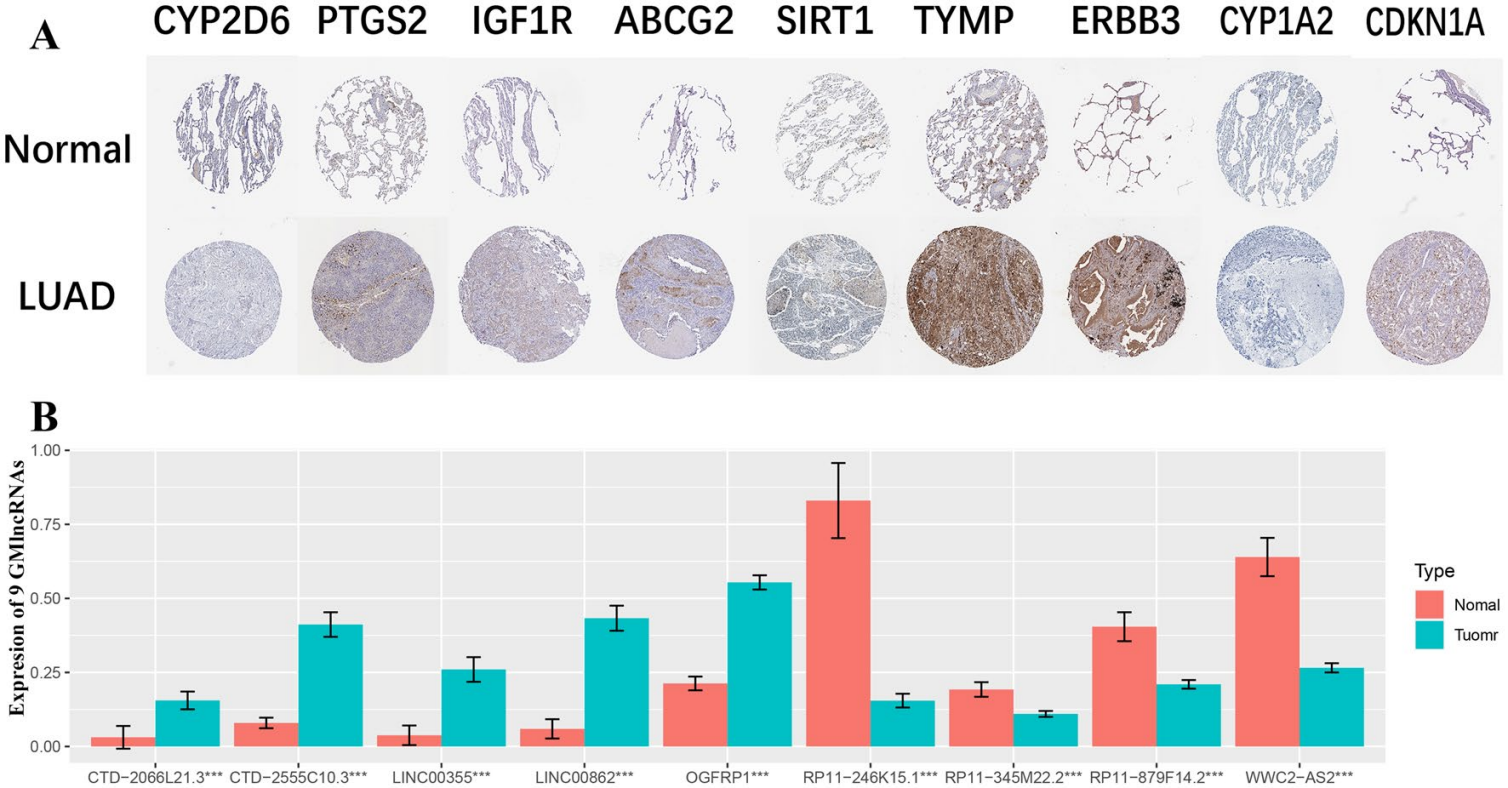
Experimental validation of gene expression. (**A**) Expression of genes related to gefitinib metabolism; (**B**) Differences in the expression of lncRNAs related to gefitinib metabolism in high and low risk groups.

The HPA database was utilized to compare the levels of protein expression of gefitinib metabolism-related genes in LUAD and normal cells. Figure 8A depicts the protein expression of 9 of the genes. MIR122 was excluded because there were no relevant data. We found that the modeled lncRNAs were significantly different between normal and tumor cells (P < 0.05). CTD-2066L21.3, LINC00355, CTD-2555C10.3, OGFRP1, and LINC00862 were found to be substantially expressed in tumor cells but expressed at low levels in normal cells. Other lncRNAs were found to be highly expressed in normal cells but expressed at low levels in tumor cells. This demonstrates the expression signature of the lncRNAs in our model (Fig. 8B).

## Discussion

Gefitinib is effective in the treatment of EGFR-mutant lung adenocarcinoma, but its metabolic effects on lung cancer remain unclear. As a biomarker, numerous studies have demonstrated a causal connection among lncRNAs and the beginning and progression of many forms of cancer. Meanwhile, many studies have proven that lncRNAs have a certain influence on the treatment of gefitinib^22,23^. However, no lncRNAs related to gefitinib metabolism have been identified in LUAD. In our research, the significance of lncRNA prognostic models concerning gefitinib metabolism in LUAD patients was demonstrated. We constructed a risk assessment model containing 9 gefitinib metabolism-related lncRNAs and constructed a nomogram that can strongly predict an accurate prognosis. GO analysis, KEGG analysis, and GSEA confirmed that the metabolism of gefitinib was related to cytochrome P450 and lipid metabolism and also affects the development of drug resistance. Evasion of Immunity and Immunotherapy analysis demonstrated that immune evasion is more likely with an increased risk score. Cancer microenvironment analysis found that most immune cells were present in higher concentrations in the low-risk subgroup. Drug susceptibility research found 23 sensitive drugs, 21 of which were more sensitive in high-risk groups.

In this study, a gefitinib metabolism-related prediction model and nomogram were constructed, demonstrating high predictive ability, as evidenced by various evaluation metrics, such as the ROC curve, C-index curve, and calibration curve. Comparisons with other studies and models revealed its superior performance. For instance, the developed model achieved a higher C-index (0.780) than the model in the study by Lin et al. (0.711)^24^. Furthermore, the gefitinib metabolism-related prediction model outperformed the lung adenocarcinoma risk model studied by Ma C et al. and exhibited superior predictive ability compared to the model investigated by Ren et al. In these studies, the risk model demonstrated higher AUC values (training group > 0.7, verification group > 0.6)^25,26^. Although the accuracy for the 5-year period was slightly lower than that of the model by Li et al., it showed higher accuracy for the 1- and 3-year periods^27^. Meanwhile, two genes associated with lung cancer, namely, LINC00355 and OGFRP1, were identified by the developed model. LINC00355 was previously reported by Yuan et al. to promote the proliferation of LUAD cells, while OGFRP1 was found by Xiaojing Liu et al. acting as a carcinogen in NSCLC^28,29^. Moreover, the model incorporated a larger number of genes with a significant influence on the prognosis of lung adenocarcinoma, encompassing both risk factors (e.g., RP11.345M22.2) and protective factors (e.g., LINC00355). The model's efficacy was further validated by PCA, which effectively distinguished patients into two subgroups due to the selected lncRNA. In summary, the gefitinib metabolism-related prediction model and nomogram developed in this study exhibited exceptional predictive accuracy, surpassing existing models in certain scenarios and identifying novel potential targets for further investigation.

Early tumors and advanced tumors were shown to be strongly related to low- and high-risk ratings, respectively. Gefitinib metabolism is related to lipid metabolism, according to GO and KEGG pathway enrichment studies. (GO:0006650, GO:0046486, GO:0009062, hsa00071, hsa00564). Liao T et al. found that in NSCLC, fasudil can increase gefitinib sensitivity by reducing intracellular lipid accumulation^30^, while a diet rich in lipids can promote tumor development^31^. Moreover, abnormal accumulation of intracellular lipids is one of the reasons for gefitinib resistance^32^. In the future, drugs that affect lipid metabolism may be used to enhance the efficacy of gefitinib. In addition, the enrichment results found a variety of pathways that may affect drug resistance, such as NF-kappaB pathway and JNK cascade^33,34^. And GSEA proved that these pathways were enriched in the high-risk group. At the same time, GSEA revealed that cytochrome P450 is involved in the metabolism of gefitinib in lung cancer^35^. In particular, the metabolic ability of cytochrome P450 1A1 (CYP1A1) in lung cancer tumor cells affects the efficacy of gefitinib^36^. Moreover, cytochrome P450-related pathways were also enriched in the high-risk group. Therefore, we believe that the metabolic process of gefitinib may contribute to gefitinib resistance to a certain extent through certain mechanisms. And the high-risk group with stronger metabolic capacity are more likely to develop drug resistance. The connection between the metabolism of lipids and gefitinib metabolism was also further validated. Additionally, multiple tumor-promoting pathways were identified in the high-risk subgroup, providing more directions for further study in LUAD. Multiple tumor immune microenvironment variables were shown to be strongly associated with the risk model. T-cell costimulation and HLA were identified as low-risk pathways, as found in the study of cuproptosis and hepatocellular carcinoma by Shu Jia Chen et al., which was consistent with our findings^37^. The risk score was determined to have a substantial positive relationship with the MDSC, CAF, and exclusion variables. Conversely, there was an unfavorable connection observed between IFNG and the risk score. Relevant studies have proven that MDSC and CAF are involved in the progression and immune suppression of lung cancer. This confirms that the high risk score of our model is associated with worse prognosis^38,39^. Although there was no significant difference in TMB between the low-risk and high-risk subgroups, a higher TMB value indicated a better prognosis. The expression properties of the modeled lncRNAs in tumor and normal cells were validated by RT-qPCR tests.

Next, we predicted potential chemotherapeutic agents that could be used to treat LUAD patients, 21 of which had higher sensitivity levels for patients in the high-risk subgroup. The 5 most relevant drugs caught our attention. Among them, AZD7762, in combination with checkpoint kinase 1 (CHK1) inhibitors, has shown potential for treating triple-negative breast cancer^40^; BMS-754807 can be combined with dasatinib to suppress the growth of lung cancer cells and produce synergistic cytotoxicity^41^; luminespib may be a potential drug for EGFR in 20 advanced NSCLC patients’ treatment^42^; staurosporine was found that its drug sensitivity is negatively correlated with the expression of EGFR^43^; camptothecin is a promising anti-obesity agent by activating the GDF15-GFRAL pathway^44^. Our study found that among the low expression and high expression groupings, there was no discernible link in the OS of the stem cell index, while the clinical features of LUAD were highly connected with mRNAsi, which indicated that mRNAsi was linked to patients' prognoses.

There are several merits in our study. The gefitinib metabolism-related prediction model and nomogram developed in this study demonstrated superior performance compared to lung adenocarcinoma risk models, achieving higher AUC values and C-index. The model's consistent results across different studies indicate its robustness and reliability. By identifying two lung cancer-associated genes and incorporating influential genes, the model offers valuable insights for further research and potential therapeutic targets. Overall, this study provides a highly accurate and comprehensive predictive tool that surpasses existing models, opening new avenues for studying lung adenocarcinoma. Nevertheless, it is crucial to note the limitations of our research, including the reliance on public data and the need for verification through experimental and clinical studies. Additionally, specific signaling pathways related to LUAD growth were not identified within the scope of our research.

## Conclusion

The prediction model and nomogram we developed based on nine gefitinib metabolism-related lncRNAs showed superior prediction accuracy and potential for further research. Furthermore, it highlights the association between gefitinib and lipid metabolism and identifies pathways associated with tumor development in high-risk populations, providing valuable direction for further research on LUAD. We also identified potential chemotherapeutic agents for LUAD treatment, such as BMS-754807, Luminespi, and staurosporine, which have shown potential in lung cancer in multiple studies. Due to the limitations of this study, we need to conduct basic experiments to verify the relevant mechanism, and the choice of chemotherapy drugs needs to be confirmed by clinical practice.

### Supplementary Information

Supplementary Figure S1.

Supplementary Figure S2.

Supplementary Figure S3.

Supplementary Figure S4.

Supplementary Figure S5.

Supplementary Figure S6.

Supplementary Figure S7.

Supplementary Figure S8.

Supplementary Figure S9.

Supplementary Figure S10.

Supplementary Figure S11.

Supplementary Figure S12.

Supplementary Figure S13.

Supplementary Figure S14.

Supplementary Table S1.

Supplementary Table S2.

Supplementary Table S3.

Supplementary Table S4.

Supplementary Table S5.

Supplementary Table S6.

Supplementary Table S7.

Supplementary Table S8.

Supplementary Table S9.

Supplementary Legends.

## Data Availability

The data sets used and/or analyzed during the current study are available from the corresponding author on reasonable request.

## Abbreviations

AUC: Area under the curve
Coef: Coefficient
FDR: False discovery rate
GMLncs: Gefitinib metabolism-related lncRNAs
GO: Gene ontology
GSEA: Gene set enrichment
GMLncSig: Gefitinib metabolism-related lncRNA gene signature
KM survival analysis: Kaplan–Meier survival analysis
KEGG: Kyoto Encyclopedia of Genes and Genomes
lncRNA: Long non-coding RNA
LUAD: Lung adenocarcinoma
OS: Overall survival
PCA: Principal component analysis
RT-qPCR: Real-time quantitative polymerase chain reaction
ROC Analysis: Time receiver operating characteristic analysis
ssGSEA: Single-sample gene set enrichment analysis
TCGA: The Cancer Genome Atlas
TIDE: Tumor immune dysfunction and exclusion
mRNAsi: Stem cell index
TMB: Tumor mutational burden
