# Supplementary figures and images for: Gefitinib metabolism-related lncRNAs for the prediction of prognosis, tumor microenvironment and drug sensitivity in lung adenocarcinoma
Source: Sci Rep. 2024 May 6;14:10348. doi: 10.1038/s41598-024-61175-3 (PMC11074108; doi:10.1038/s41598-024-61175-3)

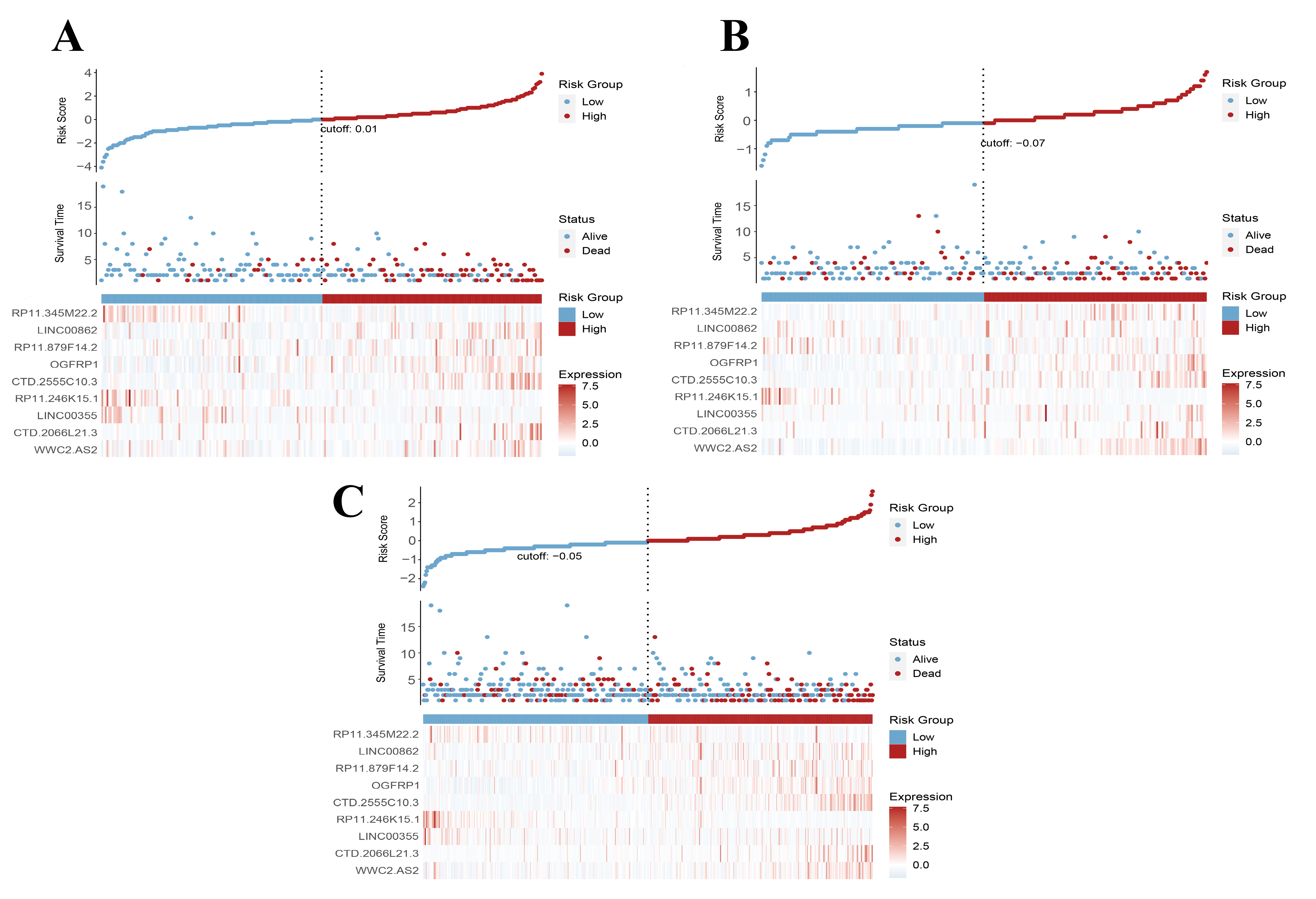

Supplement: Supplementary file 2 — Supplementary Figure S2. [file 41598_2024_61175_MOESM2_ESM.tif]

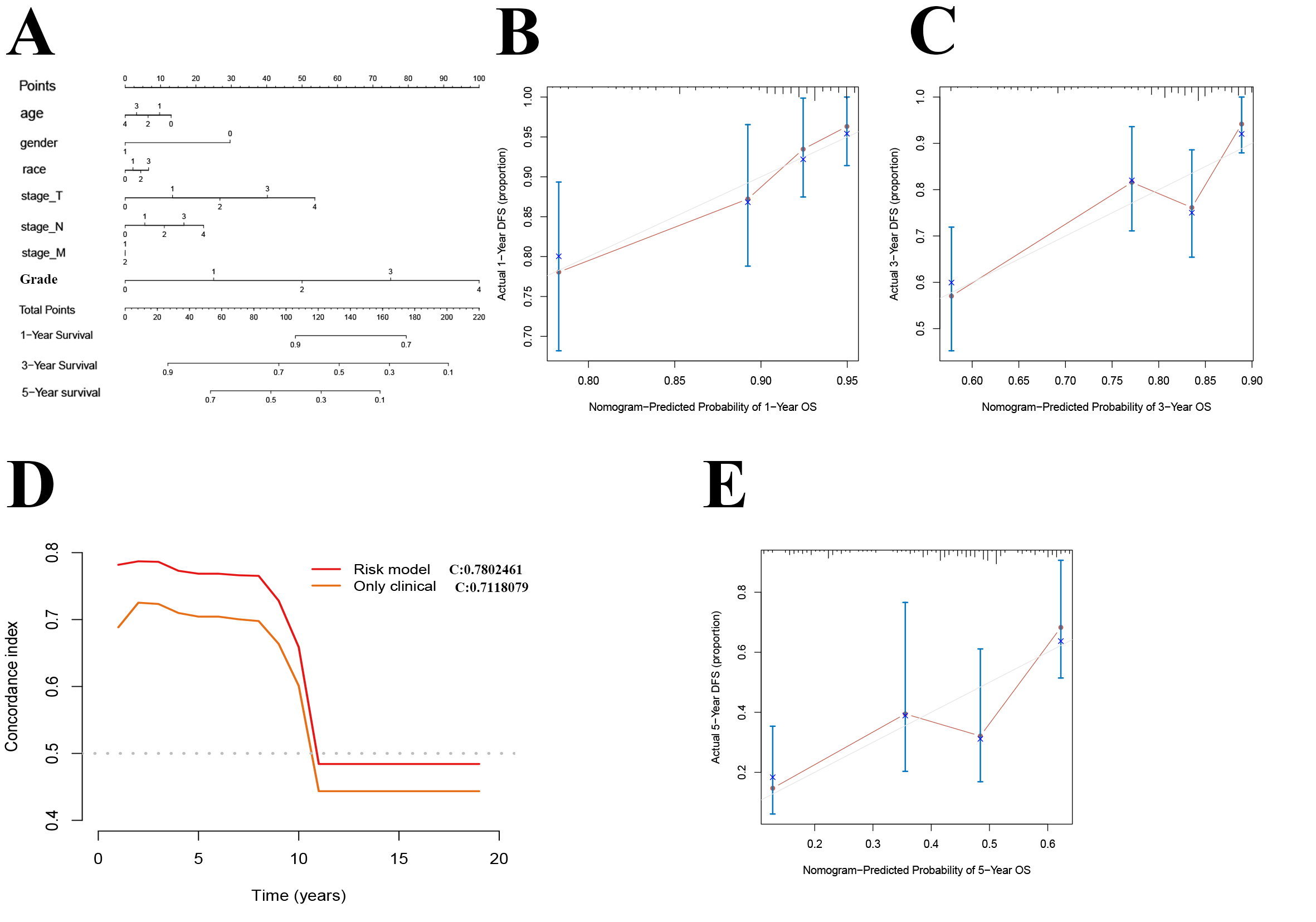

Supplement: Supplementary file 3 — Supplementary Figure S3. [file 41598_2024_61175_MOESM3_ESM.tif]

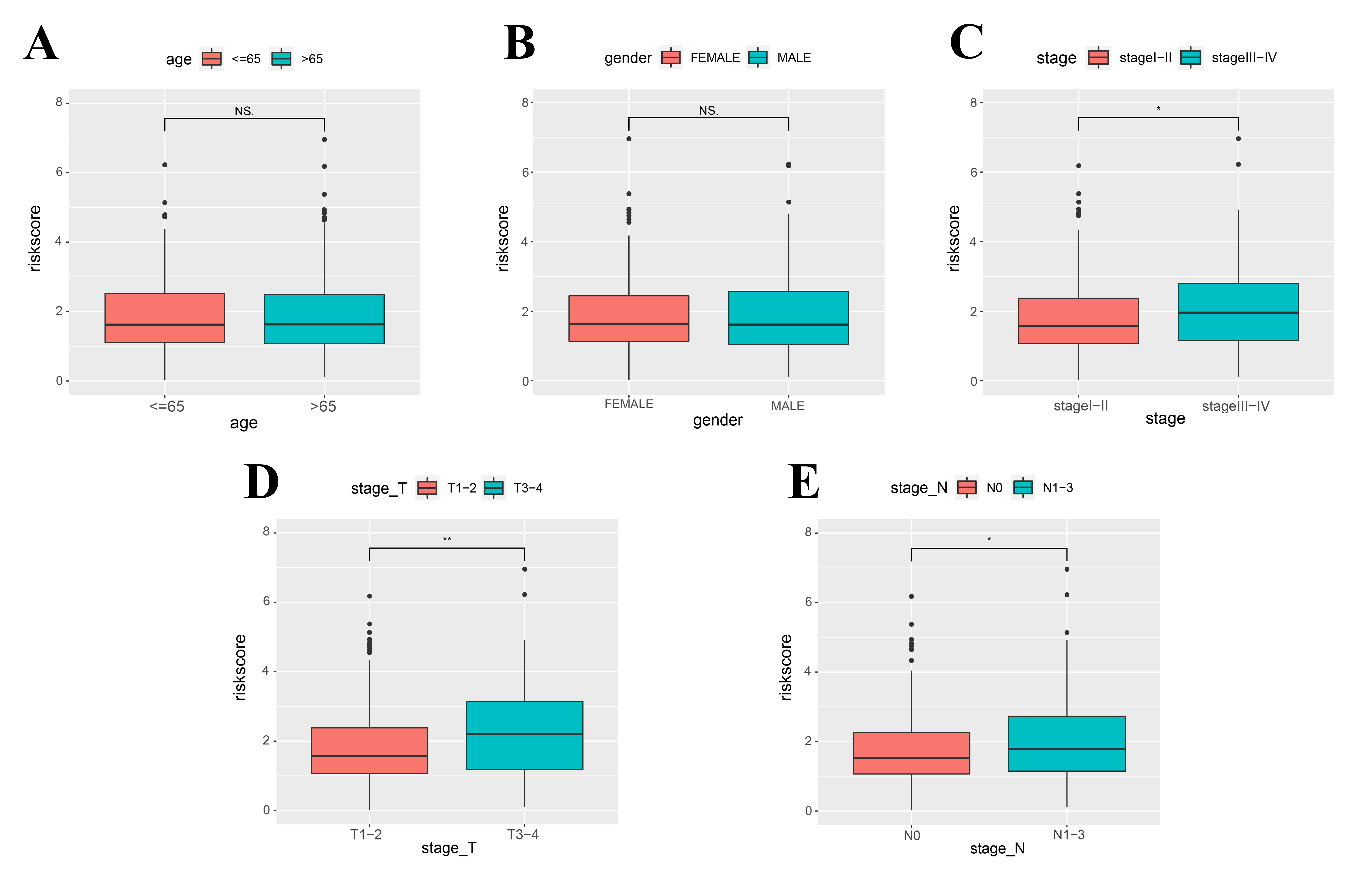

Supplement: Supplementary file 4 — Supplementary Figure S4. [file 41598_2024_61175_MOESM4_ESM.tif]

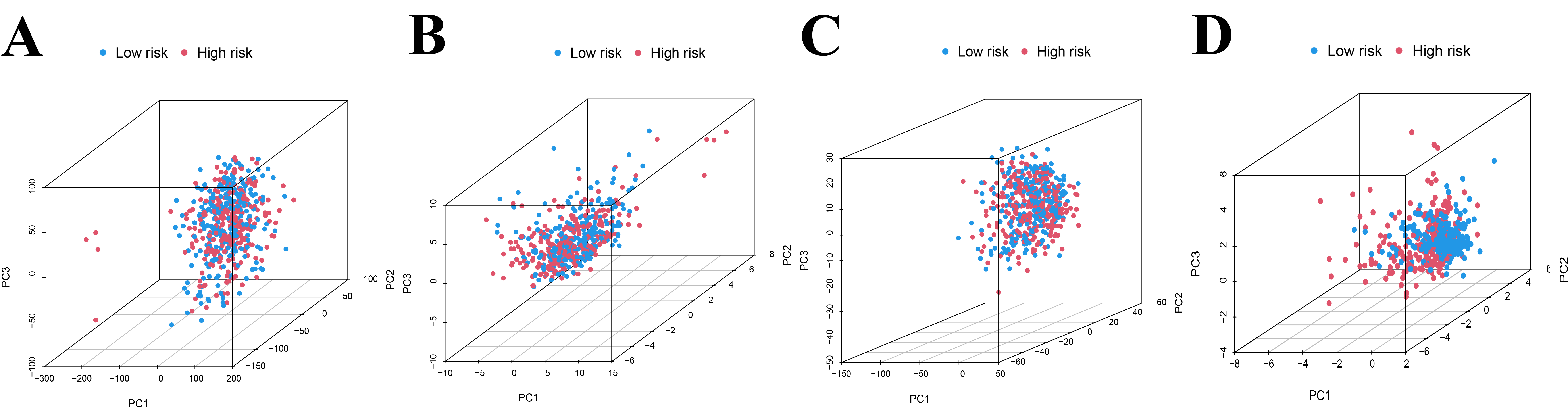

Supplement: Supplementary file 6 — Supplementary Figure S6. [file 41598_2024_61175_MOESM6_ESM.tif]

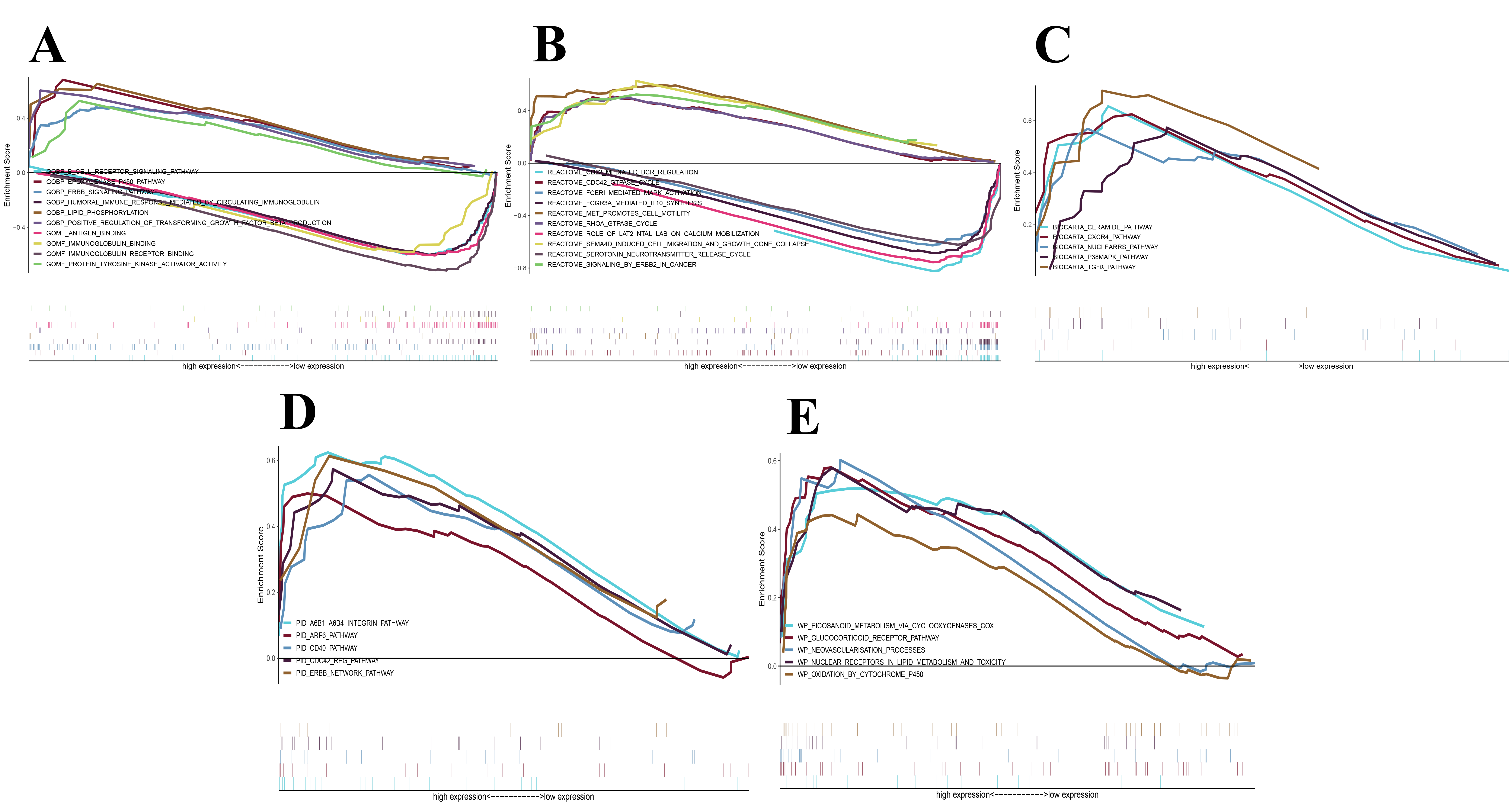

Supplement: Supplementary file 7 — Supplementary Figure S7. [file 41598_2024_61175_MOESM7_ESM.tif]

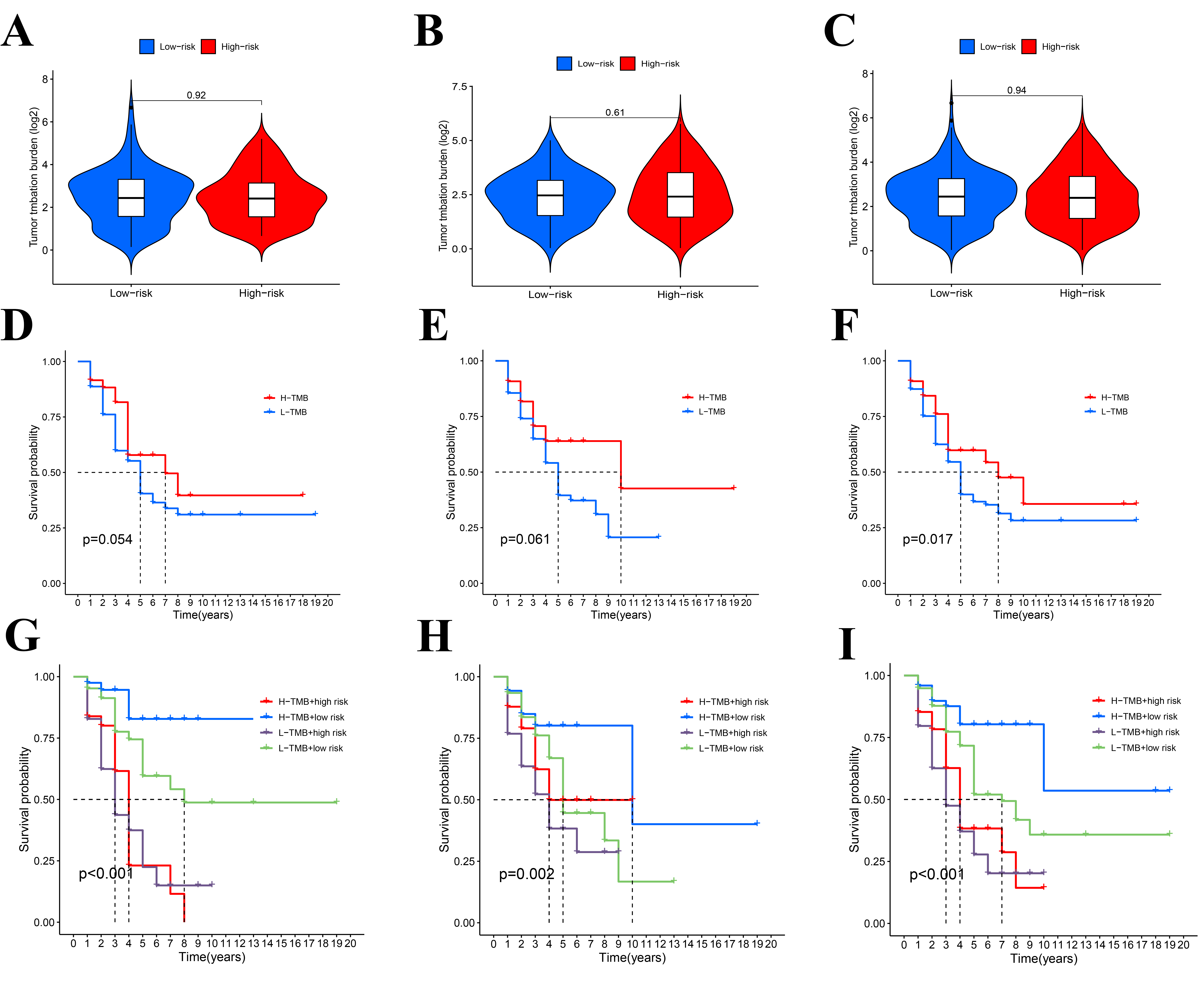

Supplement: Supplementary file 11 — Supplementary Figure S11. [file 41598_2024_61175_MOESM11_ESM.tif]

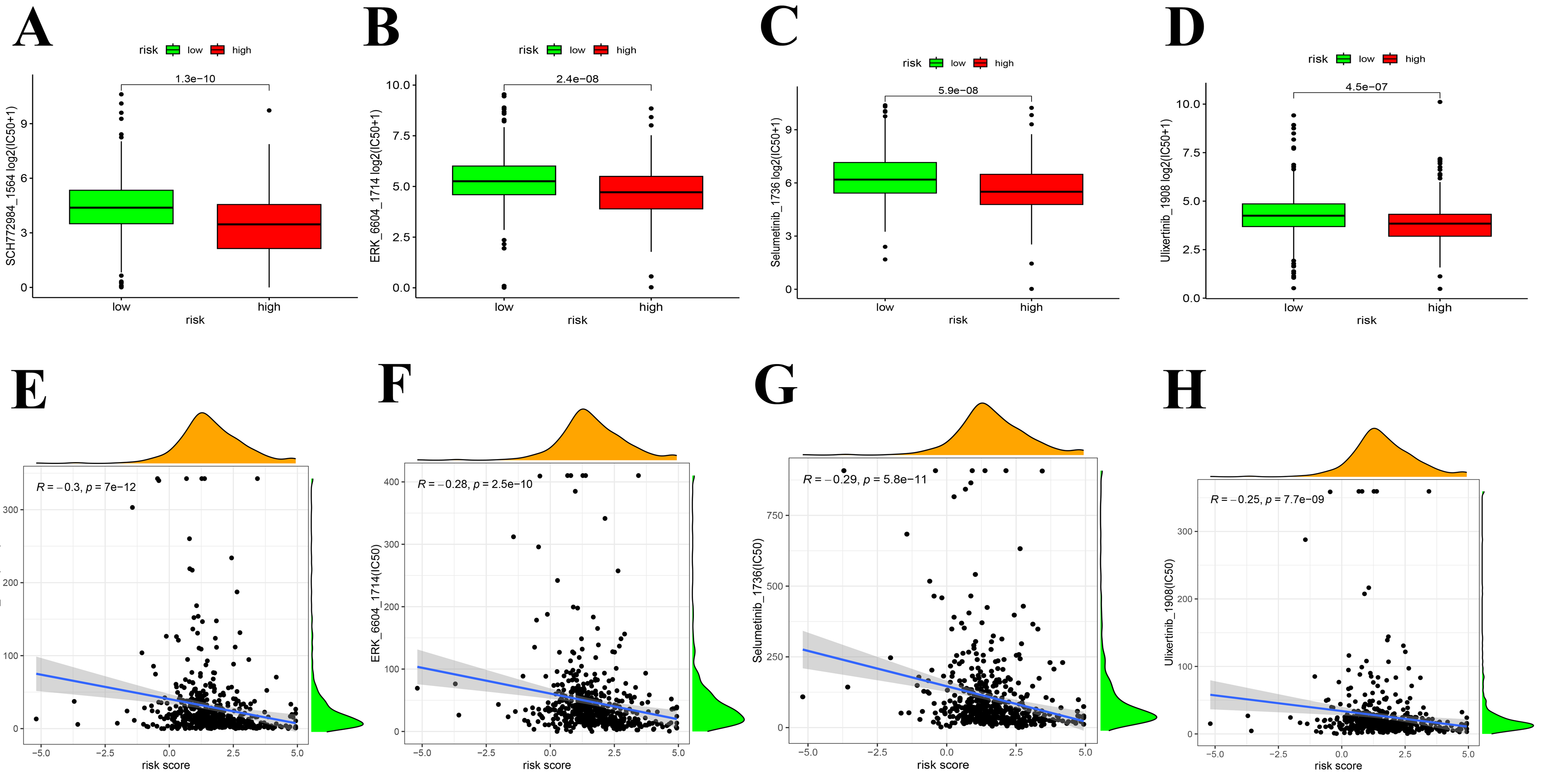

Supplement: Supplementary file 12 — Supplementary Figure S12. [file 41598_2024_61175_MOESM12_ESM.tif]

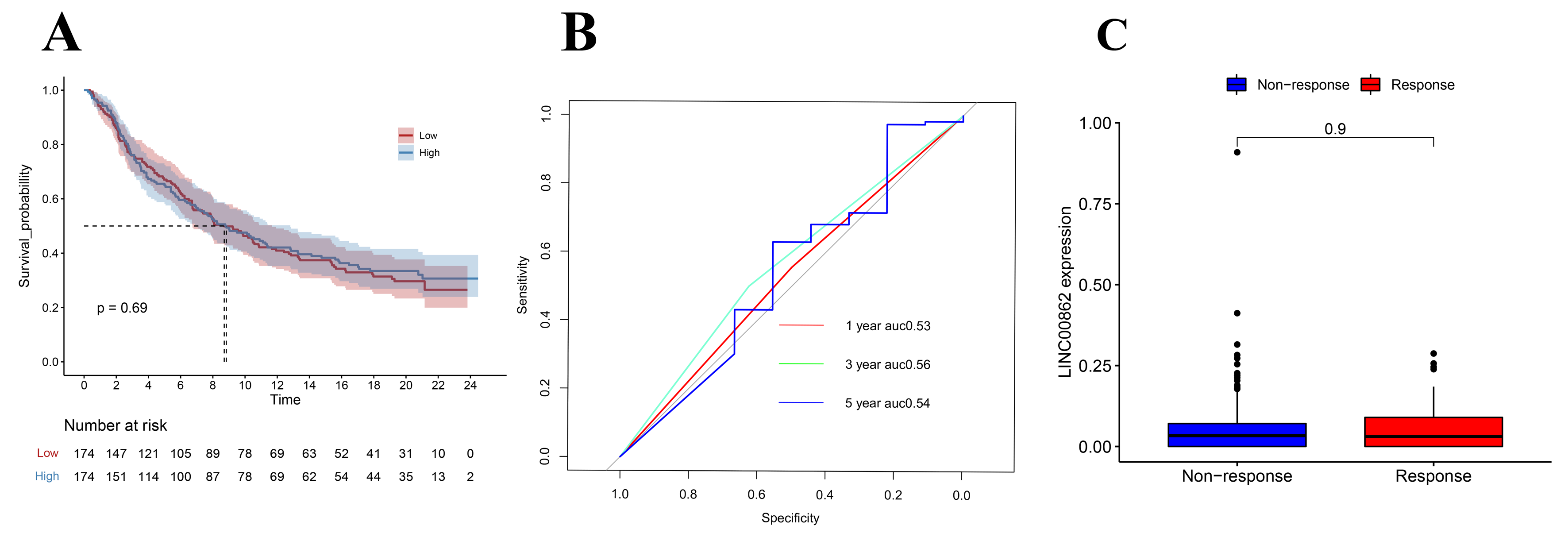

Supplement: Supplementary file 13 — Supplementary Figure S13. [file 41598_2024_61175_MOESM13_ESM.tif]

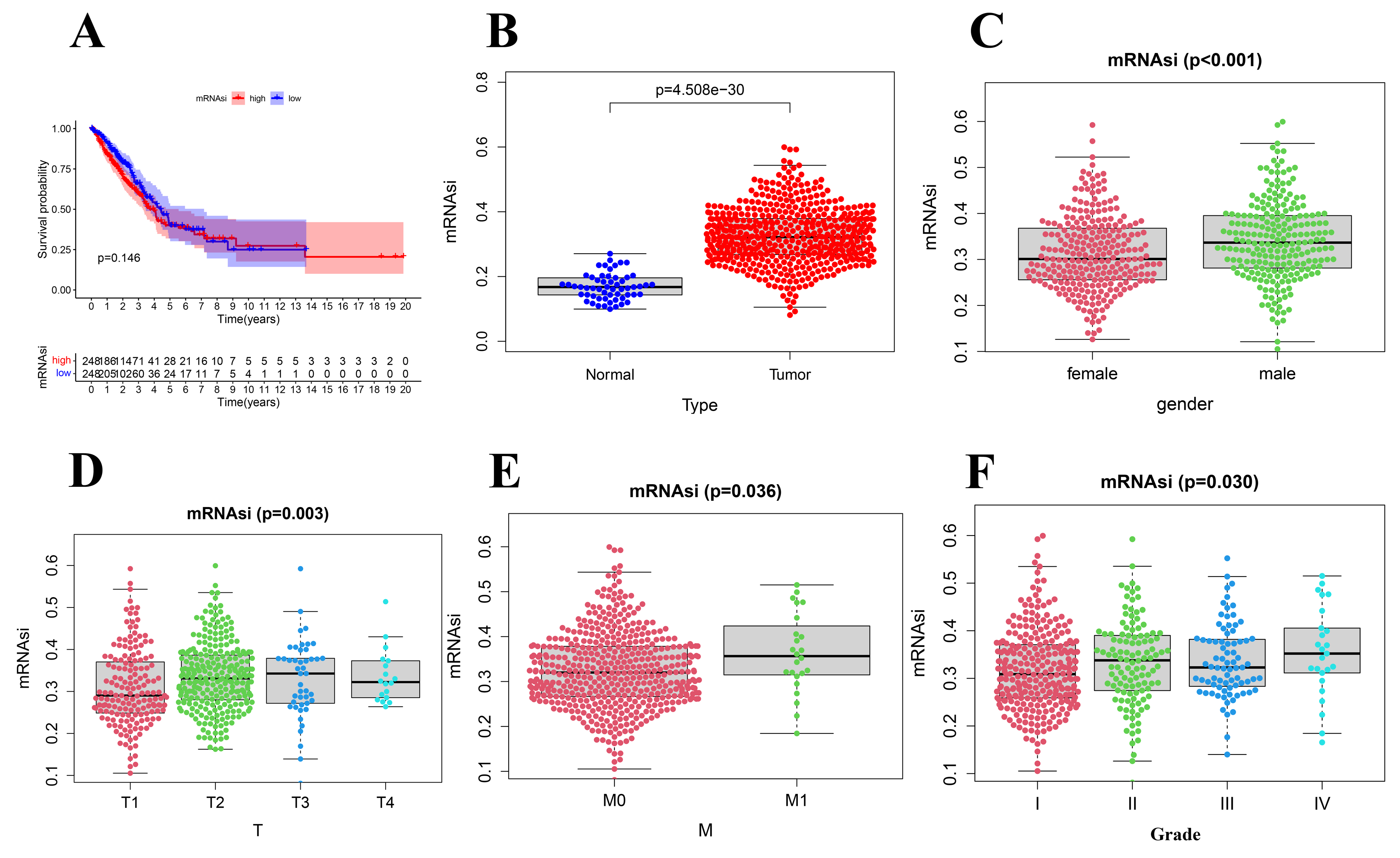

Supplement: Supplementary file 14 — Supplementary Figure S14. [file 41598_2024_61175_MOESM14_ESM.tif]
