# Supplementary material for: Gefitinib metabolism-related lncRNAs for the prediction of prognosis, tumor microenvironment and drug sensitivity in lung adenocarcinoma
Source: Sci Rep. 2024 May 6;14:10348. doi: 10.1038/s41598-024-61175-3 (PMC11074108; doi:10.1038/s41598-024-61175-3)
Supplement: Supplementary file 15 — Supplementary Table S1. [file 41598_2024_61175_MOESM15_ESM.docx]

**Table S1** Primer sequences for seven GMLncs.

| **Gene** | **Primer F** | **Primer R** |
| --- | --- | --- |
| β-actin | CATCCGCAAAGACCTGTACG | CCTGCTTGCTGATCCACATC |
| WWC2-AS2 | GGGTCGTGTTTGCCCTTAGA | CTAAATGCGGTCAAAGCGGG |
| CTD-2066L21.3 | GCCTAGCTTGGTGCAAACAC | CCCTGCTGCACTTGGATGTA |
| LINC00355 | TGTCCCTGGGGCTGAGAATA | TGACAGGTAGGTGGCATGTG |
| RP11-246K15.1 | TTCACTTGGCTGTATGGCCT | GCCCCACAGTGAGGTTGTAG |
| CTD-2555C10.3 | CTTCGAACCTCTGGGCCTTT | CGGTCCCGGATTTAAGCAGA |
| OGFRP1 | CAACTTTCTGGGGGAGTTGGT | AAACAGGTCACCGAGAGGAGA |
| LINC00862 | TCACAGGGAAGTCGGAAAGC | CATCCACGAAGGGAGCAAGT |
| RP11-879F14.2 | AGGCCCTTTACAGACTCCCT | TTCTTGTCGTCAGTCACGGG |
| RP11-345M22.2 | TTTCCCCCTCACGCAAATGT | AATTGTGAGAGGTGGCCCAG |

**Abbreviations:** GMLncs: Gefitinib metabolism-related long non-coding RNA.
