# Supplementary material for: Gefitinib metabolism-related lncRNAs for the prediction of prognosis, tumor microenvironment and drug sensitivity in lung adenocarcinoma
Source: Sci Rep. 2024 May 6;14:10348. doi: 10.1038/s41598-024-61175-3 (PMC11074108; doi:10.1038/s41598-024-61175-3)
Supplement: Supplementary file 16 — Supplementary Table S2. [file 41598_2024_61175_MOESM16_ESM.docx]

**Table S2** The specific information of 128 GMlncs screened after univariate cox analysis on Train cohort.

| **Gene** | **HR** | **Lower** | **Upper** | **P-value** |
| --- | --- | --- | --- | --- |
| COLCA1 | 0.73 | 0.57 | 0.93 | 0.01 |
| WWC2.AS2 | 4.6 | 1.5 | 14 | 0.0068 |
| CTD.2066L21.3 | 2.2 | 1.5 | 3.2 | 0.00004 |
| RP11.417E7.1 | 1.6 | 1 | 2.5 | 0.038 |
| RP1.15D23.2 | 0.00091 | 0.0000067 | 0.12 | 0.0052 |
| AC128709.2 | 0.08 | 0.0065 | 0.98 | 0.048 |
| MID1IP1.AS1 | 0.63 | 0.43 | 0.9 | 0.012 |
| RP11.283G6.4 | 0.51 | 0.28 | 0.91 | 0.022 |
| RP5.907D15.4 | 2.2 | 1.2 | 3.9 | 0.01 |
| RP11.401P9.4 | 0.69 | 0.48 | 0.99 | 0.041 |
| RP11.124O11.1 | 0.0011 | 0.0000025 | 0.46 | 0.027 |
| BLACAT1 | 1.3 | 1 | 1.7 | 0.033 |
| C6orf3 | 0.42 | 0.18 | 0.99 | 0.047 |
| RP11.295G20.2 | 1.2 | 1 | 1.4 | 0.031 |
| RP11.38M8.1 | 1.6 | 1.1 | 2.2 | 0.0061 |
| RP3.525N10.2 | 0.16 | 0.03 | 0.87 | 0.034 |
| CTD.2589M5.4 | 0.79 | 0.66 | 0.94 | 0.009 |
| LINC01028 | 180 | 3.6 | 9000 | 0.0093 |
| ARHGEF26.AS1 | 0.63 | 0.42 | 0.94 | 0.026 |
| RP11.863K10.7 | 0.024 | 0.00084 | 0.68 | 0.029 |
| RP11.320N7.2 | 0.79 | 0.64 | 0.99 | 0.045 |
| RP11.418I22.2 | 0.018 | 0.00043 | 0.74 | 0.034 |
| C14orf132 | 0.73 | 0.55 | 0.98 | 0.039 |
| LINC01234 | 1.7 | 1.2 | 2.4 | 0.0048 |
| RP11.886D15.1 | 0.000000025 | 9.7E-15 | 0.066 | 0.02 |
| LINC00704 | 1.8 | 1.3 | 2.7 | 0.0015 |
| RP11.416I2.1 | 1.7 | 1 | 2.8 | 0.033 |
| RP11.429J17.5 | 1.4 | 1 | 1.8 | 0.021 |
| LINC01537 | 3.8 | 1.5 | 9.7 | 0.0058 |
| LINC00519 | 1.7 | 1.1 | 2.5 | 0.013 |
| AP000695.6 | 1.7 | 1.1 | 2.5 | 0.01 |
| RP11.514D23.2 | 0.018 | 0.00064 | 0.49 | 0.017 |
| LINC00891 | 0.027 | 0.001 | 0.71 | 0.03 |
| LINC00355 | 0.6 | 0.37 | 0.99 | 0.046 |
| RP11.80H5.2 | 3.4 | 1 | 12 | 0.048 |
| RP11.359G22.2 | 29 | 3.2 | 250 | 0.0027 |
| RP11.133F8.2 | 0.019 | 0.0012 | 0.29 | 0.0047 |
| RP11.7K24.3 | 1.3 | 1 | 1.8 | 0.047 |
| RP11.246K15.1 | 0.19 | 0.044 | 0.77 | 0.021 |
| RP11.141M1.3 | 0.067 | 0.0082 | 0.55 | 0.012 |
| RP11.95I16.6 | 0.55 | 0.33 | 0.94 | 0.029 |
| CTC.548K16.1 | 1.3 | 1 | 1.7 | 0.035 |
| AC128709.3 | 0.17 | 0.03 | 1 | 0.049 |
| LINC01468 | 1.5 | 1.2 | 1.9 | 0.0021 |
| RP11.434D9.1 | 0.31 | 0.11 | 0.92 | 0.035 |
| RP11.33A14.1 | 0.0033 | 0.000028 | 0.39 | 0.019 |
| RP11.370I10.2 | 0.23 | 0.065 | 0.82 | 0.024 |
| RP11.1042B17.3 | 0.26 | 0.07 | 0.97 | 0.045 |
| RP11.554I8.2 | 1.4 | 1.1 | 1.6 | 0.00041 |
| MED4.AS1 | 0.41 | 0.17 | 0.98 | 0.044 |
| CTD.2510F5.4 | 1.3 | 1 | 1.5 | 0.018 |
| LINC00908 | 0.33 | 0.12 | 0.87 | 0.026 |
| AC005355.2 | 1.5 | 1.2 | 2 | 0.0012 |
| RP11.379K22.3 | 1.3 | 1 | 1.5 | 0.016 |
| FAM83H.AS1 | 1.3 | 1 | 1.6 | 0.035 |
| RP11.456K23.1 | 0.45 | 0.23 | 0.88 | 0.02 |
| CTD.2524L6.3 | 0.14 | 0.028 | 0.69 | 0.016 |
| CTD.2016O11.1 | 1.9 | 1 | 3.5 | 0.037 |
| RP11.359N11.1 | 0.29 | 0.092 | 0.9 | 0.033 |
| CASC8 | 1.6 | 1.2 | 2.2 | 0.0015 |
| AC000403.4 | 0.24 | 0.062 | 0.9 | 0.035 |
| RP11.1293J14.1 | 0.59 | 0.37 | 0.95 | 0.029 |
| AP000695.4 | 2 | 1.3 | 3.2 | 0.003 |
| AC018647.3 | 0.04 | 0.0041 | 0.4 | 0.0059 |
| CH17.360D5.3 | 1.4 | 1.1 | 1.8 | 0.012 |
| A2M.AS1 | 0.38 | 0.16 | 0.88 | 0.025 |
| Z83851.4 | 1.9 | 1.2 | 2.8 | 0.0033 |
| RP11.132A1.4 | 1.3 | 1 | 1.5 | 0.022 |
| LINC00628 | 2.2 | 1.1 | 4.5 | 0.035 |
| RP11.1029J19.4 | 1.4 | 1 | 1.9 | 0.04 |
| RP11.66N24.6 | 0.85 | 0.73 | 0.99 | 0.034 |
| AC124789.1 | 1.6 | 1 | 2.4 | 0.032 |
| AP000438.2 | 0.022 | 0.0011 | 0.43 | 0.012 |
| LINC01268 | 0.39 | 0.16 | 0.95 | 0.038 |
| CTD.2357A8.3 | 1.4 | 1.2 | 1.8 | 0.0011 |
| CTD.3179P9.1 | 0.012 | 0.00019 | 0.71 | 0.034 |
| LINC01354 | 0.11 | 0.014 | 0.91 | 0.04 |
| RP1.159A19.4 | 0.54 | 0.32 | 0.91 | 0.02 |
| RP11.10A14.5 | 1.3 | 1.1 | 1.6 | 0.00053 |
| AC109642.1 | 0.64 | 0.43 | 0.96 | 0.033 |
| RP11.284F21.10 | 1.2 | 1 | 1.4 | 0.013 |
| RP11.739N20.2 | 1.4 | 1.1 | 1.9 | 0.0057 |
| LINC00659 | 1.6 | 1.2 | 2.3 | 0.004 |
| RP11.434I12.3 | 0.79 | 0.62 | 0.99 | 0.042 |
| LINC00707 | 1.5 | 1.2 | 2 | 0.0016 |
| RP11.284F21.7 | 1.2 | 1 | 1.5 | 0.011 |
| CTD.2555C10.3 | 2.4 | 1.7 | 3.2 | 8.6E-08 |
| MIR99AHG | 0.41 | 0.19 | 0.89 | 0.024 |
| RP3.523K23.2 | 1.3 | 1.1 | 1.6 | 0.0053 |
| RP11.96H17.1 | 2.1 | 1.1 | 4.1 | 0.034 |
| MNX1.AS1 | 1.3 | 1 | 1.6 | 0.017 |
| OGFRP1 | 2.6 | 1.4 | 4.8 | 0.0033 |
| RP11.213H15.1 | 0.44 | 0.25 | 0.79 | 0.006 |
| VPS9D1.AS1 | 1.3 | 1.1 | 1.6 | 0.016 |
| LINC00261 | 0.82 | 0.68 | 0.98 | 0.032 |
| RP11.539E17.5 | 2.7 | 1.8 | 4.2 | 0.0000037 |
| CTD.2353F22.1 | 0.56 | 0.32 | 0.98 | 0.044 |
| RP11.635O16.2 | 0.64 | 0.41 | 0.99 | 0.043 |
| RP11.378A13.1 | 0.51 | 0.28 | 0.95 | 0.033 |
| CH17.360D5.2 | 1.4 | 1 | 2 | 0.027 |
| RP11.264B14.1 | 0.4 | 0.17 | 0.95 | 0.039 |
| RP11.23D24.2 | 0.18 | 0.041 | 0.75 | 0.019 |
| RP11.368L12.1 | 1.8 | 1.1 | 3.1 | 0.028 |
| LINC00968 | 0.29 | 0.11 | 0.76 | 0.012 |
| RP1.90J4.1 | 0.39 | 0.17 | 0.89 | 0.025 |
| RP11.462L8.1 | 1.5 | 1.3 | 1.8 | 0.000012 |
| RP4.639F20.1 | 0.74 | 0.56 | 0.98 | 0.038 |
| RP11.344B5.2 | 0.68 | 0.53 | 0.88 | 0.0031 |
| RP11.350J20.12 | 1.2 | 1 | 1.4 | 0.048 |
| RP11.211G23.2 | 1.3 | 1.1 | 1.5 | 0.0019 |
| LANCL1.AS1 | 0.49 | 0.26 | 0.9 | 0.022 |
| RP11.879F14.2 | 3.5 | 1.3 | 9 | 0.011 |
| LINC00857 | 1.7 | 1.2 | 2.4 | 0.0014 |
| RP11.1060J15.9 | 2.5 | 1.1 | 6 | 0.036 |
| LINC01352 | 0.16 | 0.041 | 0.65 | 0.01 |
| LINC00460 | 1.2 | 1 | 1.4 | 0.014 |
| RP11.807H17.1 | 1.8 | 1.1 | 3.1 | 0.022 |
| RP11.284F21.9 | 1.2 | 1 | 1.4 | 0.027 |
| LINC00862 | 1.6 | 1.1 | 2.2 | 0.0084 |
| AC145124.2 | 0.29 | 0.084 | 0.97 | 0.044 |
| RP11.21L23.2 | 1.3 | 1 | 1.7 | 0.027 |
| RP11.800A18.4 | 1.6 | 1.1 | 2.1 | 0.0061 |
| CARD8.AS1 | 0.67 | 0.45 | 0.98 | 0.041 |
| TRHDE.AS1 | 6.1 | 2.3 | 16 | 0.00034 |
| RP11.677M14.3 | 0.59 | 0.38 | 0.93 | 0.022 |
| FAM83A.AS1 | 1.5 | 1.3 | 1.8 | 0.0000014 |
| RP11.345M22.2 | 0.028 | 0.0031 | 0.25 | 0.0014 |
| ATP13A4.AS1 | 0.64 | 0.44 | 0.94 | 0.021 |

**Abbreviations:** GMLncs: gefitinib metabolism-related long noncoding RNAs; lncRNA: long non-coding RNA; P-value: Probability; HR: Hazard Ratio.
