# Supplementary material for: Gefitinib metabolism-related lncRNAs for the prediction of prognosis, tumor microenvironment and drug sensitivity in lung adenocarcinoma
Source: Sci Rep. 2024 May 6;14:10348. doi: 10.1038/s41598-024-61175-3 (PMC11074108; doi:10.1038/s41598-024-61175-3)
Supplement: Supplementary file 17 — Supplementary Table S3. [file 41598_2024_61175_MOESM17_ESM.docx]

**Table S3** Genetic information after multi-cox regression analysis.

| **Gene** | **Coef** | **HR** | **Se(coef)** | **Z** | **Pr(>\|z\|)** |
| --- | --- | --- | --- | --- | --- |
| WWC2.AS2 | 2.68 | 14.56 | 14.56 | 2.91 | 0.00 |
| CTD.2066L21.3 | 0.63 | 1.88 | 1.88 | 2.05 | 0.04 |
| LINC00355 | -1.33 | 0.27 | 0.27 | -3.85 | 0.00 |
| RP11.246K15.1 | -2.36 | 0.09 | 0.09 | -2.70 | 0.01 |
| CTD.2555C10.3 | 0.81 | 2.24 | 2.24 | 2.51 | 0.01 |
| OGFRP1 | 1.14 | 3.12 | 3.12 | 2.11 | 0.04 |
| RP11.879F14.2 | 1.92 | 6.84 | 6.84 | 2.79 | 0.01 |
| LINC00862 | 0.92 | 2.51 | 2.51 | 2.89 | 0.00 |
| RP11.345M22.2 | -2.89 | 0.06 | 0.06 | -2.03 | 0.04 |

**Abbreviations:** Coef: coefficient; HR: Hazard Ratio; Se(coef): Standard error (coefficient); Z: Z score, standard deviation; Pr>|z|: Probability>|z score|.
