# Supplementary material for: Gefitinib metabolism-related lncRNAs for the prediction of prognosis, tumor microenvironment and drug sensitivity in lung adenocarcinoma
Source: Sci Rep. 2024 May 6;14:10348. doi: 10.1038/s41598-024-61175-3 (PMC11074108; doi:10.1038/s41598-024-61175-3)
Supplement: Supplementary file 18 — Supplementary Table S4. [file 41598_2024_61175_MOESM18_ESM.docx]

| **Table S4** Related phenotypes and potential target proteins of 9 GMLncRNAs. | | |
| --- | --- | --- |
| **Gene** | **Relevant Phenotype** | **Potential Target Protein ID（TOP 10）** |
| WWC2-AS2 | protein measurement | Q9UGP8_SEC63, O14776_TCERG1, Q9BWU0_SLC4A1AP, Q5T7N2_L1TD1, O60293_ZFC3H1, Q9H501_ESF1, P54253_ATXN1, Q9UIF8_BAZ2B, O43719_HTATSF1, Q15022_SUZ12 |
| CTD-2066L21.3 | body height, bmi-adjusted hip circumference, bmi-adjusted waist circumference, neutrophil percentage of leukocytes | Q9UGP8_SEC63, P54253_ATXN1, Q9H501_ESF1, Q9BUQ8_DDX23, O14776_TCERG1, Q7KZ85_SUPT6H, Q96KR1_ZFR, P36776_LONP1, Q04637_EIF4G1, Q15022_SUZ12 |
| LINC00355 | body mass index, educational attainment, smoking initiation, takayasu arteritis, alzheimer disease | Q15022_SUZ12， P54253_ATXN1， O43290_SART1， Q9H501_ESF1， P36776_LONP1， Q9BUQ8_DDX23， Q9NRA8_EIF4ENIF1， Q7KZ85_SUPT6H， Q9UGP8_SEC63， Q04637_EIF4G1 |
| RP11-246K15.1 | nose morphology measurement, disease progression measurement, ischemic stroke | Q9UGP8_SEC63, O14776_TCERG1, Q9BWU0_SLC4A1AP, Q9H501_ESF1, Q5T7N2_L1TD1, O60293_ZFC3H1, P54253_ATXN1, Q9UIF8_BAZ2B, O43719_HTATSF1, Q15022_SUZ12 |
| CTD-2555C10.3 | platelet count, serum non-albumin protein measurement, blood protein measurement, mean platelet volume, platelet crit | O15514_POLR2D, Q86WX3_RPS19BP1, Q8IXM3_MRPL41, O43399_TPD52L2 Q13901_C1D, P62244_RPS15A, O75817_POP7, P37108_SRP14, P83876_TXNL4A, P55145_MANF |
| OGFRP1 | non-small cell lung cancer | Q9UGP8_SEC63, Q9BWU0_SLC4A1AP, O14776_TCERG1, Q5T7N2_L1TD1, O60293_ZFC3H1, Q9UIF8_BAZ2B Q9H501_ESF1, O43719_HTATSF1, P54277_PMS1, P54253_ATXN1 |
| LINC00862 | abnormality of refraction, body height, serum gamma-glutamyl transferase measurement, myopia, serum albumin measurement | Q15022_SUZ12, P54253_ATXN1, O43290_SART1, Q9H501_ESF1, Q9BUQ8_DDX23, P36776_LONP1, Q9NRA8_EIF4ENIF1, Q13206_DDX10, P49589_CARS1, Q7KZ85_SUPT6H |
| RP11-879F14.2 | Not reported | Q9UGP8_SEC63, Q9BWU0_SLC4A1AP, O14776_TCERG1, Q5T7N2_L1TD1, O60293_ZFC3H1, Q9UIF8_BAZ2B, Q9H501_ESF1, O43719_HTATSF1, P54277_PMS1, P54253_ATXN1 |
| RP11-345M22.2 | alkaline phosphatase measurement, thyroid stimulating hormone measurement, uric acid measurement, urate measurement, platelet count | Q9UGP8_SEC63, O14776_TCERG1, Q9BWU0_SLC4A1AP, Q5T7N2_L1TD1, O60293_ZFC3H1, Q9H501_ESF1, P54253_ATXN1, Q9UIF8_BAZ2B, O43719_HTATSF1, Q15022_SUZ12 |

**Abbreviation:** GMLncs: Gefitinib metabolism-related long non-coding RNA.
