# Supplementary material for: Gefitinib metabolism-related lncRNAs for the prediction of prognosis, tumor microenvironment and drug sensitivity in lung adenocarcinoma
Source: Sci Rep. 2024 May 6;14:10348. doi: 10.1038/s41598-024-61175-3 (PMC11074108; doi:10.1038/s41598-024-61175-3)
Supplement: Supplementary file 19 — Supplementary Table S5. [file 41598_2024_61175_MOESM19_ESM.docx]

**Table S5** Results of independent prognostic analyses.

| **ID** | **Univariate Cox regression** | | | |  | **Multivariate Cox regression** | | | |
| --- | --- | --- | --- | --- | --- | --- | --- | --- | --- |
|  | **HR** | **HR(95%L)** | **HR(95%H)** | **P-value** |  | **HR** | **HR(95%L)** | **HR(95%H)** | **P-value** |
| **Age** | 1.13 | 0.91 | 1.40 | 0.28 |  | 1.24 | 0.99 | 1.55 | 0.06 |
| **Gender** | 1.66 | 1.10 | 2.50 | 0.02 |  | 1.58 | 1.04 | 2.40 | 0.03 |
| **Race** | 0.82 | 0.60 | 1.11 | 0.19 |  | 0.73 | 0.53 | 1.01 | 0.06 |
| **Stage_T** | 1.48 | 1.13 | 1.94 | 0.00 |  | 1.05 | 0.79 | 1.41 | 0.72 |
| **Stage_N** | 1.58 | 1.24 | 2.02 | 0.00 |  | 1.22 | 0.88 | 1.69 | 0.23 |
| **Stage_M** | 1.56 | 1.03 | 2.35 | 0.03 |  | 0.98 | 0.61 | 1.55 | 0.92 |
| **Stage** | 1.60 | 1.32 | 1.95 | 0.00 |  | 1.44 | 1.06 | 1.95 | 0.02 |
| **Riskscore** | 1.29 | 1.11 | 1.51 | 0.00 |  | 1.32 | 1.12 | 1.55 | 0.00 |

**Abbreviations:** HR: Hazard Ratio; 95%L: 95% low; 95%H: 95% high; P-value: Probability.
