# Supplementary material for: Gefitinib metabolism-related lncRNAs for the prediction of prognosis, tumor microenvironment and drug sensitivity in lung adenocarcinoma
Source: Sci Rep. 2024 May 6;14:10348. doi: 10.1038/s41598-024-61175-3 (PMC11074108; doi:10.1038/s41598-024-61175-3)
Supplement: Supplementary file 20 — Supplementary Table S6. [file 41598_2024_61175_MOESM20_ESM.docx]

**Table S6** All pathways of GO enrichment analysis.

| **ID** | **Description** | **P-value** | **q-value** |
| --- | --- | --- | --- |
| GO:0010498 | proteasomal protein catabolic process | 0.00 | 0.00 |
| GO:0045785 | positive regulation of cell adhesion | 0.00 | 0.00 |
| GO:0016570 | histone modification | 0.00 | 0.00 |
| GO:0022411 | cellular component disassembly | 0.00 | 0.00 |
| GO:0043161 | proteasome-mediated ubiquitin-dependent protein catabolic process | 0.00 | 0.00 |
| GO:0044089 | positive regulation of cellular component biogenesis | 0.00 | 0.00 |
| GO:0034470 | ncRNA processing | 0.00 | 0.00 |
| GO:0031331 | positive regulation of cellular catabolic process | 0.00 | 0.00 |
| GO:0051251 | positive regulation of lymphocyte activation | 0.00 | 0.00 |
| GO:0050867 | positive regulation of cell activation | 0.00 | 0.00 |
| GO:0002764 | immune response-regulating signaling pathway | 0.00 | 0.00 |
| GO:0010638 | positive regulation of organelle organization | 0.00 | 0.00 |
| GO:0002696 | positive regulation of leukocyte activation | 0.00 | 0.00 |
| GO:0007015 | actin filament organization | 0.00 | 0.00 |
| GO:1903039 | positive regulation of leukocyte cell-cell adhesion | 0.00 | 0.00 |
| GO:0006260 | DNA replication | 0.00 | 0.00 |
| GO:1903829 | positive regulation of protein localization | 0.00 | 0.00 |
| GO:0043254 | regulation of protein-containing complex assembly | 0.00 | 0.00 |
| GO:0002460 | adaptive immune response based on somatic recombination of immune receptors built from immunoglobulin superfamily domains | 0.00 | 0.00 |
| GO:0016032 | viral process | 0.00 | 0.00 |
| GO:0050870 | positive regulation of T cell activation | 0.00 | 0.00 |
| GO:0042254 | ribosome biogenesis | 0.00 | 0.00 |
| GO:0018205 | peptidyl-lysine modification | 0.00 | 0.00 |
| GO:0022409 | positive regulation of cell-cell adhesion | 0.00 | 0.00 |
| GO:0010212 | response to ionizing radiation | 0.00 | 0.00 |
| GO:1903320 | regulation of protein modification by small protein conjugation or removal | 0.00 | 0.00 |
| GO:0051054 | positive regulation of DNA metabolic process | 0.00 | 0.00 |
| GO:0140694 | non-membrane-bounded organelle assembly | 0.00 | 0.00 |
| GO:0006399 | tRNA metabolic process | 0.00 | 0.00 |
| GO:0016197 | endosomal transport | 0.00 | 0.00 |
| GO:0071496 | cellular response to external stimulus | 0.00 | 0.00 |
| GO:0051052 | regulation of DNA metabolic process | 0.00 | 0.00 |
| GO:0042176 | regulation of protein catabolic process | 0.00 | 0.00 |
| GO:0016072 | rRNA metabolic process | 0.00 | 0.00 |
| GO:1902903 | regulation of supramolecular fiber organization | 0.00 | 0.00 |
| GO:0032956 | regulation of actin cytoskeleton organization | 0.00 | 0.00 |
| GO:0007249 | I-kappaB kinase/NF-kappaB signaling | 0.00 | 0.00 |
| GO:0006470 | protein dephosphorylation | 0.00 | 0.00 |
| GO:0140014 | mitotic nuclear division | 0.00 | 0.00 |
| GO:0044270 | cellular nitrogen compound catabolic process | 0.00 | 0.00 |
| GO:0034655 | nucleobase-containing compound catabolic process | 0.00 | 0.00 |
| GO:0031396 | regulation of protein ubiquitination | 0.00 | 0.00 |
| GO:0048193 | Golgi vesicle transport | 0.00 | 0.00 |
| GO:0051656 | establishment of organelle localization | 0.00 | 0.00 |
| GO:1904375 | regulation of protein localization to cell periphery | 0.00 | 0.00 |
| GO:0051258 | protein polymerization | 0.00 | 0.00 |
| GO:2001020 | regulation of response to DNA damage stimulus | 0.00 | 0.00 |
| GO:1903362 | regulation of cellular protein catabolic process | 0.00 | 0.00 |
| GO:0002443 | leukocyte mediated immunity | 0.00 | 0.00 |
| GO:0032970 | regulation of actin filament-based process | 0.00 | 0.00 |
| GO:0110053 | regulation of actin filament organization | 0.00 | 0.00 |
| GO:0016236 | macroautophagy | 0.00 | 0.00 |
| GO:0010506 | regulation of autophagy | 0.00 | 0.00 |
| GO:0061136 | regulation of proteasomal protein catabolic process | 0.00 | 0.00 |
| GO:0022604 | regulation of cell morphogenesis | 0.00 | 0.00 |
| GO:0019058 | viral life cycle | 0.00 | 0.00 |
| GO:0046700 | heterocycle catabolic process | 0.00 | 0.00 |
| GO:0033044 | regulation of chromosome organization | 0.00 | 0.00 |
| GO:0072594 | establishment of protein localization to organelle | 0.00 | 0.00 |
| GO:0097193 | intrinsic apoptotic signaling pathway | 0.00 | 0.00 |
| GO:0032535 | regulation of cellular component size | 0.00 | 0.00 |
| GO:0006302 | double-strand break repair | 0.00 | 0.00 |
| GO:0043122 | regulation of I-kappaB kinase/NF-kappaB signaling | 0.00 | 0.00 |
| GO:0006364 | rRNA processing | 0.00 | 0.00 |
| GO:0072331 | signal transduction by p53 class mediator | 0.00 | 0.00 |
| GO:1902115 | regulation of organelle assembly | 0.00 | 0.00 |
| GO:0006397 | mRNA processing | 0.00 | 0.00 |
| GO:0032984 | protein-containing complex disassembly | 0.00 | 0.00 |
| GO:0010639 | negative regulation of organelle organization | 0.00 | 0.00 |
| GO:2001233 | regulation of apoptotic signaling pathway | 0.00 | 0.00 |
| GO:0016311 | dephosphorylation | 0.00 | 0.00 |
| GO:1990778 | protein localization to cell periphery | 0.00 | 0.00 |
| GO:0030705 | cytoskeleton-dependent intracellular transport | 0.00 | 0.00 |
| GO:0006909 | phagocytosis | 0.00 | 0.00 |
| GO:0019439 | aromatic compound catabolic process | 0.00 | 0.00 |
| GO:0052126 | movement in host environment | 0.00 | 0.00 |
| GO:0046777 | protein autophosphorylation | 0.00 | 0.00 |
| GO:0007159 | leukocyte cell-cell adhesion | 0.00 | 0.00 |
| GO:0071900 | regulation of protein serine/threonine kinase activity | 0.00 | 0.00 |
| GO:0000209 | protein polyubiquitination | 0.00 | 0.00 |
| GO:0051701 | biological process involved in interaction with host | 0.00 | 0.00 |
| GO:0072659 | protein localization to plasma membrane | 0.00 | 0.00 |
| GO:0022407 | regulation of cell-cell adhesion | 0.00 | 0.00 |
| GO:0043543 | protein acylation | 0.00 | 0.00 |
| GO:0001701 | in utero embryonic development | 0.00 | 0.00 |
| GO:1903076 | regulation of protein localization to plasma membrane | 0.00 | 0.00 |
| GO:0008380 | RNA splicing | 0.00 | 0.00 |
| GO:0051092 | positive regulation of NF-kappaB transcription factor activity | 0.00 | 0.00 |
| GO:1901361 | organic cyclic compound catabolic process | 0.00 | 0.00 |
| GO:0031056 | regulation of histone modification | 0.00 | 0.00 |
| GO:0030099 | myeloid cell differentiation | 0.00 | 0.00 |
| GO:1903037 | regulation of leukocyte cell-cell adhesion | 0.00 | 0.00 |
| GO:0010508 | positive regulation of autophagy | 0.00 | 0.00 |
| GO:0002429 | immune response-activating cell surface receptor signaling pathway | 0.00 | 0.00 |
| GO:0002757 | immune response-activating signal transduction | 0.00 | 0.00 |
| GO:0032200 | telomere organization | 0.00 | 0.00 |
| GO:0045862 | positive regulation of proteolysis | 0.00 | 0.00 |
| GO:0000723 | telomere maintenance | 0.00 | 0.00 |
| GO:0008033 | tRNA processing | 0.00 | 0.00 |
| GO:0002768 | immune response-regulating cell surface receptor signaling pathway | 0.00 | 0.00 |
| GO:0031668 | cellular response to extracellular stimulus | 0.00 | 0.00 |
| GO:0034976 | response to endoplasmic reticulum stress | 0.00 | 0.00 |
| GO:0008154 | actin polymerization or depolymerization | 0.00 | 0.00 |
| GO:0050863 | regulation of T cell activation | 0.00 | 0.00 |
| GO:0002449 | lymphocyte mediated immunity | 0.00 | 0.00 |
| GO:0008360 | regulation of cell shape | 0.00 | 0.00 |
| GO:0046474 | glycerophospholipid biosynthetic process | 0.00 | 0.00 |
| GO:1903050 | regulation of proteolysis involved in cellular protein catabolic process | 0.00 | 0.00 |
| GO:0010563 | negative regulation of phosphorus metabolic process | 0.00 | 0.00 |
| GO:1903311 | regulation of mRNA metabolic process | 0.00 | 0.00 |
| GO:0031669 | cellular response to nutrient levels | 0.00 | 0.00 |
| GO:0045936 | negative regulation of phosphate metabolic process | 0.00 | 0.00 |
| GO:0009451 | RNA modification | 0.00 | 0.00 |
| GO:0006605 | protein targeting | 0.00 | 0.00 |
| GO:0007033 | vacuole organization | 0.00 | 0.00 |
| GO:0044409 | entry into host | 0.00 | 0.00 |
| GO:0002699 | positive regulation of immune effector process | 0.00 | 0.00 |
| GO:0098876 | vesicle-mediated transport to the plasma membrane | 0.00 | 0.00 |
| GO:2001242 | regulation of intrinsic apoptotic signaling pathway | 0.00 | 0.00 |
| GO:0042770 | signal transduction in response to DNA damage | 0.00 | 0.00 |
| GO:0008064 | regulation of actin polymerization or depolymerization | 0.00 | 0.00 |
| GO:0002440 | production of molecular mediator of immune response | 0.00 | 0.00 |
| GO:0002821 | positive regulation of adaptive immune response | 0.00 | 0.00 |
| GO:0034504 | protein localization to nucleus | 0.00 | 0.00 |
| GO:0022613 | ribonucleoprotein complex biogenesis | 0.00 | 0.00 |
| GO:0008654 | phospholipid biosynthetic process | 0.00 | 0.00 |
| GO:0002221 | pattern recognition receptor signaling pathway | 0.00 | 0.00 |
| GO:0010970 | transport along microtubule | 0.00 | 0.00 |
| GO:0001819 | positive regulation of cytokine production | 0.00 | 0.00 |
| GO:0006979 | response to oxidative stress | 0.00 | 0.00 |
| GO:0002819 | regulation of adaptive immune response | 0.00 | 0.00 |
| GO:0006661 | phosphatidylinositol biosynthetic process | 0.00 | 0.00 |
| GO:0060491 | regulation of cell projection assembly | 0.00 | 0.00 |
| GO:0016482 | cytosolic transport | 0.00 | 0.00 |
| GO:0032434 | regulation of proteasomal ubiquitin-dependent protein catabolic process | 0.00 | 0.00 |
| GO:0045732 | positive regulation of protein catabolic process | 0.00 | 0.00 |
| GO:0030832 | regulation of actin filament length | 0.00 | 0.00 |
| GO:0062197 | cellular response to chemical stress | 0.00 | 0.00 |
| GO:0016050 | vesicle organization | 0.00 | 0.00 |
| GO:0006753 | nucleoside phosphate metabolic process | 0.00 | 0.00 |
| GO:1901800 | positive regulation of proteasomal protein catabolic process | 0.00 | 0.00 |
| GO:2001022 | positive regulation of response to DNA damage stimulus | 0.00 | 0.00 |
| GO:0120032 | regulation of plasma membrane bounded cell projection assembly | 0.00 | 0.00 |
| GO:0000725 | recombinational repair | 0.00 | 0.00 |
| GO:0032271 | regulation of protein polymerization | 0.00 | 0.00 |
| GO:0016064 | immunoglobulin mediated immune response | 0.00 | 0.00 |
| GO:0019724 | B cell mediated immunity | 0.00 | 0.00 |
| GO:0150115 | cell-substrate junction organization | 0.00 | 0.00 |
| GO:0007051 | spindle organization | 0.00 | 0.00 |
| GO:0043123 | positive regulation of I-kappaB kinase/NF-kappaB signaling | 0.00 | 0.00 |
| GO:0002253 | activation of immune response | 0.00 | 0.00 |
| GO:0032386 | regulation of intracellular transport | 0.00 | 0.00 |
| GO:1902117 | positive regulation of organelle assembly | 0.00 | 0.00 |
| GO:0030865 | cortical cytoskeleton organization | 0.00 | 0.00 |
| GO:0051098 | regulation of binding | 0.00 | 0.00 |
| GO:0030041 | actin filament polymerization | 0.00 | 0.00 |
| GO:1905475 | regulation of protein localization to membrane | 0.00 | 0.00 |
| GO:1903706 | regulation of hemopoiesis | 0.00 | 0.00 |
| GO:0050851 | antigen receptor-mediated signaling pathway | 0.00 | 0.00 |
| GO:0000724 | double-strand break repair via homologous recombination | 0.00 | 0.00 |
| GO:0051056 | regulation of small GTPase mediated signal transduction | 0.00 | 0.00 |
| GO:0009117 | nucleotide metabolic process | 0.00 | 0.00 |
| GO:0046718 | viral entry into host cell | 0.00 | 0.00 |
| GO:0016049 | cell growth | 0.00 | 0.00 |
| GO:0002824 | positive regulation of adaptive immune response based on somatic recombination of immune receptors built from immunoglobulin superfamily domains | 0.00 | 0.00 |
| GO:0051495 | positive regulation of cytoskeleton organization | 0.00 | 0.00 |
| GO:0050684 | regulation of mRNA processing | 0.00 | 0.00 |
| GO:0006091 | generation of precursor metabolites and energy | 0.00 | 0.00 |
| GO:0097581 | lamellipodium organization | 0.00 | 0.00 |
| GO:0044403 | biological process involved in symbiotic interaction | 0.00 | 0.00 |
| GO:0016573 | histone acetylation | 0.00 | 0.00 |
| GO:0006401 | RNA catabolic process | 0.00 | 0.00 |
| GO:0002822 | regulation of adaptive immune response based on somatic recombination of immune receptors built from immunoglobulin superfamily domains | 0.00 | 0.00 |
| GO:0071214 | cellular response to abiotic stimulus | 0.00 | 0.00 |
| GO:0104004 | cellular response to environmental stimulus | 0.00 | 0.00 |
| GO:0002703 | regulation of leukocyte mediated immunity | 0.00 | 0.00 |
| GO:0097191 | extrinsic apoptotic signaling pathway | 0.00 | 0.00 |
| GO:0002377 | immunoglobulin production | 0.00 | 0.00 |
| GO:0034446 | substrate adhesion-dependent cell spreading | 0.00 | 0.00 |
| GO:0090066 | regulation of anatomical structure size | 0.00 | 0.00 |
| GO:0044772 | mitotic cell cycle phase transition | 0.00 | 0.00 |
| GO:0001558 | regulation of cell growth | 0.00 | 0.00 |
| GO:1901987 | regulation of cell cycle phase transition | 0.00 | 0.00 |
| GO:0009267 | cellular response to starvation | 0.00 | 0.00 |
| GO:0045017 | glycerolipid biosynthetic process | 0.00 | 0.00 |
| GO:0048872 | homeostasis of number of cells | 0.00 | 0.00 |
| GO:2000058 | regulation of ubiquitin-dependent protein catabolic process | 0.00 | 0.00 |
| GO:0050900 | leukocyte migration | 0.00 | 0.00 |
| GO:0002697 | regulation of immune effector process | 0.00 | 0.00 |
| GO:0007265 | Ras protein signal transduction | 0.00 | 0.00 |
| GO:0018394 | peptidyl-lysine acetylation | 0.00 | 0.00 |
| GO:0035304 | regulation of protein dephosphorylation | 0.00 | 0.00 |
| GO:0046488 | phosphatidylinositol metabolic process | 0.00 | 0.00 |
| GO:0061572 | actin filament bundle organization | 0.00 | 0.00 |
| GO:1903322 | positive regulation of protein modification by small protein conjugation or removal | 0.00 | 0.00 |
| GO:0006473 | protein acetylation | 0.00 | 0.00 |
| GO:0140053 | mitochondrial gene expression | 0.00 | 0.00 |
| GO:0031647 | regulation of protein stability | 0.00 | 0.00 |
| GO:0006282 | regulation of DNA repair | 0.00 | 0.00 |
| GO:0035967 | cellular response to topologically incorrect protein | 0.00 | 0.00 |
| GO:0043414 | macromolecule methylation | 0.00 | 0.00 |
| GO:0016241 | regulation of macroautophagy | 0.00 | 0.00 |
| GO:0051017 | actin filament bundle assembly | 0.00 | 0.00 |
| GO:0030833 | regulation of actin filament polymerization | 0.00 | 0.00 |
| GO:0009615 | response to virus | 0.00 | 0.00 |
| GO:1902905 | positive regulation of supramolecular fiber organization | 0.00 | 0.00 |
| GO:2000779 | regulation of double-strand break repair | 0.00 | 0.00 |
| GO:0031589 | cell-substrate adhesion | 0.00 | 0.00 |
| GO:0031334 | positive regulation of protein-containing complex assembly | 0.00 | 0.00 |
| GO:0031023 | microtubule organizing center organization | 0.00 | 0.00 |
| GO:1903131 | mononuclear cell differentiation | 0.00 | 0.00 |
| GO:0019693 | ribose phosphate metabolic process | 0.00 | 0.00 |
| GO:0043484 | regulation of RNA splicing | 0.00 | 0.00 |
| GO:0009150 | purine ribonucleotide metabolic process | 0.00 | 0.00 |
| GO:1903364 | positive regulation of cellular protein catabolic process | 0.00 | 0.00 |
| GO:0031098 | stress-activated protein kinase signaling cascade | 0.00 | 0.00 |
| GO:0006475 | internal protein amino acid acetylation | 0.00 | 0.00 |
| GO:0006650 | glycerophospholipid metabolic process | 0.00 | 0.00 |
| GO:1904951 | positive regulation of establishment of protein localization | 0.00 | 0.00 |
| GO:0071479 | cellular response to ionizing radiation | 0.00 | 0.00 |
| GO:0007346 | regulation of mitotic cell cycle | 0.00 | 0.00 |
| GO:0007044 | cell-substrate junction assembly | 0.00 | 0.00 |
| GO:0009259 | ribonucleotide metabolic process | 0.00 | 0.00 |
| GO:0090311 | regulation of protein deacetylation | 0.00 | 0.00 |
| GO:0051403 | stress-activated MAPK cascade | 0.00 | 0.00 |
| GO:0051099 | positive regulation of binding | 0.00 | 0.00 |
| GO:0006476 | protein deacetylation | 0.00 | 0.00 |
| GO:0010332 | response to gamma radiation | 0.00 | 0.00 |
| GO:0007173 | epidermal growth factor receptor signaling pathway | 0.00 | 0.00 |
| GO:1904377 | positive regulation of protein localization to cell periphery | 0.00 | 0.00 |
| GO:0009411 | response to UV | 0.00 | 0.00 |
| GO:0001933 | negative regulation of protein phosphorylation | 0.00 | 0.00 |
| GO:0031346 | positive regulation of cell projection organization | 0.00 | 0.00 |
| GO:0051222 | positive regulation of protein transport | 0.00 | 0.00 |
| GO:0031532 | actin cytoskeleton reorganization | 0.00 | 0.00 |
| GO:0050864 | regulation of B cell activation | 0.00 | 0.00 |
| GO:0007093 | mitotic cell cycle checkpoint signaling | 0.00 | 0.00 |
| GO:0030168 | platelet activation | 0.00 | 0.00 |
| GO:0035303 | regulation of dephosphorylation | 0.00 | 0.00 |
| GO:0030522 | intracellular receptor signaling pathway | 0.00 | 0.00 |
| GO:0000075 | cell cycle checkpoint signaling | 0.00 | 0.00 |
| GO:0018393 | internal peptidyl-lysine acetylation | 0.00 | 0.00 |
| GO:0006839 | mitochondrial transport | 0.00 | 0.00 |
| GO:0042060 | wound healing | 0.00 | 0.00 |
| GO:0045911 | positive regulation of DNA recombination | 0.00 | 0.00 |
| GO:0006892 | post-Golgi vesicle-mediated transport | 0.00 | 0.00 |
| GO:0045582 | positive regulation of T cell differentiation | 0.00 | 0.00 |
| GO:0002263 | cell activation involved in immune response | 0.00 | 0.00 |
| GO:0010821 | regulation of mitochondrion organization | 0.00 | 0.00 |
| GO:0000045 | autophagosome assembly | 0.00 | 0.00 |
| GO:1902107 | positive regulation of leukocyte differentiation | 0.00 | 0.00 |
| GO:1903708 | positive regulation of hemopoiesis | 0.00 | 0.00 |
| GO:0035601 | protein deacylation | 0.00 | 0.00 |
| GO:0048285 | organelle fission | 0.00 | 0.00 |
| GO:0016239 | positive regulation of macroautophagy | 0.00 | 0.00 |
| GO:1901796 | regulation of signal transduction by p53 class mediator | 0.00 | 0.00 |
| GO:1905477 | positive regulation of protein localization to membrane | 0.00 | 0.00 |
| GO:0032388 | positive regulation of intracellular transport | 0.00 | 0.00 |
| GO:0099111 | microtubule-based transport | 0.00 | 0.00 |
| GO:0120034 | positive regulation of plasma membrane bounded cell projection assembly | 0.00 | 0.00 |
| GO:0006261 | DNA-templated DNA replication | 0.00 | 0.00 |
| GO:0000377 | RNA splicing, via transesterification reactions with bulged adenosine as nucleophile | 0.00 | 0.00 |
| GO:0000398 | mRNA splicing, via spliceosome | 0.00 | 0.00 |
| GO:0030098 | lymphocyte differentiation | 0.00 | 0.00 |
| GO:0042326 | negative regulation of phosphorylation | 0.00 | 0.00 |
| GO:0033627 | cell adhesion mediated by integrin | 0.00 | 0.00 |
| GO:0034599 | cellular response to oxidative stress | 0.00 | 0.00 |
| GO:0006360 | transcription by RNA polymerase I | 0.00 | 0.00 |
| GO:0007098 | centrosome cycle | 0.00 | 0.00 |
| GO:0002831 | regulation of response to biotic stimulus | 0.00 | 0.00 |
| GO:0030330 | DNA damage response, signal transduction by p53 class mediator | 0.00 | 0.00 |
| GO:0002431 | Fc receptor mediated stimulatory signaling pathway | 0.00 | 0.00 |
| GO:0000956 | nuclear-transcribed mRNA catabolic process | 0.00 | 0.00 |
| GO:0031398 | positive regulation of protein ubiquitination | 0.00 | 0.00 |
| GO:1903008 | organelle disassembly | 0.00 | 0.00 |
| GO:0006790 | sulfur compound metabolic process | 0.00 | 0.00 |
| GO:0030217 | T cell differentiation | 0.00 | 0.00 |
| GO:0000375 | RNA splicing, via transesterification reactions | 0.00 | 0.00 |
| GO:0046854 | phosphatidylinositol phosphate biosynthetic process | 0.00 | 0.00 |
| GO:0033674 | positive regulation of kinase activity | 0.00 | 0.00 |
| GO:0044774 | mitotic DNA integrity checkpoint signaling | 0.00 | 0.00 |
| GO:1905037 | autophagosome organization | 0.00 | 0.00 |
| GO:0006479 | protein methylation | 0.00 | 0.00 |
| GO:0008213 | protein alkylation | 0.00 | 0.00 |
| GO:0002366 | leukocyte activation involved in immune response | 0.00 | 0.00 |
| GO:0051091 | positive regulation of DNA-binding transcription factor activity | 0.00 | 0.00 |
| GO:0018209 | peptidyl-serine modification | 0.00 | 0.00 |
| GO:0090150 | establishment of protein localization to membrane | 0.00 | 0.00 |
| GO:0006469 | negative regulation of protein kinase activity | 0.00 | 0.00 |
| GO:0031570 | DNA integrity checkpoint signaling | 0.00 | 0.00 |
| GO:0044773 | mitotic DNA damage checkpoint signaling | 0.00 | 0.00 |
| GO:0006402 | mRNA catabolic process | 0.00 | 0.00 |
| GO:0018105 | peptidyl-serine phosphorylation | 0.00 | 0.00 |
| GO:0051348 | negative regulation of transferase activity | 0.00 | 0.00 |
| GO:0002753 | cytoplasmic pattern recognition receptor signaling pathway | 0.00 | 0.00 |
| GO:1903078 | positive regulation of protein localization to plasma membrane | 0.00 | 0.00 |
| GO:0045621 | positive regulation of lymphocyte differentiation | 0.00 | 0.00 |
| GO:0042274 | ribosomal small subunit biogenesis | 0.00 | 0.00 |
| GO:0051223 | regulation of protein transport | 0.00 | 0.00 |
| GO:0090316 | positive regulation of intracellular protein transport | 0.00 | 0.00 |
| GO:0032456 | endocytic recycling | 0.00 | 0.00 |
| GO:0042113 | B cell activation | 0.00 | 0.00 |
| GO:0031929 | TOR signaling | 0.00 | 0.00 |
| GO:0030866 | cortical actin cytoskeleton organization | 0.00 | 0.00 |
| GO:0051650 | establishment of vesicle localization | 0.00 | 0.00 |
| GO:0002275 | myeloid cell activation involved in immune response | 0.00 | 0.00 |
| GO:0006303 | double-strand break repair via nonhomologous end joining | 0.00 | 0.00 |
| GO:0048041 | focal adhesion assembly | 0.00 | 0.00 |
| GO:0000819 | sister chromatid segregation | 0.00 | 0.00 |
| GO:0034440 | lipid oxidation | 0.00 | 0.00 |
| GO:0014020 | primary neural tube formation | 0.00 | 0.00 |
| GO:0000077 | DNA damage checkpoint signaling | 0.00 | 0.00 |
| GO:0045926 | negative regulation of growth | 0.00 | 0.00 |
| GO:0044282 | small molecule catabolic process | 0.00 | 0.00 |
| GO:0042594 | response to starvation | 0.00 | 0.00 |
| GO:0038127 | ERBB signaling pathway | 0.00 | 0.00 |
| GO:0051225 | spindle assembly | 0.00 | 0.00 |
| GO:0061099 | negative regulation of protein tyrosine kinase activity | 0.00 | 0.00 |
| GO:0046635 | positive regulation of alpha-beta T cell activation | 0.00 | 0.00 |
| GO:0098732 | macromolecule deacylation | 0.00 | 0.00 |
| GO:2001234 | negative regulation of apoptotic signaling pathway | 0.00 | 0.00 |
| GO:0072521 | purine-containing compound metabolic process | 0.00 | 0.00 |
| GO:0032418 | lysosome localization | 0.00 | 0.00 |
| GO:1990849 | vacuolar localization | 0.00 | 0.00 |
| GO:0035966 | response to topologically incorrect protein | 0.00 | 0.00 |
| GO:0042098 | T cell proliferation | 0.00 | 0.00 |
| GO:0032436 | positive regulation of proteasomal ubiquitin-dependent protein catabolic process | 0.00 | 0.00 |
| GO:1905897 | regulation of response to endoplasmic reticulum stress | 0.00 | 0.00 |
| GO:0051648 | vesicle localization | 0.00 | 0.00 |
| GO:0034620 | cellular response to unfolded protein | 0.00 | 0.00 |
| GO:0000070 | mitotic sister chromatid segregation | 0.00 | 0.00 |
| GO:0001841 | neural tube formation | 0.00 | 0.00 |
| GO:0006400 | tRNA modification | 0.00 | 0.00 |
| GO:0007029 | endoplasmic reticulum organization | 0.00 | 0.00 |
| GO:0032259 | methylation | 0.00 | 0.00 |
| GO:2001244 | positive regulation of intrinsic apoptotic signaling pathway | 0.00 | 0.00 |
| GO:0006513 | protein monoubiquitination | 0.00 | 0.00 |
| GO:0060996 | dendritic spine development | 0.00 | 0.00 |
| GO:0045637 | regulation of myeloid cell differentiation | 0.00 | 0.00 |
| GO:0007059 | chromosome segregation | 0.00 | 0.00 |
| GO:0030032 | lamellipodium assembly | 0.00 | 0.00 |
| GO:0019318 | hexose metabolic process | 0.00 | 0.00 |
| GO:0043244 | regulation of protein-containing complex disassembly | 0.00 | 0.00 |
| GO:0070585 | protein localization to mitochondrion | 0.00 | 0.00 |
| GO:0006163 | purine nucleotide metabolic process | 0.00 | 0.00 |
| GO:0031122 | cytoplasmic microtubule organization | 0.00 | 0.00 |
| GO:0044380 | protein localization to cytoskeleton | 0.00 | 0.00 |
| GO:0046631 | alpha-beta T cell activation | 0.00 | 0.00 |
| GO:0000018 | regulation of DNA recombination | 0.00 | 0.00 |
| GO:0032753 | positive regulation of interleukin-4 production | 0.00 | 0.00 |
| GO:0050871 | positive regulation of B cell activation | 0.00 | 0.00 |
| GO:1903052 | positive regulation of proteolysis involved in cellular protein catabolic process | 0.00 | 0.00 |
| GO:0031397 | negative regulation of protein ubiquitination | 0.00 | 0.00 |
| GO:0019080 | viral gene expression | 0.00 | 0.00 |
| GO:0031058 | positive regulation of histone modification | 0.00 | 0.00 |
| GO:0006644 | phospholipid metabolic process | 0.00 | 0.00 |
| GO:0005996 | monosaccharide metabolic process | 0.00 | 0.00 |
| GO:0019395 | fatty acid oxidation | 0.00 | 0.00 |
| GO:0007599 | hemostasis | 0.00 | 0.00 |
| GO:0070085 | glycosylation | 0.00 | 0.00 |
| GO:0010975 | regulation of neuron projection development | 0.00 | 0.00 |
| GO:0048732 | gland development | 0.00 | 0.00 |
| GO:0072698 | protein localization to microtubule cytoskeleton | 0.00 | 0.00 |
| GO:0043281 | regulation of cysteine-type endopeptidase activity involved in apoptotic process | 0.00 | 0.00 |
| GO:0006919 | activation of cysteine-type endopeptidase activity involved in apoptotic process | 0.00 | 0.00 |
| GO:0006913 | nucleocytoplasmic transport | 0.00 | 0.00 |
| GO:0051169 | nuclear transport | 0.00 | 0.00 |
| GO:0001843 | neural tube closure | 0.00 | 0.00 |
| GO:0070302 | regulation of stress-activated protein kinase signaling cascade | 0.00 | 0.00 |
| GO:0007596 | blood coagulation | 0.00 | 0.00 |
| GO:0032872 | regulation of stress-activated MAPK cascade | 0.00 | 0.00 |
| GO:0002262 | myeloid cell homeostasis | 0.00 | 0.00 |
| GO:0045786 | negative regulation of cell cycle | 0.00 | 0.00 |
| GO:0021915 | neural tube development | 0.00 | 0.00 |
| GO:0002274 | myeloid leukocyte activation | 0.00 | 0.00 |
| GO:0006310 | DNA recombination | 0.00 | 0.00 |
| GO:1902850 | microtubule cytoskeleton organization involved in mitosis | 0.00 | 0.00 |
| GO:1903321 | negative regulation of protein modification by small protein conjugation or removal | 0.00 | 0.00 |
| GO:0000959 | mitochondrial RNA metabolic process | 0.00 | 0.00 |
| GO:0050686 | negative regulation of mRNA processing | 0.00 | 0.00 |
| GO:0031529 | ruffle organization | 0.00 | 0.00 |
| GO:0043967 | histone H4 acetylation | 0.00 | 0.00 |
| GO:0016572 | histone phosphorylation | 0.00 | 0.00 |
| GO:2000785 | regulation of autophagosome assembly | 0.00 | 0.00 |
| GO:0009141 | nucleoside triphosphate metabolic process | 0.00 | 0.00 |
| GO:0050792 | regulation of viral process | 0.00 | 0.00 |
| GO:0032633 | interleukin-4 production | 0.00 | 0.00 |
| GO:0032673 | regulation of interleukin-4 production | 0.00 | 0.00 |
| GO:0072655 | establishment of protein localization to mitochondrion | 0.00 | 0.00 |
| GO:1905168 | positive regulation of double-strand break repair via homologous recombination | 0.00 | 0.00 |
| GO:0033673 | negative regulation of kinase activity | 0.00 | 0.00 |
| GO:0009991 | response to extracellular stimulus | 0.00 | 0.00 |
| GO:0007163 | establishment or maintenance of cell polarity | 0.00 | 0.00 |
| GO:0072527 | pyrimidine-containing compound metabolic process | 0.00 | 0.00 |
| GO:0018212 | peptidyl-tyrosine modification | 0.00 | 0.00 |
| GO:0019882 | antigen processing and presentation | 0.00 | 0.00 |
| GO:0032543 | mitochondrial translation | 0.00 | 0.00 |
| GO:0007032 | endosome organization | 0.00 | 0.00 |
| GO:0031667 | response to nutrient levels | 0.00 | 0.00 |
| GO:0007034 | vacuolar transport | 0.00 | 0.00 |
| GO:0033157 | regulation of intracellular protein transport | 0.00 | 0.00 |
| GO:0018108 | peptidyl-tyrosine phosphorylation | 0.00 | 0.00 |
| GO:0061008 | hepaticobiliary system development | 0.00 | 0.00 |
| GO:1901990 | regulation of mitotic cell cycle phase transition | 0.00 | 0.00 |
| GO:0006275 | regulation of DNA replication | 0.00 | 0.00 |
| GO:0007254 | JNK cascade | 0.00 | 0.00 |
| GO:0060606 | tube closure | 0.00 | 0.00 |
| GO:0001522 | pseudouridine synthesis | 0.00 | 0.00 |
| GO:0071786 | endoplasmic reticulum tubular network organization | 0.00 | 0.00 |
| GO:0001914 | regulation of T cell mediated cytotoxicity | 0.00 | 0.00 |
| GO:0007492 | endoderm development | 0.00 | 0.00 |
| GO:1900024 | regulation of substrate adhesion-dependent cell spreading | 0.00 | 0.00 |
| GO:0044088 | regulation of vacuole organization | 0.00 | 0.00 |
| GO:0050817 | coagulation | 0.00 | 0.00 |
| GO:0051607 | defense response to virus | 0.00 | 0.00 |
| GO:0010165 | response to X-ray | 0.00 | 0.00 |
| GO:0007020 | microtubule nucleation | 0.00 | 0.00 |
| GO:0150116 | regulation of cell-substrate junction organization | 0.00 | 0.00 |
| GO:2000756 | regulation of peptidyl-lysine acetylation | 0.00 | 0.00 |
| GO:0046434 | organophosphate catabolic process | 0.00 | 0.00 |
| GO:0032006 | regulation of TOR signaling | 0.00 | 0.00 |
| GO:0051090 | regulation of DNA-binding transcription factor activity | 0.00 | 0.00 |
| GO:0000281 | mitotic cytokinesis | 0.00 | 0.00 |
| GO:0000910 | cytokinesis | 0.00 | 0.00 |
| GO:0007041 | lysosomal transport | 0.00 | 0.00 |
| GO:0007052 | mitotic spindle organization | 0.00 | 0.00 |
| GO:0051604 | protein maturation | 0.00 | 0.00 |
| GO:0006486 | protein glycosylation | 0.00 | 0.00 |
| GO:0043413 | macromolecule glycosylation | 0.00 | 0.00 |
| GO:0071902 | positive regulation of protein serine/threonine kinase activity | 0.00 | 0.00 |
| GO:0051494 | negative regulation of cytoskeleton organization | 0.00 | 0.00 |
| GO:0009314 | response to radiation | 0.00 | 0.00 |
| GO:0032508 | DNA duplex unwinding | 0.00 | 0.00 |
| GO:0034502 | protein localization to chromosome | 0.00 | 0.00 |
| GO:2000781 | positive regulation of double-strand break repair | 0.00 | 0.00 |
| GO:0031063 | regulation of histone deacetylation | 0.00 | 0.00 |
| GO:0031032 | actomyosin structure organization | 0.00 | 0.00 |
| GO:0050670 | regulation of lymphocyte proliferation | 0.00 | 0.00 |
| GO:0140546 | defense response to symbiont | 0.00 | 0.00 |
| GO:0009062 | fatty acid catabolic process | 0.00 | 0.00 |
| GO:0001913 | T cell mediated cytotoxicity | 0.00 | 0.00 |
| GO:0046638 | positive regulation of alpha-beta T cell differentiation | 0.00 | 0.00 |
| GO:0034063 | stress granule assembly | 0.00 | 0.00 |
| GO:0097205 | renal filtration | 0.00 | 0.00 |
| GO:0002705 | positive regulation of leukocyte mediated immunity | 0.00 | 0.00 |
| GO:0046632 | alpha-beta T cell differentiation | 0.00 | 0.00 |
| GO:0031109 | microtubule polymerization or depolymerization | 0.00 | 0.00 |
| GO:0002702 | positive regulation of production of molecular mediator of immune response | 0.00 | 0.00 |
| GO:0007160 | cell-matrix adhesion | 0.00 | 0.00 |
| GO:0010569 | regulation of double-strand break repair via homologous recombination | 0.00 | 0.00 |
| GO:0071260 | cellular response to mechanical stimulus | 0.00 | 0.00 |
| GO:0045860 | positive regulation of protein kinase activity | 0.00 | 0.00 |
| GO:0046486 | glycerolipid metabolic process | 0.00 | 0.00 |
| GO:0010948 | negative regulation of cell cycle process | 0.00 | 0.00 |
| GO:2001252 | positive regulation of chromosome organization | 0.00 | 0.00 |
| GO:0044839 | cell cycle G2/M phase transition | 0.00 | 0.00 |
| GO:0035307 | positive regulation of protein dephosphorylation | 0.00 | 0.00 |
| GO:0002381 | immunoglobulin production involved in immunoglobulin-mediated immune response | 0.00 | 0.00 |
| GO:0002181 | cytoplasmic translation | 0.00 | 0.00 |
| GO:0019221 | cytokine-mediated signaling pathway | 0.00 | 0.00 |
| GO:0032623 | interleukin-2 production | 0.00 | 0.00 |
| GO:0032663 | regulation of interleukin-2 production | 0.00 | 0.00 |
| GO:1905515 | non-motile cilium assembly | 0.00 | 0.00 |
| GO:0016575 | histone deacetylation | 0.00 | 0.00 |
| GO:0000280 | nuclear division | 0.00 | 0.00 |
| GO:0006635 | fatty acid beta-oxidation | 0.00 | 0.00 |
| GO:0030968 | endoplasmic reticulum unfolded protein response | 0.00 | 0.00 |
| GO:0001889 | liver development | 0.00 | 0.00 |
| GO:2001235 | positive regulation of apoptotic signaling pathway | 0.00 | 0.00 |
| GO:0001909 | leukocyte mediated cytotoxicity | 0.00 | 0.00 |
| GO:0050853 | B cell receptor signaling pathway | 0.00 | 0.00 |
| GO:0030308 | negative regulation of cell growth | 0.00 | 0.00 |
| GO:0045088 | regulation of innate immune response | 0.00 | 0.00 |
| GO:0010810 | regulation of cell-substrate adhesion | 0.00 | 0.00 |
| GO:1901292 | nucleoside phosphate catabolic process | 0.00 | 0.00 |
| GO:1901983 | regulation of protein acetylation | 0.00 | 0.00 |
| GO:0071897 | DNA biosynthetic process | 0.00 | 0.00 |
| GO:0072384 | organelle transport along microtubule | 0.00 | 0.00 |
| GO:1902105 | regulation of leukocyte differentiation | 0.00 | 0.00 |
| GO:0046390 | ribose phosphate biosynthetic process | 0.00 | 0.00 |
| GO:0002711 | positive regulation of T cell mediated immunity | 0.00 | 0.00 |
| GO:0002886 | regulation of myeloid leukocyte mediated immunity | 0.00 | 0.00 |
| GO:0035065 | regulation of histone acetylation | 0.00 | 0.00 |
| GO:0002433 | immune response-regulating cell surface receptor signaling pathway involved in phagocytosis | 0.00 | 0.00 |
| GO:0003094 | glomerular filtration | 0.00 | 0.00 |
| GO:0019883 | antigen processing and presentation of endogenous antigen | 0.00 | 0.00 |
| GO:0033598 | mammary gland epithelial cell proliferation | 0.00 | 0.00 |
| GO:0038096 | Fc-gamma receptor signaling pathway involved in phagocytosis | 0.00 | 0.00 |
| GO:0043966 | histone H3 acetylation | 0.00 | 0.00 |
| GO:0045739 | positive regulation of DNA repair | 0.00 | 0.00 |
| GO:0006338 | chromatin remodeling | 0.00 | 0.00 |
| GO:1905508 | protein localization to microtubule organizing center | 0.00 | 0.00 |
| GO:0046651 | lymphocyte proliferation | 0.00 | 0.00 |
| GO:0061640 | cytoskeleton-dependent cytokinesis | 0.00 | 0.00 |
| GO:0050852 | T cell receptor signaling pathway | 0.00 | 0.00 |
| GO:0038093 | Fc receptor signaling pathway | 0.00 | 0.00 |
| GO:0007176 | regulation of epidermal growth factor-activated receptor activity | 0.00 | 0.00 |
| GO:0010458 | exit from mitosis | 0.00 | 0.00 |
| GO:0038094 | Fc-gamma receptor signaling pathway | 0.00 | 0.00 |
| GO:0001503 | ossification | 0.00 | 0.00 |
| GO:0001838 | embryonic epithelial tube formation | 0.00 | 0.00 |
| GO:1903313 | positive regulation of mRNA metabolic process | 0.00 | 0.00 |
| GO:0048024 | regulation of mRNA splicing, via spliceosome | 0.00 | 0.00 |
| GO:0036503 | ERAD pathway | 0.00 | 0.00 |
| GO:0043393 | regulation of protein binding | 0.00 | 0.00 |
| GO:0009123 | nucleoside monophosphate metabolic process | 0.00 | 0.00 |
| GO:0006898 | receptor-mediated endocytosis | 0.00 | 0.00 |
| GO:2001224 | positive regulation of neuron migration | 0.00 | 0.00 |
| GO:0007369 | gastrulation | 0.00 | 0.00 |
| GO:0010324 | membrane invagination | 0.00 | 0.00 |
| GO:2000116 | regulation of cysteine-type endopeptidase activity | 0.00 | 0.00 |
| GO:0046785 | microtubule polymerization | 0.00 | 0.00 |
| GO:0002687 | positive regulation of leukocyte migration | 0.00 | 0.00 |
| GO:0045727 | positive regulation of translation | 0.00 | 0.00 |
| GO:0046328 | regulation of JNK cascade | 0.00 | 0.00 |
| GO:0009260 | ribonucleotide biosynthetic process | 0.00 | 0.00 |
| GO:0009144 | purine nucleoside triphosphate metabolic process | 0.00 | 0.00 |
| GO:1901985 | positive regulation of protein acetylation | 0.00 | 0.00 |
| GO:0006887 | exocytosis | 0.00 | 0.00 |
| GO:0016571 | histone methylation | 0.00 | 0.00 |
| GO:0006497 | protein lipidation | 0.00 | 0.00 |
| GO:0010769 | regulation of cell morphogenesis involved in differentiation | 0.00 | 0.00 |
| GO:0032392 | DNA geometric change | 0.00 | 0.00 |
| GO:0002224 | toll-like receptor signaling pathway | 0.00 | 0.00 |
| GO:0060997 | dendritic spine morphogenesis | 0.00 | 0.00 |
| GO:0045639 | positive regulation of myeloid cell differentiation | 0.00 | 0.00 |
| GO:0046634 | regulation of alpha-beta T cell activation | 0.00 | 0.00 |
| GO:2000060 | positive regulation of ubiquitin-dependent protein catabolic process | 0.00 | 0.00 |
| GO:2000758 | positive regulation of peptidyl-lysine acetylation | 0.00 | 0.00 |
| GO:0015980 | energy derivation by oxidation of organic compounds | 0.00 | 0.00 |
| GO:0035306 | positive regulation of dephosphorylation | 0.00 | 0.00 |
| GO:0016055 | Wnt signaling pathway | 0.00 | 0.00 |
| GO:0097178 | ruffle assembly | 0.00 | 0.00 |
| GO:1902116 | negative regulation of organelle assembly | 0.00 | 0.00 |
| GO:0033119 | negative regulation of RNA splicing | 0.00 | 0.00 |
| GO:0006356 | regulation of transcription by RNA polymerase I | 0.00 | 0.00 |
| GO:0000086 | G2/M transition of mitotic cell cycle | 0.00 | 0.00 |
| GO:0030595 | leukocyte chemotaxis | 0.00 | 0.00 |
| GO:0032204 | regulation of telomere maintenance | 0.00 | 0.00 |
| GO:2001032 | regulation of double-strand break repair via nonhomologous end joining | 0.00 | 0.00 |
| GO:0032944 | regulation of mononuclear cell proliferation | 0.00 | 0.00 |
| GO:0032092 | positive regulation of protein binding | 0.00 | 0.00 |
| GO:0030433 | ubiquitin-dependent ERAD pathway | 0.00 | 0.00 |
| GO:0050821 | protein stabilization | 0.00 | 0.00 |
| GO:0043280 | positive regulation of cysteine-type endopeptidase activity involved in apoptotic process | 0.00 | 0.00 |
| GO:0010811 | positive regulation of cell-substrate adhesion | 0.00 | 0.00 |
| GO:0008630 | intrinsic apoptotic signaling pathway in response to DNA damage | 0.00 | 0.00 |
| GO:0042158 | lipoprotein biosynthetic process | 0.00 | 0.00 |
| GO:0007229 | integrin-mediated signaling pathway | 0.00 | 0.00 |
| GO:0030038 | contractile actin filament bundle assembly | 0.00 | 0.00 |
| GO:0043149 | stress fiber assembly | 0.00 | 0.00 |
| GO:0030111 | regulation of Wnt signaling pathway | 0.00 | 0.00 |
| GO:0016578 | histone deubiquitination | 0.00 | 0.00 |
| GO:0019884 | antigen processing and presentation of exogenous antigen | 0.00 | 0.00 |
| GO:0032008 | positive regulation of TOR signaling | 0.00 | 0.00 |
| GO:1901293 | nucleoside phosphate biosynthetic process | 0.00 | 0.00 |
| GO:0017038 | protein import | 0.00 | 0.00 |
| GO:0006986 | response to unfolded protein | 0.00 | 0.00 |
| GO:0019827 | stem cell population maintenance | 0.00 | 0.00 |
| GO:1903900 | regulation of viral life cycle | 0.00 | 0.00 |
| GO:0042058 | regulation of epidermal growth factor receptor signaling pathway | 0.00 | 0.00 |
| GO:0034101 | erythrocyte homeostasis | 0.00 | 0.00 |
| GO:0061912 | selective autophagy | 0.00 | 0.00 |
| GO:0009100 | glycoprotein metabolic process | 0.00 | 0.00 |
| GO:0006893 | Golgi to plasma membrane transport | 0.00 | 0.00 |
| GO:0002504 | antigen processing and presentation of peptide or polysaccharide antigen via MHC class II | 0.00 | 0.00 |
| GO:0042149 | cellular response to glucose starvation | 0.00 | 0.00 |
| GO:0045444 | fat cell differentiation | 0.00 | 0.00 |
| GO:0030218 | erythrocyte differentiation | 0.00 | 0.00 |
| GO:0009152 | purine ribonucleotide biosynthetic process | 0.00 | 0.00 |
| GO:0009205 | purine ribonucleoside triphosphate metabolic process | 0.00 | 0.00 |
| GO:0043242 | negative regulation of protein-containing complex disassembly | 0.00 | 0.00 |
| GO:0043367 | CD4-positive, alpha-beta T cell differentiation | 0.00 | 0.00 |
| GO:0051261 | protein depolymerization | 0.00 | 0.00 |
| GO:0034329 | cell junction assembly | 0.00 | 0.00 |
| GO:0048002 | antigen processing and presentation of peptide antigen | 0.00 | 0.00 |
| GO:0051893 | regulation of focal adhesion assembly | 0.00 | 0.00 |
| GO:0070059 | intrinsic apoptotic signaling pathway in response to endoplasmic reticulum stress | 0.00 | 0.00 |
| GO:0090109 | regulation of cell-substrate junction assembly | 0.00 | 0.00 |
| GO:0034250 | positive regulation of cellular amide metabolic process | 0.00 | 0.00 |
| GO:0000422 | autophagy of mitochondrion | 0.00 | 0.00 |
| GO:0002709 | regulation of T cell mediated immunity | 0.00 | 0.00 |
| GO:0061726 | mitochondrion disassembly | 0.00 | 0.00 |
| GO:0002456 | T cell mediated immunity | 0.00 | 0.00 |
| GO:0071887 | leukocyte apoptotic process | 0.00 | 0.00 |
| GO:0061014 | positive regulation of mRNA catabolic process | 0.00 | 0.00 |
| GO:0062207 | regulation of pattern recognition receptor signaling pathway | 0.00 | 0.00 |
| GO:0032755 | positive regulation of interleukin-6 production | 0.00 | 0.00 |
| GO:0071168 | protein localization to chromatin | 0.00 | 0.00 |
| GO:0071539 | protein localization to centrosome | 0.00 | 0.00 |
| GO:0045333 | cellular respiration | 0.00 | 0.00 |
| GO:0198738 | cell-cell signaling by wnt | 0.00 | 0.00 |
| GO:0033059 | cellular pigmentation | 0.00 | 0.00 |
| GO:0043620 | regulation of DNA-templated transcription in response to stress | 0.00 | 0.00 |
| GO:0040029 | regulation of gene expression, epigenetic | 0.00 | 0.00 |
| GO:0002706 | regulation of lymphocyte mediated immunity | 0.00 | 0.00 |
| GO:0016556 | mRNA modification | 0.00 | 0.00 |
| GO:0032801 | receptor catabolic process | 0.00 | 0.00 |
| GO:1901798 | positive regulation of signal transduction by p53 class mediator | 0.00 | 0.00 |
| GO:0016445 | somatic diversification of immunoglobulins | 0.00 | 0.00 |
| GO:0070527 | platelet aggregation | 0.00 | 0.00 |
| GO:0060326 | cell chemotaxis | 0.00 | 0.00 |
| GO:1900180 | regulation of protein localization to nucleus | 0.00 | 0.00 |
| GO:0051235 | maintenance of location | 0.00 | 0.00 |
| GO:0035987 | endodermal cell differentiation | 0.00 | 0.00 |
| GO:0098534 | centriole assembly | 0.00 | 0.00 |
| GO:0009166 | nucleotide catabolic process | 0.00 | 0.00 |
| GO:1901880 | negative regulation of protein depolymerization | 0.00 | 0.00 |
| GO:0035148 | tube formation | 0.00 | 0.00 |
| GO:0070507 | regulation of microtubule cytoskeleton organization | 0.00 | 0.00 |
| GO:0009165 | nucleotide biosynthetic process | 0.00 | 0.00 |
| GO:0043405 | regulation of MAP kinase activity | 0.00 | 0.00 |
| GO:0001704 | formation of primary germ layer | 0.00 | 0.00 |
| GO:0002708 | positive regulation of lymphocyte mediated immunity | 0.00 | 0.00 |
| GO:0043666 | regulation of phosphoprotein phosphatase activity | 0.00 | 0.00 |
| GO:0018022 | peptidyl-lysine methylation | 0.00 | 0.00 |
| GO:0007088 | regulation of mitotic nuclear division | 0.00 | 0.00 |
| GO:0010921 | regulation of phosphatase activity | 0.00 | 0.00 |
| GO:0022406 | membrane docking | 0.00 | 0.00 |
| GO:1990928 | response to amino acid starvation | 0.00 | 0.00 |
| GO:0042157 | lipoprotein metabolic process | 0.00 | 0.00 |
| GO:0046330 | positive regulation of JNK cascade | 0.00 | 0.00 |
| GO:0099024 | plasma membrane invagination | 0.00 | 0.00 |
| GO:0031663 | lipopolysaccharide-mediated signaling pathway | 0.00 | 0.00 |
| GO:0007095 | mitotic G2 DNA damage checkpoint signaling | 0.00 | 0.00 |
| GO:0031330 | negative regulation of cellular catabolic process | 0.00 | 0.00 |
| GO:0043467 | regulation of generation of precursor metabolites and energy | 0.00 | 0.00 |
| GO:0032943 | mononuclear cell proliferation | 0.00 | 0.00 |
| GO:0006403 | RNA localization | 0.00 | 0.00 |
| GO:2000786 | positive regulation of autophagosome assembly | 0.00 | 0.00 |
| GO:0006911 | phagocytosis, engulfment | 0.00 | 0.00 |
| GO:0006890 | retrograde vesicle-mediated transport, Golgi to endoplasmic reticulum | 0.00 | 0.00 |
| GO:0015931 | nucleobase-containing compound transport | 0.00 | 0.00 |
| GO:0002720 | positive regulation of cytokine production involved in immune response | 0.00 | 0.00 |
| GO:0031333 | negative regulation of protein-containing complex assembly | 0.00 | 0.00 |
| GO:0006006 | glucose metabolic process | 0.00 | 0.00 |
| GO:0001706 | endoderm formation | 0.00 | 0.00 |
| GO:0072583 | clathrin-dependent endocytosis | 0.00 | 0.00 |
| GO:0098927 | vesicle-mediated transport between endosomal compartments | 0.00 | 0.00 |
| GO:0061013 | regulation of mRNA catabolic process | 0.00 | 0.00 |
| GO:0070972 | protein localization to endoplasmic reticulum | 0.00 | 0.00 |
| GO:1903363 | negative regulation of cellular protein catabolic process | 0.00 | 0.00 |
| GO:0006622 | protein targeting to lysosome | 0.00 | 0.00 |
| GO:0072175 | epithelial tube formation | 0.00 | 0.00 |
| GO:0002444 | myeloid leukocyte mediated immunity | 0.00 | 0.00 |
| GO:0046822 | regulation of nucleocytoplasmic transport | 0.00 | 0.00 |
| GO:0043487 | regulation of RNA stability | 0.00 | 0.00 |
| GO:1904029 | regulation of cyclin-dependent protein kinase activity | 0.00 | 0.00 |
| GO:0000079 | regulation of cyclin-dependent protein serine/threonine kinase activity | 0.00 | 0.00 |
| GO:0001776 | leukocyte homeostasis | 0.00 | 0.00 |
| GO:0051492 | regulation of stress fiber assembly | 0.00 | 0.00 |
| GO:0050732 | negative regulation of peptidyl-tyrosine phosphorylation | 0.00 | 0.00 |
| GO:0002700 | regulation of production of molecular mediator of immune response | 0.00 | 0.00 |
| GO:0009132 | nucleoside diphosphate metabolic process | 0.00 | 0.00 |
| GO:0016331 | morphogenesis of embryonic epithelium | 0.00 | 0.00 |
| GO:0060562 | epithelial tube morphogenesis | 0.00 | 0.00 |
| GO:0010952 | positive regulation of peptidase activity | 0.00 | 0.00 |
| GO:0034968 | histone lysine methylation | 0.00 | 0.00 |
| GO:0044818 | mitotic G2/M transition checkpoint | 0.00 | 0.00 |
| GO:1901988 | negative regulation of cell cycle phase transition | 0.00 | 0.00 |
| GO:0090305 | nucleic acid phosphodiester bond hydrolysis | 0.00 | 0.00 |
| GO:0048284 | organelle fusion | 0.00 | 0.00 |
| GO:0050671 | positive regulation of lymphocyte proliferation | 0.00 | 0.00 |
| GO:0098727 | maintenance of cell number | 0.00 | 0.00 |
| GO:0002495 | antigen processing and presentation of peptide antigen via MHC class II | 0.00 | 0.00 |
| GO:0032743 | positive regulation of interleukin-2 production | 0.00 | 0.00 |
| GO:0035308 | negative regulation of protein dephosphorylation | 0.00 | 0.00 |
| GO:0039528 | cytoplasmic pattern recognition receptor signaling pathway in response to virus | 0.00 | 0.00 |
| GO:0051973 | positive regulation of telomerase activity | 0.00 | 0.00 |
| GO:0002714 | positive regulation of B cell mediated immunity | 0.00 | 0.00 |
| GO:0002891 | positive regulation of immunoglobulin mediated immune response | 0.00 | 0.00 |
| GO:0009299 | mRNA transcription | 0.00 | 0.00 |
| GO:0072595 | maintenance of protein localization in organelle | 0.00 | 0.00 |
| GO:1901224 | positive regulation of NIK/NF-kappaB signaling | 0.00 | 0.00 |
| GO:0007266 | Rho protein signal transduction | 0.00 | 0.00 |
| GO:0031503 | protein-containing complex localization | 0.00 | 0.00 |
| GO:0006109 | regulation of carbohydrate metabolic process | 0.00 | 0.00 |
| GO:1902904 | negative regulation of supramolecular fiber organization | 0.00 | 0.00 |
| GO:0043401 | steroid hormone mediated signaling pathway | 0.00 | 0.01 |
| GO:0032231 | regulation of actin filament bundle assembly | 0.00 | 0.01 |
| GO:2000573 | positive regulation of DNA biosynthetic process | 0.00 | 0.01 |
| GO:0043087 | regulation of GTPase activity | 0.00 | 0.01 |
| GO:0071103 | DNA conformation change | 0.00 | 0.01 |
| GO:0032874 | positive regulation of stress-activated MAPK cascade | 0.00 | 0.01 |
| GO:0009101 | glycoprotein biosynthetic process | 0.00 | 0.01 |
| GO:0009895 | negative regulation of catabolic process | 0.00 | 0.01 |
| GO:0031341 | regulation of cell killing | 0.00 | 0.01 |
| GO:1901184 | regulation of ERBB signaling pathway | 0.00 | 0.01 |
| GO:0050779 | RNA destabilization | 0.00 | 0.01 |
| GO:0042147 | retrograde transport, endosome to Golgi | 0.00 | 0.01 |
| GO:0051170 | import into nucleus | 0.00 | 0.01 |
| GO:0008625 | extrinsic apoptotic signaling pathway via death domain receptors | 0.00 | 0.01 |
| GO:0009142 | nucleoside triphosphate biosynthetic process | 0.00 | 0.01 |
| GO:1901879 | regulation of protein depolymerization | 0.00 | 0.01 |
| GO:0031345 | negative regulation of cell projection organization | 0.00 | 0.01 |
| GO:0051028 | mRNA transport | 0.00 | 0.01 |
| GO:0001916 | positive regulation of T cell mediated cytotoxicity | 0.00 | 0.01 |
| GO:0071480 | cellular response to gamma radiation | 0.00 | 0.01 |
| GO:0071674 | mononuclear cell migration | 0.00 | 0.01 |
| GO:0042129 | regulation of T cell proliferation | 0.00 | 0.01 |
| GO:0007030 | Golgi organization | 0.00 | 0.01 |
| GO:0015693 | magnesium ion transport | 0.00 | 0.01 |
| GO:0002244 | hematopoietic progenitor cell differentiation | 0.00 | 0.01 |
| GO:2001056 | positive regulation of cysteine-type endopeptidase activity | 0.00 | 0.01 |
| GO:0098781 | ncRNA transcription | 0.00 | 0.01 |
| GO:0007006 | mitochondrial membrane organization | 0.00 | 0.01 |
| GO:0000381 | regulation of alternative mRNA splicing, via spliceosome | 0.00 | 0.01 |
| GO:0016447 | somatic recombination of immunoglobulin gene segments | 0.00 | 0.01 |
| GO:0030071 | regulation of mitotic metaphase/anaphase transition | 0.00 | 0.01 |
| GO:0035331 | negative regulation of hippo signaling | 0.00 | 0.01 |
| GO:0070424 | regulation of nucleotide-binding oligomerization domain containing signaling pathway | 0.00 | 0.01 |
| GO:0035872 | nucleotide-binding domain, leucine rich repeat containing receptor signaling pathway | 0.00 | 0.01 |
| GO:0045724 | positive regulation of cilium assembly | 0.00 | 0.01 |
| GO:0051016 | barbed-end actin filament capping | 0.00 | 0.01 |
| GO:0034198 | cellular response to amino acid starvation | 0.00 | 0.01 |
| GO:0043618 | regulation of transcription from RNA polymerase II promoter in response to stress | 0.00 | 0.01 |
| GO:0045646 | regulation of erythrocyte differentiation | 0.00 | 0.01 |
| GO:0070849 | response to epidermal growth factor | 0.00 | 0.01 |
| GO:0008361 | regulation of cell size | 0.00 | 0.01 |
| GO:0070304 | positive regulation of stress-activated protein kinase signaling cascade | 0.00 | 0.01 |
| GO:0031124 | mRNA 3'-end processing | 0.00 | 0.01 |
| GO:0032615 | interleukin-12 production | 0.00 | 0.01 |
| GO:0032655 | regulation of interleukin-12 production | 0.00 | 0.01 |
| GO:1902099 | regulation of metaphase/anaphase transition of cell cycle | 0.00 | 0.01 |
| GO:0031065 | positive regulation of histone deacetylation | 0.00 | 0.01 |
| GO:0060828 | regulation of canonical Wnt signaling pathway | 0.00 | 0.01 |
| GO:0030837 | negative regulation of actin filament polymerization | 0.00 | 0.01 |
| GO:0046847 | filopodium assembly | 0.00 | 0.01 |
| GO:0016358 | dendrite development | 0.00 | 0.01 |
| GO:2001243 | negative regulation of intrinsic apoptotic signaling pathway | 0.00 | 0.01 |
| GO:0071478 | cellular response to radiation | 0.00 | 0.01 |
| GO:0071559 | response to transforming growth factor beta | 0.00 | 0.01 |
| GO:0007004 | telomere maintenance via telomerase | 0.00 | 0.01 |
| GO:0052372 | modulation by symbiont of entry into host | 0.00 | 0.01 |
| GO:1905818 | regulation of chromosome separation | 0.00 | 0.01 |
| GO:0032273 | positive regulation of protein polymerization | 0.00 | 0.01 |
| GO:0051865 | protein autoubiquitination | 0.00 | 0.01 |
| GO:0140056 | organelle localization by membrane tethering | 0.00 | 0.01 |
| GO:0000184 | nuclear-transcribed mRNA catabolic process, nonsense-mediated decay | 0.00 | 0.01 |
| GO:0043001 | Golgi to plasma membrane protein transport | 0.00 | 0.01 |
| GO:0045740 | positive regulation of DNA replication | 0.00 | 0.01 |
| GO:0061157 | mRNA destabilization | 0.00 | 0.01 |
| GO:0032272 | negative regulation of protein polymerization | 0.00 | 0.01 |
| GO:0051168 | nuclear export | 0.00 | 0.01 |
| GO:0048813 | dendrite morphogenesis | 0.00 | 0.01 |
| GO:2000278 | regulation of DNA biosynthetic process | 0.00 | 0.01 |
| GO:0000302 | response to reactive oxygen species | 0.00 | 0.01 |
| GO:1902743 | regulation of lamellipodium organization | 0.00 | 0.01 |
| GO:2001236 | regulation of extrinsic apoptotic signaling pathway | 0.00 | 0.01 |
| GO:0032886 | regulation of microtubule-based process | 0.00 | 0.01 |
| GO:0007584 | response to nutrient | 0.00 | 0.01 |
| GO:0001836 | release of cytochrome c from mitochondria | 0.00 | 0.01 |
| GO:0043903 | regulation of biological process involved in symbiotic interaction | 0.00 | 0.01 |
| GO:0042554 | superoxide anion generation | 0.00 | 0.01 |
| GO:0071364 | cellular response to epidermal growth factor stimulus | 0.00 | 0.01 |
| GO:0050878 | regulation of body fluid levels | 0.00 | 0.01 |
| GO:0046034 | ATP metabolic process | 0.00 | 0.01 |
| GO:2000377 | regulation of reactive oxygen species metabolic process | 0.00 | 0.01 |
| GO:0008637 | apoptotic mitochondrial changes | 0.00 | 0.01 |
| GO:1903557 | positive regulation of tumor necrosis factor superfamily cytokine production | 0.00 | 0.01 |
| GO:0030042 | actin filament depolymerization | 0.00 | 0.01 |
| GO:0030258 | lipid modification | 0.00 | 0.01 |
| GO:1903203 | regulation of oxidative stress-induced neuron death | 0.00 | 0.01 |
| GO:0045216 | cell-cell junction organization | 0.00 | 0.01 |
| GO:0006626 | protein targeting to mitochondrion | 0.00 | 0.01 |
| GO:0002712 | regulation of B cell mediated immunity | 0.00 | 0.01 |
| GO:0002889 | regulation of immunoglobulin mediated immune response | 0.00 | 0.01 |
| GO:0007091 | metaphase/anaphase transition of mitotic cell cycle | 0.00 | 0.01 |
| GO:0022617 | extracellular matrix disassembly | 0.00 | 0.01 |
| GO:0043516 | regulation of DNA damage response, signal transduction by p53 class mediator | 0.00 | 0.01 |
| GO:1902745 | positive regulation of lamellipodium organization | 0.00 | 0.01 |
| GO:0042177 | negative regulation of protein catabolic process | 0.00 | 0.01 |
| GO:0032602 | chemokine production | 0.00 | 0.01 |
| GO:0044784 | metaphase/anaphase transition of cell cycle | 0.00 | 0.01 |
| GO:0051310 | metaphase plate congression | 0.00 | 0.01 |
| GO:1902017 | regulation of cilium assembly | 0.00 | 0.01 |
| GO:0002455 | humoral immune response mediated by circulating immunoglobulin | 0.00 | 0.01 |
| GO:0051306 | mitotic sister chromatid separation | 0.00 | 0.01 |
| GO:0061180 | mammary gland epithelium development | 0.00 | 0.01 |
| GO:0009199 | ribonucleoside triphosphate metabolic process | 0.00 | 0.01 |
| GO:0051881 | regulation of mitochondrial membrane potential | 0.00 | 0.01 |
| GO:0099518 | vesicle cytoskeletal trafficking | 0.00 | 0.01 |
| GO:0002573 | myeloid leukocyte differentiation | 0.00 | 0.01 |
| GO:0034134 | toll-like receptor 2 signaling pathway | 0.00 | 0.01 |
| GO:0060382 | regulation of DNA strand elongation | 0.00 | 0.01 |
| GO:1903830 | magnesium ion transmembrane transport | 0.00 | 0.01 |
| GO:0051298 | centrosome duplication | 0.00 | 0.01 |
| GO:0061515 | myeloid cell development | 0.00 | 0.01 |
| GO:0001510 | RNA methylation | 0.00 | 0.01 |
| GO:0002200 | somatic diversification of immune receptors | 0.00 | 0.01 |
| GO:0006023 | aminoglycan biosynthetic process | 0.00 | 0.01 |
| GO:0097061 | dendritic spine organization | 0.00 | 0.01 |
| GO:0002685 | regulation of leukocyte migration | 0.00 | 0.01 |
| GO:0001894 | tissue homeostasis | 0.00 | 0.01 |
| GO:0001911 | negative regulation of leukocyte mediated cytotoxicity | 0.00 | 0.01 |
| GO:0006929 | substrate-dependent cell migration | 0.00 | 0.01 |
| GO:0032469 | endoplasmic reticulum calcium ion homeostasis | 0.00 | 0.01 |
| GO:0046039 | GTP metabolic process | 0.00 | 0.01 |
| GO:0070423 | nucleotide-binding oligomerization domain containing signaling pathway | 0.00 | 0.01 |
| GO:0071459 | protein localization to chromosome, centromeric region | 0.00 | 0.01 |
| GO:0018107 | peptidyl-threonine phosphorylation | 0.00 | 0.01 |
| GO:0030518 | intracellular steroid hormone receptor signaling pathway | 0.00 | 0.01 |
| GO:0043300 | regulation of leukocyte degranulation | 0.00 | 0.01 |
| GO:2000059 | negative regulation of ubiquitin-dependent protein catabolic process | 0.00 | 0.01 |
| GO:0010950 | positive regulation of endopeptidase activity | 0.00 | 0.01 |
| GO:0045930 | negative regulation of mitotic cell cycle | 0.00 | 0.01 |
| GO:0002683 | negative regulation of immune system process | 0.00 | 0.01 |
| GO:0036092 | phosphatidylinositol-3-phosphate biosynthetic process | 0.00 | 0.01 |
| GO:0044090 | positive regulation of vacuole organization | 0.00 | 0.01 |
| GO:0045056 | transcytosis | 0.00 | 0.01 |
| GO:0071243 | cellular response to arsenic-containing substance | 0.00 | 0.01 |
| GO:0140747 | regulation of ncRNA transcription | 0.00 | 0.01 |
| GO:1903902 | positive regulation of viral life cycle | 0.00 | 0.01 |
| GO:0009595 | detection of biotic stimulus | 0.00 | 0.01 |
| GO:0032506 | cytokinetic process | 0.00 | 0.01 |
| GO:0042073 | intraciliary transport | 0.00 | 0.01 |
| GO:0016054 | organic acid catabolic process | 0.00 | 0.01 |
| GO:0006220 | pyrimidine nucleotide metabolic process | 0.00 | 0.01 |
| GO:0032206 | positive regulation of telomere maintenance | 0.00 | 0.01 |
| GO:0032946 | positive regulation of mononuclear cell proliferation | 0.00 | 0.01 |
| GO:0070661 | leukocyte proliferation | 0.00 | 0.01 |
| GO:0032490 | detection of molecule of bacterial origin | 0.00 | 0.01 |
| GO:1901503 | ether biosynthetic process | 0.00 | 0.01 |
| GO:0043372 | positive regulation of CD4-positive, alpha-beta T cell differentiation | 0.00 | 0.01 |
| GO:0071353 | cellular response to interleukin-4 | 0.00 | 0.01 |
| GO:0032760 | positive regulation of tumor necrosis factor production | 0.00 | 0.01 |
| GO:0042102 | positive regulation of T cell proliferation | 0.00 | 0.01 |
| GO:0001954 | positive regulation of cell-matrix adhesion | 0.00 | 0.01 |
| GO:0097352 | autophagosome maturation | 0.00 | 0.01 |
| GO:0050921 | positive regulation of chemotaxis | 0.00 | 0.01 |
| GO:0098813 | nuclear chromosome segregation | 0.00 | 0.01 |
| GO:0032642 | regulation of chemokine production | 0.00 | 0.01 |
| GO:0051304 | chromosome separation | 0.00 | 0.01 |
| GO:0033619 | membrane protein proteolysis | 0.00 | 0.01 |
| GO:0007099 | centriole replication | 0.00 | 0.01 |
| GO:0034314 | Arp2/3 complex-mediated actin nucleation | 0.00 | 0.01 |
| GO:0045022 | early endosome to late endosome transport | 0.00 | 0.01 |
| GO:1900026 | positive regulation of substrate adhesion-dependent cell spreading | 0.00 | 0.01 |
| GO:0071560 | cellular response to transforming growth factor beta stimulus | 0.00 | 0.01 |
| GO:0032233 | positive regulation of actin filament bundle assembly | 0.00 | 0.01 |
| GO:0043112 | receptor metabolic process | 0.00 | 0.01 |
| GO:0048524 | positive regulation of viral process | 0.00 | 0.01 |
| GO:0061951 | establishment of protein localization to plasma membrane | 0.00 | 0.01 |
| GO:0051236 | establishment of RNA localization | 0.00 | 0.01 |
| GO:0061448 | connective tissue development | 0.00 | 0.01 |
| GO:0032103 | positive regulation of response to external stimulus | 0.00 | 0.01 |
| GO:0006900 | vesicle budding from membrane | 0.00 | 0.01 |
| GO:0001910 | regulation of leukocyte mediated cytotoxicity | 0.00 | 0.01 |
| GO:0071229 | cellular response to acid chemical | 0.00 | 0.01 |
| GO:2000106 | regulation of leukocyte apoptotic process | 0.00 | 0.01 |
| GO:0045787 | positive regulation of cell cycle | 0.00 | 0.01 |
| GO:0002294 | CD4-positive, alpha-beta T cell differentiation involved in immune response | 0.00 | 0.01 |
| GO:0046637 | regulation of alpha-beta T cell differentiation | 0.00 | 0.01 |
| GO:0070936 | protein K48-linked ubiquitination | 0.00 | 0.01 |
| GO:0002064 | epithelial cell development | 0.00 | 0.01 |
| GO:0006606 | protein import into nucleus | 0.00 | 0.01 |
| GO:0050657 | nucleic acid transport | 0.00 | 0.01 |
| GO:0050658 | RNA transport | 0.00 | 0.01 |
| GO:0002562 | somatic diversification of immune receptors via germline recombination within a single locus | 0.00 | 0.01 |
| GO:0016444 | somatic cell DNA recombination | 0.00 | 0.01 |
| GO:0010770 | positive regulation of cell morphogenesis involved in differentiation | 0.00 | 0.01 |
| GO:0006024 | glycosaminoglycan biosynthetic process | 0.00 | 0.01 |
| GO:0043299 | leukocyte degranulation | 0.00 | 0.01 |
| GO:0045619 | regulation of lymphocyte differentiation | 0.00 | 0.01 |
| GO:0006378 | mRNA polyadenylation | 0.00 | 0.01 |
| GO:0019692 | deoxyribose phosphate metabolic process | 0.00 | 0.01 |
| GO:0030835 | negative regulation of actin filament depolymerization | 0.00 | 0.01 |
| GO:0043038 | amino acid activation | 0.00 | 0.01 |
| GO:0046395 | carboxylic acid catabolic process | 0.00 | 0.01 |
| GO:0000460 | maturation of 5.8S rRNA | 0.00 | 0.01 |
| GO:0033146 | regulation of intracellular estrogen receptor signaling pathway | 0.00 | 0.01 |
| GO:0097345 | mitochondrial outer membrane permeabilization | 0.00 | 0.01 |
| GO:0097502 | mannosylation | 0.00 | 0.01 |
| GO:1905898 | positive regulation of response to endoplasmic reticulum stress | 0.00 | 0.01 |
| GO:0031440 | regulation of mRNA 3'-end processing | 0.00 | 0.01 |
| GO:0062125 | regulation of mitochondrial gene expression | 0.00 | 0.01 |
| GO:0090312 | positive regulation of protein deacetylation | 0.00 | 0.01 |
| GO:0051651 | maintenance of location in cell | 0.00 | 0.01 |
| GO:0006958 | complement activation, classical pathway | 0.00 | 0.01 |
| GO:0070663 | regulation of leukocyte proliferation | 0.00 | 0.01 |
| GO:0033865 | nucleoside bisphosphate metabolic process | 0.00 | 0.01 |
| GO:0033875 | ribonucleoside bisphosphate metabolic process | 0.00 | 0.01 |
| GO:0034032 | purine nucleoside bisphosphate metabolic process | 0.00 | 0.01 |
| GO:0010761 | fibroblast migration | 0.00 | 0.01 |
| GO:0032527 | protein exit from endoplasmic reticulum | 0.00 | 0.01 |
| GO:0033628 | regulation of cell adhesion mediated by integrin | 0.00 | 0.01 |
| GO:0035305 | negative regulation of dephosphorylation | 0.00 | 0.01 |
| GO:0060324 | face development | 0.00 | 0.01 |
| GO:0035710 | CD4-positive, alpha-beta T cell activation | 0.00 | 0.01 |
| GO:0010977 | negative regulation of neuron projection development | 0.00 | 0.01 |
| GO:0019083 | viral transcription | 0.00 | 0.01 |
| GO:0030490 | maturation of SSU-rRNA | 0.00 | 0.01 |
| GO:0051496 | positive regulation of stress fiber assembly | 0.00 | 0.01 |
| GO:0071622 | regulation of granulocyte chemotaxis | 0.00 | 0.01 |
| GO:1901991 | negative regulation of mitotic cell cycle phase transition | 0.00 | 0.01 |
| GO:0006270 | DNA replication initiation | 0.00 | 0.01 |
| GO:0072522 | purine-containing compound biosynthetic process | 0.00 | 0.01 |
| GO:0097529 | myeloid leukocyte migration | 0.00 | 0.01 |
| GO:0032528 | microvillus organization | 0.00 | 0.01 |
| GO:0051788 | response to misfolded protein | 0.00 | 0.01 |
| GO:2000209 | regulation of anoikis | 0.00 | 0.01 |
| GO:0006637 | acyl-CoA metabolic process | 0.00 | 0.01 |
| GO:0035383 | thioester metabolic process | 0.00 | 0.01 |
| GO:0030834 | regulation of actin filament depolymerization | 0.00 | 0.01 |
| GO:1901136 | carbohydrate derivative catabolic process | 0.00 | 0.01 |
| GO:0061097 | regulation of protein tyrosine kinase activity | 0.00 | 0.01 |
| GO:0002832 | negative regulation of response to biotic stimulus | 0.00 | 0.01 |
| GO:0006857 | oligopeptide transport | 0.00 | 0.01 |
| GO:0034349 | glial cell apoptotic process | 0.00 | 0.01 |
| GO:0051014 | actin filament severing | 0.00 | 0.01 |
| GO:0070431 | nucleotide-binding oligomerization domain containing 2 signaling pathway | 0.00 | 0.01 |
| GO:0031099 | regeneration | 0.00 | 0.01 |
| GO:1900182 | positive regulation of protein localization to nucleus | 0.00 | 0.01 |
| GO:0061614 | miRNA transcription | 0.00 | 0.01 |
| GO:0000002 | mitochondrial genome maintenance | 0.00 | 0.01 |
| GO:0001782 | B cell homeostasis | 0.00 | 0.01 |
| GO:0035066 | positive regulation of histone acetylation | 0.00 | 0.01 |
| GO:0140718 | facultative heterochromatin assembly | 0.00 | 0.01 |
| GO:0150117 | positive regulation of cell-substrate junction organization | 0.00 | 0.01 |
| GO:1900027 | regulation of ruffle assembly | 0.00 | 0.01 |
| GO:2000406 | positive regulation of T cell migration | 0.00 | 0.01 |
| GO:0010833 | telomere maintenance via telomere lengthening | 0.00 | 0.01 |
| GO:0038061 | NIK/NF-kappaB signaling | 0.00 | 0.01 |
| GO:0002396 | MHC protein complex assembly | 0.00 | 0.01 |
| GO:0002501 | peptide antigen assembly with MHC protein complex | 0.00 | 0.01 |
| GO:0048025 | negative regulation of mRNA splicing, via spliceosome | 0.00 | 0.01 |
| GO:0070584 | mitochondrion morphogenesis | 0.00 | 0.01 |
| GO:0097320 | plasma membrane tubulation | 0.00 | 0.01 |
| GO:1901659 | glycosyl compound biosynthetic process | 0.00 | 0.01 |
| GO:0008088 | axo-dendritic transport | 0.00 | 0.01 |
| GO:0006418 | tRNA aminoacylation for protein translation | 0.00 | 0.01 |
| GO:0031297 | replication fork processing | 0.00 | 0.01 |
| GO:0010822 | positive regulation of mitochondrion organization | 0.00 | 0.01 |
| GO:0071230 | cellular response to amino acid stimulus | 0.00 | 0.01 |
| GO:0001736 | establishment of planar polarity | 0.00 | 0.01 |
| GO:0007164 | establishment of tissue polarity | 0.00 | 0.01 |
| GO:0033045 | regulation of sister chromatid segregation | 0.00 | 0.01 |
| GO:0033143 | regulation of intracellular steroid hormone receptor signaling pathway | 0.00 | 0.01 |
| GO:0035567 | non-canonical Wnt signaling pathway | 0.00 | 0.01 |
| GO:0009145 | purine nucleoside triphosphate biosynthetic process | 0.00 | 0.01 |
| GO:0090501 | RNA phosphodiester bond hydrolysis | 0.00 | 0.01 |
| GO:0018210 | peptidyl-threonine modification | 0.00 | 0.01 |
| GO:0072329 | monocarboxylic acid catabolic process | 0.00 | 0.01 |
| GO:0044262 | cellular carbohydrate metabolic process | 0.00 | 0.01 |
| GO:0110020 | regulation of actomyosin structure organization | 0.00 | 0.01 |
| GO:0009124 | nucleoside monophosphate biosynthetic process | 0.00 | 0.01 |
| GO:0009126 | purine nucleoside monophosphate metabolic process | 0.00 | 0.01 |
| GO:0009262 | deoxyribonucleotide metabolic process | 0.00 | 0.01 |
| GO:0043039 | tRNA aminoacylation | 0.00 | 0.01 |
| GO:0061001 | regulation of dendritic spine morphogenesis | 0.00 | 0.01 |
| GO:0001667 | ameboidal-type cell migration | 0.00 | 0.01 |
| GO:0008611 | ether lipid biosynthetic process | 0.00 | 0.01 |
| GO:0009120 | deoxyribonucleoside metabolic process | 0.00 | 0.01 |
| GO:0045628 | regulation of T-helper 2 cell differentiation | 0.00 | 0.01 |
| GO:0046504 | glycerol ether biosynthetic process | 0.00 | 0.01 |
| GO:0046598 | positive regulation of viral entry into host cell | 0.00 | 0.01 |
| GO:0046643 | regulation of gamma-delta T cell activation | 0.00 | 0.01 |
| GO:0070561 | vitamin D receptor signaling pathway | 0.00 | 0.01 |
| GO:0072697 | protein localization to cell cortex | 0.00 | 0.01 |
| GO:0075294 | positive regulation by symbiont of entry into host | 0.00 | 0.01 |
| GO:0097384 | cellular lipid biosynthetic process | 0.00 | 0.01 |
| GO:2000392 | regulation of lamellipodium morphogenesis | 0.00 | 0.01 |
| GO:0006891 | intra-Golgi vesicle-mediated transport | 0.00 | 0.01 |
| GO:0009303 | rRNA transcription | 0.00 | 0.01 |
| GO:0032212 | positive regulation of telomere maintenance via telomerase | 0.00 | 0.01 |
| GO:0051125 | regulation of actin nucleation | 0.00 | 0.01 |
| GO:0070670 | response to interleukin-4 | 0.00 | 0.01 |
| GO:0051972 | regulation of telomerase activity | 0.00 | 0.01 |
| GO:0009060 | aerobic respiration | 0.00 | 0.01 |
| GO:0006888 | endoplasmic reticulum to Golgi vesicle-mediated transport | 0.00 | 0.01 |
| GO:0018126 | protein hydroxylation | 0.00 | 0.01 |
| GO:0039529 | RIG-I signaling pathway | 0.00 | 0.01 |
| GO:0046174 | polyol catabolic process | 0.00 | 0.01 |
| GO:1904376 | negative regulation of protein localization to cell periphery | 0.00 | 0.01 |
| GO:0106027 | neuron projection organization | 0.00 | 0.01 |
| GO:0031057 | negative regulation of histone modification | 0.00 | 0.01 |
| GO:0038202 | TORC1 signaling | 0.00 | 0.01 |
| GO:0043457 | regulation of cellular respiration | 0.00 | 0.01 |
| GO:0072523 | purine-containing compound catabolic process | 0.00 | 0.01 |
| GO:0090174 | organelle membrane fusion | 0.00 | 0.01 |
| GO:0045580 | regulation of T cell differentiation | 0.00 | 0.01 |
| GO:0030198 | extracellular matrix organization | 0.00 | 0.01 |
| GO:0032210 | regulation of telomere maintenance via telomerase | 0.00 | 0.01 |
| GO:0007179 | transforming growth factor beta receptor signaling pathway | 0.00 | 0.01 |
| GO:0006623 | protein targeting to vacuole | 0.00 | 0.01 |
| GO:0010923 | negative regulation of phosphatase activity | 0.00 | 0.01 |
| GO:1904358 | positive regulation of telomere maintenance via telomere lengthening | 0.00 | 0.01 |
| GO:0000380 | alternative mRNA splicing, via spliceosome | 0.00 | 0.01 |
| GO:0035773 | insulin secretion involved in cellular response to glucose stimulus | 0.00 | 0.01 |
| GO:1902893 | regulation of miRNA transcription | 0.00 | 0.01 |
| GO:0035335 | peptidyl-tyrosine dephosphorylation | 0.00 | 0.01 |
| GO:0006352 | DNA-templated transcription, initiation | 0.00 | 0.01 |
| GO:0019079 | viral genome replication | 0.00 | 0.01 |
| GO:0042255 | ribosome assembly | 0.00 | 0.01 |
| GO:0002287 | alpha-beta T cell activation involved in immune response | 0.00 | 0.01 |
| GO:0002293 | alpha-beta T cell differentiation involved in immune response | 0.00 | 0.01 |
| GO:0009206 | purine ribonucleoside triphosphate biosynthetic process | 0.00 | 0.01 |
| GO:0002260 | lymphocyte homeostasis | 0.00 | 0.01 |
| GO:0010965 | regulation of mitotic sister chromatid separation | 0.00 | 0.01 |
| GO:0042093 | T-helper cell differentiation | 0.00 | 0.01 |
| GO:1903051 | negative regulation of proteolysis involved in cellular protein catabolic process | 0.00 | 0.01 |
| GO:0002367 | cytokine production involved in immune response | 0.00 | 0.01 |
| GO:0046939 | nucleotide phosphorylation | 0.00 | 0.01 |
| GO:0043968 | histone H2A acetylation | 0.00 | 0.01 |
| GO:0046641 | positive regulation of alpha-beta T cell proliferation | 0.00 | 0.01 |
| GO:0000266 | mitochondrial fission | 0.00 | 0.01 |
| GO:0035329 | hippo signaling | 0.00 | 0.01 |
| GO:0019886 | antigen processing and presentation of exogenous peptide antigen via MHC class II | 0.00 | 0.01 |
| GO:0043094 | cellular metabolic compound salvage | 0.00 | 0.01 |
| GO:0090140 | regulation of mitochondrial fission | 0.00 | 0.01 |
| GO:0071621 | granulocyte chemotaxis | 0.00 | 0.01 |
| GO:0001738 | morphogenesis of a polarized epithelium | 0.00 | 0.02 |
| GO:0033108 | mitochondrial respiratory chain complex assembly | 0.00 | 0.02 |
| GO:0051783 | regulation of nuclear division | 0.00 | 0.02 |
| GO:0006284 | base-excision repair | 0.00 | 0.02 |
| GO:0009394 | 2'-deoxyribonucleotide metabolic process | 0.00 | 0.02 |
| GO:0044782 | cilium organization | 0.00 | 0.02 |
| GO:0033127 | regulation of histone phosphorylation | 0.00 | 0.02 |
| GO:0033599 | regulation of mammary gland epithelial cell proliferation | 0.00 | 0.02 |
| GO:0043517 | positive regulation of DNA damage response, signal transduction by p53 class mediator | 0.00 | 0.02 |
| GO:0051299 | centrosome separation | 0.00 | 0.02 |
| GO:1900029 | positive regulation of ruffle assembly | 0.00 | 0.02 |
| GO:1901836 | regulation of transcription of nucleolar large rRNA by RNA polymerase I | 0.00 | 0.02 |
| GO:1902287 | semaphorin-plexin signaling pathway involved in axon guidance | 0.00 | 0.02 |
| GO:0043062 | extracellular structure organization | 0.00 | 0.02 |
| GO:0002283 | neutrophil activation involved in immune response | 0.00 | 0.02 |
| GO:0002483 | antigen processing and presentation of endogenous peptide antigen | 0.00 | 0.02 |
| GO:0032930 | positive regulation of superoxide anion generation | 0.00 | 0.02 |
| GO:0046782 | regulation of viral transcription | 0.00 | 0.02 |
| GO:2001135 | regulation of endocytic recycling | 0.00 | 0.02 |
| GO:2001185 | regulation of CD8-positive, alpha-beta T cell activation | 0.00 | 0.02 |
| GO:0006575 | cellular modified amino acid metabolic process | 0.00 | 0.02 |
| GO:0043488 | regulation of mRNA stability | 0.00 | 0.02 |
| GO:0035794 | positive regulation of mitochondrial membrane permeability | 0.00 | 0.02 |
| GO:0043631 | RNA polyadenylation | 0.00 | 0.02 |
| GO:0060271 | cilium assembly | 0.00 | 0.02 |
| GO:0060070 | canonical Wnt signaling pathway | 0.00 | 0.02 |
| GO:0072332 | intrinsic apoptotic signaling pathway by p53 class mediator | 0.00 | 0.02 |
| GO:0003382 | epithelial cell morphogenesis | 0.00 | 0.02 |
| GO:0006084 | acetyl-CoA metabolic process | 0.00 | 0.02 |
| GO:0009225 | nucleotide-sugar metabolic process | 0.00 | 0.02 |
| GO:0036475 | neuron death in response to oxidative stress | 0.00 | 0.02 |
| GO:0046685 | response to arsenic-containing substance | 0.00 | 0.02 |
| GO:0090022 | regulation of neutrophil chemotaxis | 0.00 | 0.02 |
| GO:0006910 | phagocytosis, recognition | 0.00 | 0.02 |
| GO:0045005 | DNA-templated DNA replication maintenance of fidelity | 0.00 | 0.02 |
| GO:0045229 | external encapsulating structure organization | 0.00 | 0.02 |
| GO:0002204 | somatic recombination of immunoglobulin genes involved in immune response | 0.00 | 0.02 |
| GO:0002208 | somatic diversification of immunoglobulins involved in immune response | 0.00 | 0.02 |
| GO:0045190 | isotype switching | 0.00 | 0.02 |
| GO:0002285 | lymphocyte activation involved in immune response | 0.00 | 0.02 |
| GO:0006165 | nucleoside diphosphate phosphorylation | 0.00 | 0.02 |
| GO:0006487 | protein N-linked glycosylation | 0.00 | 0.02 |
| GO:0045010 | actin nucleation | 0.00 | 0.02 |
| GO:0002704 | negative regulation of leukocyte mediated immunity | 0.00 | 0.02 |
| GO:0072678 | T cell migration | 0.00 | 0.02 |
| GO:0060348 | bone development | 0.00 | 0.02 |
| GO:0006289 | nucleotide-excision repair | 0.00 | 0.02 |
| GO:0001649 | osteoblast differentiation | 0.00 | 0.02 |
| GO:0001885 | endothelial cell development | 0.00 | 0.02 |
| GO:0009116 | nucleoside metabolic process | 0.00 | 0.02 |
| GO:0032507 | maintenance of protein location in cell | 0.00 | 0.02 |
| GO:0034340 | response to type I interferon | 0.00 | 0.02 |
| GO:0016226 | iron-sulfur cluster assembly | 0.00 | 0.02 |
| GO:0031163 | metallo-sulfur cluster assembly | 0.00 | 0.02 |
| GO:0032515 | negative regulation of phosphoprotein phosphatase activity | 0.00 | 0.02 |
| GO:2000050 | regulation of non-canonical Wnt signaling pathway | 0.00 | 0.02 |
| GO:0044272 | sulfur compound biosynthetic process | 0.00 | 0.02 |
| GO:0032435 | negative regulation of proteasomal ubiquitin-dependent protein catabolic process | 0.00 | 0.02 |
| GO:0042789 | mRNA transcription by RNA polymerase II | 0.00 | 0.02 |
| GO:0045652 | regulation of megakaryocyte differentiation | 0.00 | 0.02 |
| GO:0045742 | positive regulation of epidermal growth factor receptor signaling pathway | 0.00 | 0.02 |
| GO:1903749 | positive regulation of establishment of protein localization to mitochondrion | 0.00 | 0.02 |
| GO:0034109 | homotypic cell-cell adhesion | 0.00 | 0.02 |
| GO:0051216 | cartilage development | 0.00 | 0.02 |
| GO:0044242 | cellular lipid catabolic process | 0.00 | 0.02 |
| GO:1901216 | positive regulation of neuron death | 0.00 | 0.02 |
| GO:0030953 | astral microtubule organization | 0.00 | 0.02 |
| GO:0031118 | rRNA pseudouridine synthesis | 0.00 | 0.02 |
| GO:0032060 | bleb assembly | 0.00 | 0.02 |
| GO:0060245 | detection of cell density | 0.00 | 0.02 |
| GO:0060368 | regulation of Fc receptor mediated stimulatory signaling pathway | 0.00 | 0.02 |
| GO:0070391 | response to lipoteichoic acid | 0.00 | 0.02 |
| GO:0071223 | cellular response to lipoteichoic acid | 0.00 | 0.02 |
| GO:0150172 | regulation of phosphatidylcholine metabolic process | 0.00 | 0.02 |
| GO:1902275 | regulation of chromatin organization | 0.00 | 0.02 |
| GO:0030010 | establishment of cell polarity | 0.00 | 0.02 |
| GO:0035264 | multicellular organism growth | 0.00 | 0.02 |
| GO:0006520 | cellular amino acid metabolic process | 0.00 | 0.02 |
| GO:0048017 | inositol lipid-mediated signaling | 0.00 | 0.02 |
| GO:0006354 | DNA-templated transcription, elongation | 0.00 | 0.02 |
| GO:0051303 | establishment of chromosome localization | 0.00 | 0.02 |
| GO:0001101 | response to acid chemical | 0.00 | 0.02 |
| GO:0070997 | neuron death | 0.00 | 0.02 |
| GO:0030970 | retrograde protein transport, ER to cytosol | 0.00 | 0.02 |
| GO:0031571 | mitotic G1 DNA damage checkpoint signaling | 0.00 | 0.02 |
| GO:0034067 | protein localization to Golgi apparatus | 0.00 | 0.02 |
| GO:1903513 | endoplasmic reticulum to cytosol transport | 0.00 | 0.02 |
| GO:0009167 | purine ribonucleoside monophosphate metabolic process | 0.00 | 0.02 |
| GO:0002363 | alpha-beta T cell lineage commitment | 0.00 | 0.02 |
| GO:0006662 | glycerol ether metabolic process | 0.00 | 0.02 |
| GO:0032928 | regulation of superoxide anion generation | 0.00 | 0.02 |
| GO:0071218 | cellular response to misfolded protein | 0.00 | 0.02 |
| GO:0090042 | tubulin deacetylation | 0.00 | 0.02 |
| GO:0031497 | chromatin assembly | 0.00 | 0.02 |
| GO:0002718 | regulation of cytokine production involved in immune response | 0.00 | 0.02 |
| GO:1903532 | positive regulation of secretion by cell | 0.01 | 0.02 |
| GO:0008631 | intrinsic apoptotic signaling pathway in response to oxidative stress | 0.01 | 0.02 |
| GO:0031295 | T cell costimulation | 0.01 | 0.02 |
| GO:0033003 | regulation of mast cell activation | 0.01 | 0.02 |
| GO:1902895 | positive regulation of miRNA transcription | 0.01 | 0.02 |
| GO:2000273 | positive regulation of signaling receptor activity | 0.01 | 0.02 |
| GO:0042246 | tissue regeneration | 0.01 | 0.02 |
| GO:0042273 | ribosomal large subunit biogenesis | 0.01 | 0.02 |
| GO:0022900 | electron transport chain | 0.01 | 0.02 |
| GO:0061028 | establishment of endothelial barrier | 0.01 | 0.02 |
| GO:0034394 | protein localization to cell surface | 0.01 | 0.02 |
| GO:0090307 | mitotic spindle assembly | 0.01 | 0.02 |
| GO:0007080 | mitotic metaphase plate congression | 0.01 | 0.02 |
| GO:0010823 | negative regulation of mitochondrion organization | 0.01 | 0.02 |
| GO:1905710 | positive regulation of membrane permeability | 0.01 | 0.02 |
| GO:0000288 | nuclear-transcribed mRNA catabolic process, deadenylation-dependent decay | 0.01 | 0.02 |
| GO:0006767 | water-soluble vitamin metabolic process | 0.01 | 0.02 |
| GO:0032613 | interleukin-10 production | 0.01 | 0.02 |
| GO:0032653 | regulation of interleukin-10 production | 0.01 | 0.02 |
| GO:0071806 | protein transmembrane transport | 0.01 | 0.02 |
| GO:0030520 | intracellular estrogen receptor signaling pathway | 0.01 | 0.02 |
| GO:0046824 | positive regulation of nucleocytoplasmic transport | 0.01 | 0.02 |
| GO:0051205 | protein insertion into membrane | 0.01 | 0.02 |
| GO:0006998 | nuclear envelope organization | 0.01 | 0.02 |
| GO:0032608 | interferon-beta production | 0.01 | 0.02 |
| GO:0032648 | regulation of interferon-beta production | 0.01 | 0.02 |
| GO:0048278 | vesicle docking | 0.01 | 0.02 |
| GO:0070534 | protein K63-linked ubiquitination | 0.01 | 0.02 |
| GO:0090175 | regulation of establishment of planar polarity | 0.01 | 0.02 |
| GO:0019082 | viral protein processing | 0.01 | 0.02 |
| GO:1901185 | negative regulation of ERBB signaling pathway | 0.01 | 0.02 |
| GO:1903955 | positive regulation of protein targeting to mitochondrion | 0.01 | 0.02 |
| GO:0006493 | protein O-linked glycosylation | 0.01 | 0.02 |
| GO:0042790 | nucleolar large rRNA transcription by RNA polymerase I | 0.01 | 0.02 |
| GO:0002312 | B cell activation involved in immune response | 0.01 | 0.02 |
| GO:0031123 | RNA 3'-end processing | 0.01 | 0.02 |
| GO:0017157 | regulation of exocytosis | 0.01 | 0.02 |
| GO:0000054 | ribosomal subunit export from nucleus | 0.01 | 0.02 |
| GO:0002475 | antigen processing and presentation via MHC class Ib | 0.01 | 0.02 |
| GO:0007100 | mitotic centrosome separation | 0.01 | 0.02 |
| GO:0007175 | negative regulation of epidermal growth factor-activated receptor activity | 0.01 | 0.02 |
| GO:0033750 | ribosome localization | 0.01 | 0.02 |
| GO:0035672 | oligopeptide transmembrane transport | 0.01 | 0.02 |
| GO:0051764 | actin crosslink formation | 0.01 | 0.02 |
| GO:0061709 | reticulophagy | 0.01 | 0.02 |
| GO:0090239 | regulation of histone H4 acetylation | 0.01 | 0.02 |
| GO:0033273 | response to vitamin | 0.01 | 0.02 |
| GO:0045921 | positive regulation of exocytosis | 0.01 | 0.02 |
| GO:0050000 | chromosome localization | 0.01 | 0.02 |
| GO:0070665 | positive regulation of leukocyte proliferation | 0.01 | 0.02 |
| GO:0043200 | response to amino acid | 0.01 | 0.02 |
| GO:0034612 | response to tumor necrosis factor | 0.01 | 0.02 |
| GO:0009081 | branched-chain amino acid metabolic process | 0.01 | 0.02 |
| GO:0009164 | nucleoside catabolic process | 0.01 | 0.02 |
| GO:1903077 | negative regulation of protein localization to plasma membrane | 0.01 | 0.02 |
| GO:0050920 | regulation of chemotaxis | 0.01 | 0.02 |
| GO:0010389 | regulation of G2/M transition of mitotic cell cycle | 0.01 | 0.02 |
| GO:2001237 | negative regulation of extrinsic apoptotic signaling pathway | 0.01 | 0.02 |
| GO:0010634 | positive regulation of epithelial cell migration | 0.01 | 0.02 |
| GO:0030178 | negative regulation of Wnt signaling pathway | 0.01 | 0.02 |
| GO:0032729 | positive regulation of interferon-gamma production | 0.01 | 0.02 |
| GO:0002478 | antigen processing and presentation of exogenous peptide antigen | 0.01 | 0.02 |
| GO:0035337 | fatty-acyl-CoA metabolic process | 0.01 | 0.02 |
| GO:1901186 | positive regulation of ERBB signaling pathway | 0.01 | 0.02 |
| GO:1901661 | quinone metabolic process | 0.01 | 0.02 |
| GO:0002637 | regulation of immunoglobulin production | 0.01 | 0.02 |
| GO:0007040 | lysosome organization | 0.01 | 0.02 |
| GO:0009201 | ribonucleoside triphosphate biosynthetic process | 0.01 | 0.02 |
| GO:0080171 | lytic vacuole organization | 0.01 | 0.02 |
| GO:0003158 | endothelium development | 0.01 | 0.02 |
| GO:0002690 | positive regulation of leukocyte chemotaxis | 0.01 | 0.02 |
| GO:1902749 | regulation of cell cycle G2/M phase transition | 0.01 | 0.02 |
| GO:0006040 | amino sugar metabolic process | 0.01 | 0.03 |
| GO:0009154 | purine ribonucleotide catabolic process | 0.01 | 0.03 |
| GO:0032733 | positive regulation of interleukin-10 production | 0.01 | 0.03 |
| GO:0051693 | actin filament capping | 0.01 | 0.03 |
| GO:0050777 | negative regulation of immune response | 0.01 | 0.03 |
| GO:0048525 | negative regulation of viral process | 0.01 | 0.03 |
| GO:0051983 | regulation of chromosome segregation | 0.01 | 0.03 |
| GO:0046578 | regulation of Ras protein signal transduction | 0.01 | 0.03 |
| GO:0060249 | anatomical structure homeostasis | 0.01 | 0.03 |
| GO:0006906 | vesicle fusion | 0.01 | 0.03 |
| GO:0045576 | mast cell activation | 0.01 | 0.03 |
| GO:0006509 | membrane protein ectodomain proteolysis | 0.01 | 0.03 |
| GO:1903432 | regulation of TORC1 signaling | 0.01 | 0.03 |
| GO:1903573 | negative regulation of response to endoplasmic reticulum stress | 0.01 | 0.03 |
| GO:2000404 | regulation of T cell migration | 0.01 | 0.03 |
| GO:0061564 | axon development | 0.01 | 0.03 |
| GO:0034644 | cellular response to UV | 0.01 | 0.03 |
| GO:0010972 | negative regulation of G2/M transition of mitotic cell cycle | 0.01 | 0.03 |
| GO:0046902 | regulation of mitochondrial membrane permeability | 0.01 | 0.03 |
| GO:0043406 | positive regulation of MAP kinase activity | 0.01 | 0.03 |
| GO:0019076 | viral release from host cell | 0.01 | 0.03 |
| GO:0031342 | negative regulation of cell killing | 0.01 | 0.03 |
| GO:0035890 | exit from host | 0.01 | 0.03 |
| GO:0035891 | exit from host cell | 0.01 | 0.03 |
| GO:0042059 | negative regulation of epidermal growth factor receptor signaling pathway | 0.01 | 0.03 |
| GO:0045830 | positive regulation of isotype switching | 0.01 | 0.03 |
| GO:0031294 | lymphocyte costimulation | 0.01 | 0.03 |
| GO:0036230 | granulocyte activation | 0.01 | 0.03 |
| GO:0009161 | ribonucleoside monophosphate metabolic process | 0.01 | 0.03 |
| GO:0043370 | regulation of CD4-positive, alpha-beta T cell differentiation | 0.01 | 0.03 |
| GO:0051646 | mitochondrion localization | 0.01 | 0.03 |
| GO:0061178 | regulation of insulin secretion involved in cellular response to glucose stimulus | 0.01 | 0.03 |
| GO:0061647 | histone H3-K9 modification | 0.01 | 0.03 |
| GO:1901799 | negative regulation of proteasomal protein catabolic process | 0.01 | 0.03 |
| GO:1903747 | regulation of establishment of protein localization to mitochondrion | 0.01 | 0.03 |
| GO:0030838 | positive regulation of actin filament polymerization | 0.01 | 0.03 |
| GO:0060071 | Wnt signaling pathway, planar cell polarity pathway | 0.01 | 0.03 |
| GO:0006164 | purine nucleotide biosynthetic process | 0.01 | 0.03 |
| GO:0050764 | regulation of phagocytosis | 0.01 | 0.03 |
| GO:0071356 | cellular response to tumor necrosis factor | 0.01 | 0.03 |
| GO:0006450 | regulation of translational fidelity | 0.01 | 0.03 |
| GO:0007252 | I-kappaB phosphorylation | 0.01 | 0.03 |
| GO:0009083 | branched-chain amino acid catabolic process | 0.01 | 0.03 |
| GO:0035330 | regulation of hippo signaling | 0.01 | 0.03 |
| GO:0051767 | nitric-oxide synthase biosynthetic process | 0.01 | 0.03 |
| GO:0051769 | regulation of nitric-oxide synthase biosynthetic process | 0.01 | 0.03 |
| GO:1901652 | response to peptide | 0.01 | 0.03 |
| GO:1901222 | regulation of NIK/NF-kappaB signaling | 0.01 | 0.03 |
| GO:0014068 | positive regulation of phosphatidylinositol 3-kinase signaling | 0.01 | 0.03 |
| GO:0010039 | response to iron ion | 0.01 | 0.03 |
| GO:0002292 | T cell differentiation involved in immune response | 0.01 | 0.03 |
| GO:0031349 | positive regulation of defense response | 0.01 | 0.03 |
| GO:0033209 | tumor necrosis factor-mediated signaling pathway | 0.01 | 0.03 |
| GO:0007224 | smoothened signaling pathway | 0.01 | 0.03 |
| GO:2000027 | regulation of animal organ morphogenesis | 0.01 | 0.03 |
| GO:0072593 | reactive oxygen species metabolic process | 0.01 | 0.03 |
| GO:0060538 | skeletal muscle organ development | 0.01 | 0.03 |
| GO:0036465 | synaptic vesicle recycling | 0.01 | 0.03 |
| GO:0045824 | negative regulation of innate immune response | 0.01 | 0.03 |
| GO:0021696 | cerebellar cortex morphogenesis | 0.01 | 0.03 |
| GO:0033006 | regulation of mast cell activation involved in immune response | 0.01 | 0.03 |
| GO:0035520 | monoubiquitinated protein deubiquitination | 0.01 | 0.03 |
| GO:1901657 | glycosyl compound metabolic process | 0.01 | 0.03 |
| GO:0032722 | positive regulation of chemokine production | 0.01 | 0.03 |
| GO:0035023 | regulation of Rho protein signal transduction | 0.01 | 0.03 |
| GO:0045069 | regulation of viral genome replication | 0.01 | 0.03 |
| GO:0001845 | phagolysosome assembly | 0.01 | 0.03 |
| GO:0002830 | positive regulation of type 2 immune response | 0.01 | 0.03 |
| GO:0006390 | mitochondrial transcription | 0.01 | 0.03 |
| GO:0006895 | Golgi to endosome transport | 0.01 | 0.03 |
| GO:0006978 | DNA damage response, signal transduction by p53 class mediator resulting in transcription of p21 class mediator | 0.01 | 0.03 |
| GO:0019885 | antigen processing and presentation of endogenous peptide antigen via MHC class I | 0.01 | 0.03 |
| GO:0033623 | regulation of integrin activation | 0.01 | 0.03 |
| GO:0035269 | protein O-linked mannosylation | 0.01 | 0.03 |
| GO:0043923 | positive regulation by host of viral transcription | 0.01 | 0.03 |
| GO:0046838 | phosphorylated carbohydrate dephosphorylation | 0.01 | 0.03 |
| GO:0046855 | inositol phosphate dephosphorylation | 0.01 | 0.03 |
| GO:0051770 | positive regulation of nitric-oxide synthase biosynthetic process | 0.01 | 0.03 |
| GO:0051895 | negative regulation of focal adhesion assembly | 0.01 | 0.03 |
| GO:0071816 | tail-anchored membrane protein insertion into ER membrane | 0.01 | 0.03 |
| GO:0090083 | regulation of inclusion body assembly | 0.01 | 0.03 |
| GO:0150118 | negative regulation of cell-substrate junction organization | 0.01 | 0.03 |
| GO:1903358 | regulation of Golgi organization | 0.01 | 0.03 |
| GO:2000811 | negative regulation of anoikis | 0.01 | 0.03 |
| GO:1902882 | regulation of response to oxidative stress | 0.01 | 0.03 |
| GO:0009266 | response to temperature stimulus | 0.01 | 0.03 |
| GO:0000154 | rRNA modification | 0.01 | 0.03 |
| GO:0006739 | NADP metabolic process | 0.01 | 0.03 |
| GO:0022616 | DNA strand elongation | 0.01 | 0.03 |
| GO:0031112 | positive regulation of microtubule polymerization or depolymerization | 0.01 | 0.03 |
| GO:0045730 | respiratory burst | 0.01 | 0.03 |
| GO:0048821 | erythrocyte development | 0.01 | 0.03 |
| GO:0072528 | pyrimidine-containing compound biosynthetic process | 0.01 | 0.03 |
| GO:0098930 | axonal transport | 0.01 | 0.03 |
| GO:1902750 | negative regulation of cell cycle G2/M phase transition | 0.01 | 0.03 |
| GO:0000466 | maturation of 5.8S rRNA from tricistronic rRNA transcript (SSU-rRNA, 5.8S rRNA, LSU-rRNA) | 0.01 | 0.03 |
| GO:0034315 | regulation of Arp2/3 complex-mediated actin nucleation | 0.01 | 0.03 |
| GO:0039531 | regulation of viral-induced cytoplasmic pattern recognition receptor signaling pathway | 0.01 | 0.03 |
| GO:0070129 | regulation of mitochondrial translation | 0.01 | 0.03 |
| GO:0070841 | inclusion body assembly | 0.01 | 0.03 |
| GO:0090023 | positive regulation of neutrophil chemotaxis | 0.01 | 0.03 |
| GO:1904666 | regulation of ubiquitin protein ligase activity | 0.01 | 0.03 |
| GO:0009185 | ribonucleoside diphosphate metabolic process | 0.01 | 0.03 |
| GO:0001952 | regulation of cell-matrix adhesion | 0.01 | 0.03 |
| GO:0010906 | regulation of glucose metabolic process | 0.01 | 0.03 |
| GO:1904356 | regulation of telomere maintenance via telomere lengthening | 0.01 | 0.03 |
| GO:0016233 | telomere capping | 0.01 | 0.03 |
| GO:0097009 | energy homeostasis | 0.01 | 0.03 |
| GO:0036473 | cell death in response to oxidative stress | 0.01 | 0.03 |
| GO:0090090 | negative regulation of canonical Wnt signaling pathway | 0.01 | 0.03 |
| GO:0006611 | protein export from nucleus | 0.01 | 0.03 |
| GO:0032481 | positive regulation of type I interferon production | 0.01 | 0.03 |
| GO:0048015 | phosphatidylinositol-mediated signaling | 0.01 | 0.03 |
| GO:0034661 | ncRNA catabolic process | 0.01 | 0.03 |
| GO:0046596 | regulation of viral entry into host cell | 0.01 | 0.03 |
| GO:1902686 | mitochondrial outer membrane permeabilization involved in programmed cell death | 0.01 | 0.03 |
| GO:0002707 | negative regulation of lymphocyte mediated immunity | 0.01 | 0.03 |
| GO:0006414 | translational elongation | 0.01 | 0.03 |
| GO:0060998 | regulation of dendritic spine development | 0.01 | 0.03 |
| GO:0009261 | ribonucleotide catabolic process | 0.01 | 0.03 |
| GO:0042398 | cellular modified amino acid biosynthetic process | 0.01 | 0.03 |
| GO:0085029 | extracellular matrix assembly | 0.01 | 0.03 |
| GO:0090199 | regulation of release of cytochrome c from mitochondria | 0.01 | 0.03 |
| GO:0046605 | regulation of centrosome cycle | 0.01 | 0.03 |
| GO:0065002 | intracellular protein transmembrane transport | 0.01 | 0.03 |
| GO:0009133 | nucleoside diphosphate biosynthetic process | 0.01 | 0.03 |
| GO:0010771 | negative regulation of cell morphogenesis involved in differentiation | 0.01 | 0.03 |
| GO:0015801 | aromatic amino acid transport | 0.01 | 0.03 |
| GO:0022038 | corpus callosum development | 0.01 | 0.03 |
| GO:0032530 | regulation of microvillus organization | 0.01 | 0.03 |
| GO:0043928 | exonucleolytic catabolism of deadenylated mRNA | 0.01 | 0.03 |
| GO:0090161 | Golgi ribbon formation | 0.01 | 0.03 |
| GO:0098792 | xenophagy | 0.01 | 0.03 |
| GO:1900025 | negative regulation of substrate adhesion-dependent cell spreading | 0.01 | 0.03 |
| GO:0031146 | SCF-dependent proteasomal ubiquitin-dependent protein catabolic process | 0.01 | 0.03 |
| GO:0051489 | regulation of filopodium assembly | 0.01 | 0.03 |
| GO:0070482 | response to oxygen levels | 0.01 | 0.03 |
| GO:0006119 | oxidative phosphorylation | 0.01 | 0.03 |
| GO:1903533 | regulation of protein targeting | 0.01 | 0.03 |
| GO:0043410 | positive regulation of MAPK cascade | 0.01 | 0.03 |
| GO:0042116 | macrophage activation | 0.01 | 0.03 |
| GO:0034614 | cellular response to reactive oxygen species | 0.01 | 0.03 |
| GO:0007519 | skeletal muscle tissue development | 0.01 | 0.03 |
| GO:0006278 | RNA-templated DNA biosynthetic process | 0.01 | 0.03 |
| GO:0070227 | lymphocyte apoptotic process | 0.01 | 0.03 |
| GO:0000469 | cleavage involved in rRNA processing | 0.01 | 0.03 |
| GO:0006515 | protein quality control for misfolded or incompletely synthesized proteins | 0.01 | 0.03 |
| GO:0018904 | ether metabolic process | 0.01 | 0.03 |
| GO:0034453 | microtubule anchoring | 0.01 | 0.03 |
| GO:0050687 | negative regulation of defense response to virus | 0.01 | 0.03 |
| GO:0071624 | positive regulation of granulocyte chemotaxis | 0.01 | 0.03 |
| GO:1903055 | positive regulation of extracellular matrix organization | 0.01 | 0.03 |
| GO:0048863 | stem cell differentiation | 0.01 | 0.03 |
| GO:0034767 | positive regulation of ion transmembrane transport | 0.01 | 0.03 |
| GO:1901214 | regulation of neuron death | 0.01 | 0.03 |
| GO:1903578 | regulation of ATP metabolic process | 0.01 | 0.03 |
| GO:2001251 | negative regulation of chromosome organization | 0.01 | 0.03 |
| GO:0006801 | superoxide metabolic process | 0.01 | 0.03 |
| GO:0050866 | negative regulation of cell activation | 0.01 | 0.04 |
| GO:0071824 | protein-DNA complex subunit organization | 0.01 | 0.04 |
| GO:0071901 | negative regulation of protein serine/threonine kinase activity | 0.01 | 0.04 |
| GO:0014066 | regulation of phosphatidylinositol 3-kinase signaling | 0.01 | 0.04 |
| GO:0006734 | NADH metabolic process | 0.01 | 0.04 |
| GO:0010847 | regulation of chromatin assembly | 0.01 | 0.04 |
| GO:0034656 | nucleobase-containing small molecule catabolic process | 0.01 | 0.04 |
| GO:0044819 | mitotic G1/S transition checkpoint signaling | 0.01 | 0.04 |
| GO:0070979 | protein K11-linked ubiquitination | 0.01 | 0.04 |
| GO:1900101 | regulation of endoplasmic reticulum unfolded protein response | 0.01 | 0.04 |
| GO:0002716 | negative regulation of natural killer cell mediated immunity | 0.01 | 0.04 |
| GO:0015919 | peroxisomal membrane transport | 0.01 | 0.04 |
| GO:0039535 | regulation of RIG-I signaling pathway | 0.01 | 0.04 |
| GO:0042454 | ribonucleoside catabolic process | 0.01 | 0.04 |
| GO:0043373 | CD4-positive, alpha-beta T cell lineage commitment | 0.01 | 0.04 |
| GO:0045591 | positive regulation of regulatory T cell differentiation | 0.01 | 0.04 |
| GO:0046485 | ether lipid metabolic process | 0.01 | 0.04 |
| GO:0046629 | gamma-delta T cell activation | 0.01 | 0.04 |
| GO:0046653 | tetrahydrofolate metabolic process | 0.01 | 0.04 |
| GO:0061484 | hematopoietic stem cell homeostasis | 0.01 | 0.04 |
| GO:1904263 | positive regulation of TORC1 signaling | 0.01 | 0.04 |
| GO:1904292 | regulation of ERAD pathway | 0.01 | 0.04 |
| GO:0007043 | cell-cell junction assembly | 0.01 | 0.04 |
| GO:0017015 | regulation of transforming growth factor beta receptor signaling pathway | 0.01 | 0.04 |
| GO:0032496 | response to lipopolysaccharide | 0.01 | 0.04 |
| GO:0045453 | bone resorption | 0.01 | 0.04 |
| GO:0090068 | positive regulation of cell cycle process | 0.01 | 0.04 |
| GO:0006654 | phosphatidic acid biosynthetic process | 0.01 | 0.04 |
| GO:0009112 | nucleobase metabolic process | 0.01 | 0.04 |
| GO:0018345 | protein palmitoylation | 0.01 | 0.04 |
| GO:0043552 | positive regulation of phosphatidylinositol 3-kinase activity | 0.01 | 0.04 |
| GO:0045648 | positive regulation of erythrocyte differentiation | 0.01 | 0.04 |
| GO:1903715 | regulation of aerobic respiration | 0.01 | 0.04 |
| GO:0033866 | nucleoside bisphosphate biosynthetic process | 0.01 | 0.04 |
| GO:0034030 | ribonucleoside bisphosphate biosynthetic process | 0.01 | 0.04 |
| GO:0034033 | purine nucleoside bisphosphate biosynthetic process | 0.01 | 0.04 |
| GO:0036293 | response to decreased oxygen levels | 0.01 | 0.04 |
| GO:0045814 | negative regulation of gene expression, epigenetic | 0.01 | 0.04 |
| GO:0097530 | granulocyte migration | 0.01 | 0.04 |
| GO:0030219 | megakaryocyte differentiation | 0.01 | 0.04 |
| GO:0051302 | regulation of cell division | 0.01 | 0.04 |
| GO:0007031 | peroxisome organization | 0.01 | 0.04 |
| GO:0007431 | salivary gland development | 0.01 | 0.04 |
| GO:0039694 | viral RNA genome replication | 0.01 | 0.04 |
| GO:0043368 | positive T cell selection | 0.01 | 0.04 |
| GO:0045738 | negative regulation of DNA repair | 0.01 | 0.04 |
| GO:0043297 | apical junction assembly | 0.01 | 0.04 |
| GO:0032465 | regulation of cytokinesis | 0.01 | 0.04 |
| GO:0090398 | cellular senescence | 0.01 | 0.04 |
| GO:0051567 | histone H3-K9 methylation | 0.01 | 0.04 |
| GO:0071470 | cellular response to osmotic stress | 0.01 | 0.04 |
| GO:0071634 | regulation of transforming growth factor beta production | 0.01 | 0.04 |
| GO:2000516 | positive regulation of CD4-positive, alpha-beta T cell activation | 0.01 | 0.04 |
| GO:0002639 | positive regulation of immunoglobulin production | 0.01 | 0.04 |
| GO:0009409 | response to cold | 0.01 | 0.04 |
| GO:0002688 | regulation of leukocyte chemotaxis | 0.01 | 0.04 |
| GO:1903828 | negative regulation of protein localization | 0.01 | 0.04 |
| GO:0010543 | regulation of platelet activation | 0.01 | 0.04 |
| GO:0035272 | exocrine system development | 0.01 | 0.04 |
| GO:0031670 | cellular response to nutrient | 0.01 | 0.04 |
| GO:0032735 | positive regulation of interleukin-12 production | 0.01 | 0.04 |
| GO:0071763 | nuclear membrane organization | 0.01 | 0.04 |
| GO:0090503 | RNA phosphodiester bond hydrolysis, exonucleolytic | 0.01 | 0.04 |
| GO:2000249 | regulation of actin cytoskeleton reorganization | 0.01 | 0.04 |
| GO:0006195 | purine nucleotide catabolic process | 0.01 | 0.04 |
| GO:0010171 | body morphogenesis | 0.01 | 0.04 |
| GO:0047496 | vesicle transport along microtubule | 0.01 | 0.04 |
| GO:0048538 | thymus development | 0.01 | 0.04 |
| GO:1902622 | regulation of neutrophil migration | 0.01 | 0.04 |
| GO:1990090 | cellular response to nerve growth factor stimulus | 0.01 | 0.04 |
| GO:0050688 | regulation of defense response to virus | 0.01 | 0.04 |
| GO:0006457 | protein folding | 0.01 | 0.04 |
| GO:0045048 | protein insertion into ER membrane | 0.01 | 0.04 |
| GO:0045943 | positive regulation of transcription by RNA polymerase I | 0.01 | 0.04 |
| GO:0046599 | regulation of centriole replication | 0.01 | 0.04 |
| GO:0072576 | liver morphogenesis | 0.01 | 0.04 |
| GO:0048675 | axon extension | 0.01 | 0.04 |
| GO:0051568 | histone H3-K4 methylation | 0.01 | 0.04 |
| GO:0002062 | chondrocyte differentiation | 0.01 | 0.04 |
| GO:0043547 | positive regulation of GTPase activity | 0.01 | 0.04 |
| GO:0002833 | positive regulation of response to biotic stimulus | 0.01 | 0.04 |
| GO:0002399 | MHC class II protein complex assembly | 0.01 | 0.04 |
| GO:0002503 | peptide antigen assembly with MHC class II protein complex | 0.01 | 0.04 |
| GO:0009163 | nucleoside biosynthetic process | 0.01 | 0.04 |
| GO:0009200 | deoxyribonucleoside triphosphate metabolic process | 0.01 | 0.04 |
| GO:0010715 | regulation of extracellular matrix disassembly | 0.01 | 0.04 |
| GO:0030033 | microvillus assembly | 0.01 | 0.04 |
| GO:0034404 | nucleobase-containing small molecule biosynthetic process | 0.01 | 0.04 |
| GO:0035067 | negative regulation of histone acetylation | 0.01 | 0.04 |
| GO:0035970 | peptidyl-threonine dephosphorylation | 0.01 | 0.04 |
| GO:0044804 | autophagy of nucleus | 0.01 | 0.04 |
| GO:0045651 | positive regulation of macrophage differentiation | 0.01 | 0.04 |
| GO:0080009 | mRNA methylation | 0.01 | 0.04 |
| GO:1902285 | semaphorin-plexin signaling pathway involved in neuron projection guidance | 0.01 | 0.04 |
| GO:0098586 | cellular response to virus | 0.01 | 0.04 |
| GO:0001659 | temperature homeostasis | 0.01 | 0.04 |
| GO:0048736 | appendage development | 0.01 | 0.04 |
| GO:0060173 | limb development | 0.01 | 0.04 |
| GO:0008589 | regulation of smoothened signaling pathway | 0.01 | 0.04 |
| GO:2001021 | negative regulation of response to DNA damage stimulus | 0.01 | 0.04 |
| GO:0031343 | positive regulation of cell killing | 0.01 | 0.04 |
| GO:0033522 | histone H2A ubiquitination | 0.01 | 0.04 |
| GO:0033622 | integrin activation | 0.01 | 0.04 |
| GO:0051894 | positive regulation of focal adhesion assembly | 0.01 | 0.04 |
| GO:0045185 | maintenance of protein location | 0.01 | 0.04 |
| GO:0002237 | response to molecule of bacterial origin | 0.01 | 0.04 |
| GO:0032731 | positive regulation of interleukin-1 beta production | 0.01 | 0.04 |
| GO:0032757 | positive regulation of interleukin-8 production | 0.01 | 0.04 |
| GO:0070828 | heterochromatin organization | 0.01 | 0.05 |
| GO:0010675 | regulation of cellular carbohydrate metabolic process | 0.01 | 0.05 |
| GO:0010720 | positive regulation of cell development | 0.01 | 0.05 |
| GO:0060541 | respiratory system development | 0.01 | 0.05 |
| GO:0071383 | cellular response to steroid hormone stimulus | 0.01 | 0.05 |
| GO:0002328 | pro-B cell differentiation | 0.01 | 0.05 |
| GO:0006047 | UDP-N-acetylglucosamine metabolic process | 0.01 | 0.05 |
| GO:0033540 | fatty acid beta-oxidation using acyl-CoA oxidase | 0.01 | 0.05 |
| GO:0034135 | regulation of toll-like receptor 2 signaling pathway | 0.01 | 0.05 |
| GO:0042451 | purine nucleoside biosynthetic process | 0.01 | 0.05 |
| GO:0042455 | ribonucleoside biosynthetic process | 0.01 | 0.05 |
| GO:0046051 | UTP metabolic process | 0.01 | 0.05 |
| GO:0046129 | purine ribonucleoside biosynthetic process | 0.01 | 0.05 |
| GO:0051639 | actin filament network formation | 0.01 | 0.05 |
| GO:0097201 | negative regulation of transcription from RNA polymerase II promoter in response to stress | 0.01 | 0.05 |
| GO:1902855 | regulation of non-motile cilium assembly | 0.01 | 0.05 |
| GO:1905214 | regulation of RNA binding | 0.01 | 0.05 |
| GO:0046031 | ADP metabolic process | 0.01 | 0.05 |
| GO:0006956 | complement activation | 0.01 | 0.05 |
| GO:0002474 | antigen processing and presentation of peptide antigen via MHC class I | 0.02 | 0.05 |
| GO:0010575 | positive regulation of vascular endothelial growth factor production | 0.02 | 0.05 |
| GO:0017145 | stem cell division | 0.02 | 0.05 |
| GO:0035521 | monoubiquitinated histone deubiquitination | 0.02 | 0.05 |
| GO:0035522 | monoubiquitinated histone H2A deubiquitination | 0.02 | 0.05 |
| GO:1902175 | regulation of oxidative stress-induced intrinsic apoptotic signaling pathway | 0.02 | 0.05 |
| GO:0055076 | transition metal ion homeostasis | 0.02 | 0.05 |
| GO:0051438 | regulation of ubiquitin-protein transferase activity | 0.02 | 0.05 |
| GO:0060337 | type I interferon signaling pathway | 0.02 | 0.05 |
| GO:0001890 | placenta development | 0.02 | 0.05 |
| GO:0071322 | cellular response to carbohydrate stimulus | 0.02 | 0.05 |
| GO:0030100 | regulation of endocytosis | 0.02 | 0.05 |
| GO:0030307 | positive regulation of cell growth | 0.02 | 0.05 |
| GO:0006413 | translational initiation | 0.02 | 0.05 |
| GO:0044843 | cell cycle G1/S phase transition | 0.02 | 0.05 |
| GO:0048260 | positive regulation of receptor-mediated endocytosis | 0.02 | 0.05 |
| GO:0009408 | response to heat | 0.02 | 0.05 |
| GO:0002063 | chondrocyte development | 0.02 | 0.05 |
| GO:0002828 | regulation of type 2 immune response | 0.02 | 0.05 |
| GO:0006221 | pyrimidine nucleotide biosynthetic process | 0.02 | 0.05 |
| GO:0010669 | epithelial structure maintenance | 0.02 | 0.05 |
| GO:0036297 | interstrand cross-link repair | 0.02 | 0.05 |
| GO:0043304 | regulation of mast cell degranulation | 0.02 | 0.05 |
| GO:0051085 | chaperone cofactor-dependent protein refolding | 0.02 | 0.05 |
| GO:0071711 | basement membrane organization | 0.02 | 0.05 |
| GO:0090114 | COPII-coated vesicle budding | 0.02 | 0.05 |
| GO:0060560 | developmental growth involved in morphogenesis | 0.02 | 0.05 |
| GO:0032479 | regulation of type I interferon production | 0.02 | 0.05 |
| GO:0032606 | type I interferon production | 0.02 | 0.05 |
| GO:0001666 | response to hypoxia | 0.02 | 0.05 |
| GO:1903053 | regulation of extracellular matrix organization | 0.02 | 0.05 |
| GO:2000107 | negative regulation of leukocyte apoptotic process | 0.02 | 0.05 |
| GO:0034764 | positive regulation of transmembrane transport | 0.02 | 0.05 |
| GO:0009743 | response to carbohydrate | 0.02 | 0.05 |
| GO:0010824 | regulation of centrosome duplication | 0.02 | 0.05 |
| GO:2000144 | positive regulation of DNA-templated transcription, initiation | 0.02 | 0.05 |
| GO:0010631 | epithelial cell migration | 0.02 | 0.05 |
| GO:0010762 | regulation of fibroblast migration | 0.02 | 0.05 |
| GO:0034110 | regulation of homotypic cell-cell adhesion | 0.02 | 0.05 |
| GO:0042558 | pteridine-containing compound metabolic process | 0.02 | 0.05 |
| GO:0045446 | endothelial cell differentiation | 0.02 | 0.05 |
| GO:0000028 | ribosomal small subunit assembly | 0.02 | 0.05 |
| GO:0000291 | nuclear-transcribed mRNA catabolic process, exonucleolytic | 0.02 | 0.05 |
| GO:0006490 | oligosaccharide-lipid intermediate biosynthetic process | 0.02 | 0.05 |
| GO:0030050 | vesicle transport along actin filament | 0.02 | 0.05 |
| GO:0034501 | protein localization to kinetochore | 0.02 | 0.05 |
| GO:0045618 | positive regulation of keratinocyte differentiation | 0.02 | 0.05 |
| GO:0045953 | negative regulation of natural killer cell mediated cytotoxicity | 0.02 | 0.05 |
| GO:0051900 | regulation of mitochondrial depolarization | 0.02 | 0.05 |
| GO:0071545 | inositol phosphate catabolic process | 0.02 | 0.05 |
| GO:0090043 | regulation of tubulin deacetylation | 0.02 | 0.05 |
| GO:1902170 | cellular response to reactive nitrogen species | 0.02 | 0.05 |
| GO:1903083 | protein localization to condensed chromosome | 0.02 | 0.05 |
| GO:1901607 | alpha-amino acid biosynthetic process | 0.02 | 0.05 |
| GO:0062208 | positive regulation of pattern recognition receptor signaling pathway | 0.02 | 0.05 |
| GO:1903214 | regulation of protein targeting to mitochondrion | 0.02 | 0.05 |
| GO:0045191 | regulation of isotype switching | 0.02 | 0.05 |
| GO:0042119 | neutrophil activation | 0.02 | 0.05 |
| GO:0060261 | positive regulation of transcription initiation from RNA polymerase II promoter | 0.02 | 0.05 |
| GO:1902110 | positive regulation of mitochondrial membrane permeability involved in apoptotic process | 0.02 | 0.05 |
| GO:2000008 | regulation of protein localization to cell surface | 0.02 | 0.05 |
| GO:0006090 | pyruvate metabolic process | 0.02 | 0.05 |
| GO:0030593 | neutrophil chemotaxis | 0.02 | 0.05 |
| GO:1903844 | regulation of cellular response to transforming growth factor beta stimulus | 0.02 | 0.05 |
| GO:0005925 | focal adhesion | 0.00 | 0.00 |
| GO:0030055 | cell-substrate junction | 0.00 | 0.00 |
| GO:0005759 | mitochondrial matrix | 0.00 | 0.00 |
| GO:0031252 | cell leading edge | 0.00 | 0.00 |
| GO:0005819 | spindle | 0.00 | 0.00 |
| GO:0005769 | early endosome | 0.00 | 0.00 |
| GO:0030139 | endocytic vesicle | 0.00 | 0.00 |
| GO:0030027 | lamellipodium | 0.00 | 0.00 |
| GO:0001726 | ruffle | 0.00 | 0.00 |
| GO:0031300 | intrinsic component of organelle membrane | 0.00 | 0.00 |
| GO:0016607 | nuclear speck | 0.00 | 0.00 |
| GO:0005774 | vacuolar membrane | 0.00 | 0.00 |
| GO:0055037 | recycling endosome | 0.00 | 0.00 |
| GO:0031301 | integral component of organelle membrane | 0.00 | 0.00 |
| GO:0005765 | lysosomal membrane | 0.00 | 0.00 |
| GO:0098852 | lytic vacuole membrane | 0.00 | 0.00 |
| GO:0030135 | coated vesicle | 0.00 | 0.00 |
| GO:0015629 | actin cytoskeleton | 0.00 | 0.00 |
| GO:0000922 | spindle pole | 0.00 | 0.00 |
| GO:0005743 | mitochondrial inner membrane | 0.00 | 0.00 |
| GO:0030496 | midbody | 0.00 | 0.00 |
| GO:0005635 | nuclear envelope | 0.00 | 0.00 |
| GO:0005681 | spliceosomal complex | 0.00 | 0.00 |
| GO:0032587 | ruffle membrane | 0.00 | 0.00 |
| GO:0005770 | late endosome | 0.00 | 0.00 |
| GO:0044391 | ribosomal subunit | 0.00 | 0.00 |
| GO:0045335 | phagocytic vesicle | 0.00 | 0.00 |
| GO:0031227 | intrinsic component of endoplasmic reticulum membrane | 0.00 | 0.00 |
| GO:0030176 | integral component of endoplasmic reticulum membrane | 0.00 | 0.00 |
| GO:0000151 | ubiquitin ligase complex | 0.00 | 0.00 |
| GO:0005938 | cell cortex | 0.00 | 0.00 |
| GO:0031901 | early endosome membrane | 0.00 | 0.00 |
| GO:0098798 | mitochondrial protein-containing complex | 0.00 | 0.00 |
| GO:0005874 | microtubule | 0.00 | 0.00 |
| GO:0098791 | Golgi apparatus subcompartment | 0.00 | 0.00 |
| GO:0005814 | centriole | 0.00 | 0.00 |
| GO:0140534 | endoplasmic reticulum protein-containing complex | 0.00 | 0.00 |
| GO:0005802 | trans-Golgi network | 0.00 | 0.00 |
| GO:0098687 | chromosomal region | 0.00 | 0.00 |
| GO:0035770 | ribonucleoprotein granule | 0.00 | 0.00 |
| GO:0030136 | clathrin-coated vesicle | 0.00 | 0.00 |
| GO:0055038 | recycling endosome membrane | 0.00 | 0.00 |
| GO:0031965 | nuclear membrane | 0.00 | 0.00 |
| GO:0072686 | mitotic spindle | 0.00 | 0.00 |
| GO:0015934 | large ribosomal subunit | 0.00 | 0.00 |
| GO:1905368 | peptidase complex | 0.00 | 0.00 |
| GO:0090575 | RNA polymerase II transcription regulator complex | 0.00 | 0.00 |
| GO:0005667 | transcription regulator complex | 0.00 | 0.00 |
| GO:0005884 | actin filament | 0.00 | 0.00 |
| GO:0005778 | peroxisomal membrane | 0.00 | 0.00 |
| GO:0031903 | microbody membrane | 0.00 | 0.00 |
| GO:0005684 | U2-type spliceosomal complex | 0.00 | 0.00 |
| GO:0034774 | secretory granule lumen | 0.00 | 0.00 |
| GO:0000313 | organellar ribosome | 0.00 | 0.00 |
| GO:0005761 | mitochondrial ribosome | 0.00 | 0.00 |
| GO:0030662 | coated vesicle membrane | 0.00 | 0.00 |
| GO:0030117 | membrane coat | 0.00 | 0.00 |
| GO:0048475 | coated membrane | 0.00 | 0.00 |
| GO:0005840 | ribosome | 0.00 | 0.00 |
| GO:0034451 | centriolar satellite | 0.00 | 0.00 |
| GO:0036464 | cytoplasmic ribonucleoprotein granule | 0.00 | 0.00 |
| GO:0060205 | cytoplasmic vesicle lumen | 0.00 | 0.00 |
| GO:0031983 | vesicle lumen | 0.00 | 0.00 |
| GO:0030666 | endocytic vesicle membrane | 0.00 | 0.00 |
| GO:0031253 | cell projection membrane | 0.00 | 0.00 |
| GO:0071011 | precatalytic spliceosome | 0.00 | 0.00 |
| GO:0005657 | replication fork | 0.00 | 0.00 |
| GO:0030134 | COPII-coated ER to Golgi transport vesicle | 0.00 | 0.00 |
| GO:0005777 | peroxisome | 0.00 | 0.00 |
| GO:0042579 | microbody | 0.00 | 0.00 |
| GO:0030670 | phagocytic vesicle membrane | 0.00 | 0.00 |
| GO:1904813 | ficolin-1-rich granule lumen | 0.00 | 0.00 |
| GO:1905369 | endopeptidase complex | 0.00 | 0.00 |
| GO:0005881 | cytoplasmic microtubule | 0.00 | 0.00 |
| GO:0000315 | organellar large ribosomal subunit | 0.00 | 0.00 |
| GO:0005762 | mitochondrial large ribosomal subunit | 0.00 | 0.00 |
| GO:0032592 | integral component of mitochondrial membrane | 0.00 | 0.00 |
| GO:0071005 | U2-type precatalytic spliceosome | 0.00 | 0.00 |
| GO:0061695 | transferase complex, transferring phosphorus-containing groups | 0.00 | 0.00 |
| GO:0036064 | ciliary basal body | 0.00 | 0.00 |
| GO:0005798 | Golgi-associated vesicle | 0.00 | 0.00 |
| GO:0005793 | endoplasmic reticulum-Golgi intermediate compartment | 0.00 | 0.00 |
| GO:0031902 | late endosome membrane | 0.00 | 0.00 |
| GO:0001650 | fibrillar center | 0.00 | 0.00 |
| GO:0098573 | intrinsic component of mitochondrial membrane | 0.00 | 0.00 |
| GO:0031256 | leading edge membrane | 0.00 | 0.00 |
| GO:0042581 | specific granule | 0.00 | 0.00 |
| GO:0005911 | cell-cell junction | 0.00 | 0.00 |
| GO:0019867 | outer membrane | 0.00 | 0.00 |
| GO:1990204 | oxidoreductase complex | 0.00 | 0.00 |
| GO:0005741 | mitochondrial outer membrane | 0.00 | 0.00 |
| GO:0000792 | heterochromatin | 0.00 | 0.00 |
| GO:0019814 | immunoglobulin complex | 0.00 | 0.00 |
| GO:0031968 | organelle outer membrane | 0.00 | 0.00 |
| GO:0032432 | actin filament bundle | 0.00 | 0.00 |
| GO:0030140 | trans-Golgi network transport vesicle | 0.00 | 0.00 |
| GO:0071782 | endoplasmic reticulum tubular network | 0.00 | 0.00 |
| GO:0042470 | melanosome | 0.00 | 0.00 |
| GO:0048770 | pigment granule | 0.00 | 0.00 |
| GO:0097542 | ciliary tip | 0.00 | 0.00 |
| GO:0042641 | actomyosin | 0.00 | 0.00 |
| GO:0022626 | cytosolic ribosome | 0.00 | 0.00 |
| GO:0005905 | clathrin-coated pit | 0.00 | 0.00 |
| GO:0030667 | secretory granule membrane | 0.00 | 0.00 |
| GO:0032153 | cell division site | 0.00 | 0.00 |
| GO:0000242 | pericentriolar material | 0.00 | 0.00 |
| GO:0031461 | cullin-RING ubiquitin ligase complex | 0.00 | 0.00 |
| GO:0000776 | kinetochore | 0.00 | 0.00 |
| GO:0071013 | catalytic step 2 spliceosome | 0.00 | 0.00 |
| GO:0097346 | INO80-type complex | 0.00 | 0.00 |
| GO:0000502 | proteasome complex | 0.00 | 0.00 |
| GO:0035579 | specific granule membrane | 0.00 | 0.00 |
| GO:0030660 | Golgi-associated vesicle membrane | 0.00 | 0.00 |
| GO:0000779 | condensed chromosome, centromeric region | 0.00 | 0.00 |
| GO:0000930 | gamma-tubulin complex | 0.00 | 0.00 |
| GO:0030684 | preribosome | 0.00 | 0.00 |
| GO:0044232 | organelle membrane contact site | 0.00 | 0.00 |
| GO:0005766 | primary lysosome | 0.00 | 0.00 |
| GO:0042582 | azurophil granule | 0.00 | 0.00 |
| GO:0098857 | membrane microdomain | 0.00 | 0.00 |
| GO:0030133 | transport vesicle | 0.00 | 0.00 |
| GO:0098858 | actin-based cell projection | 0.00 | 0.00 |
| GO:0099023 | vesicle tethering complex | 0.00 | 0.00 |
| GO:0042611 | MHC protein complex | 0.00 | 0.00 |
| GO:0009295 | nucleoid | 0.00 | 0.00 |
| GO:0042645 | mitochondrial nucleoid | 0.00 | 0.00 |
| GO:0000781 | chromosome, telomeric region | 0.00 | 0.00 |
| GO:0002102 | podosome | 0.00 | 0.00 |
| GO:0071556 | integral component of lumenal side of endoplasmic reticulum membrane | 0.00 | 0.00 |
| GO:0098553 | lumenal side of endoplasmic reticulum membrane | 0.00 | 0.00 |
| GO:0005876 | spindle microtubule | 0.00 | 0.00 |
| GO:0045121 | membrane raft | 0.00 | 0.00 |
| GO:0005775 | vacuolar lumen | 0.00 | 0.00 |
| GO:0000775 | chromosome, centromeric region | 0.00 | 0.00 |
| GO:0101002 | ficolin-1-rich granule | 0.00 | 0.00 |
| GO:0005779 | integral component of peroxisomal membrane | 0.00 | 0.00 |
| GO:0031231 | intrinsic component of peroxisomal membrane | 0.00 | 0.00 |
| GO:0030864 | cortical actin cytoskeleton | 0.00 | 0.00 |
| GO:1904949 | ATPase complex | 0.00 | 0.00 |
| GO:0005776 | autophagosome | 0.00 | 0.00 |
| GO:0090734 | site of DNA damage | 0.00 | 0.00 |
| GO:0032588 | trans-Golgi network membrane | 0.00 | 0.00 |
| GO:0001725 | stress fiber | 0.00 | 0.00 |
| GO:0015935 | small ribosomal subunit | 0.00 | 0.00 |
| GO:0097517 | contractile actin filament bundle | 0.00 | 0.00 |
| GO:0016605 | PML body | 0.00 | 0.00 |
| GO:0030175 | filopodium | 0.00 | 0.00 |
| GO:0000932 | P-body | 0.00 | 0.00 |
| GO:0090543 | Flemming body | 0.00 | 0.00 |
| GO:0005788 | endoplasmic reticulum lumen | 0.00 | 0.00 |
| GO:0005844 | polysome | 0.00 | 0.00 |
| GO:0030120 | vesicle coat | 0.00 | 0.00 |
| GO:0000428 | DNA-directed RNA polymerase complex | 0.00 | 0.00 |
| GO:0070603 | SWI/SNF superfamily-type complex | 0.00 | 0.00 |
| GO:0070820 | tertiary granule | 0.00 | 0.00 |
| GO:0035861 | site of double-strand break | 0.00 | 0.00 |
| GO:0055029 | nuclear DNA-directed RNA polymerase complex | 0.00 | 0.00 |
| GO:0010494 | cytoplasmic stress granule | 0.00 | 0.00 |
| GO:0045334 | clathrin-coated endocytic vesicle | 0.00 | 0.00 |
| GO:0001772 | immunological synapse | 0.00 | 0.00 |
| GO:0031304 | intrinsic component of mitochondrial inner membrane | 0.00 | 0.00 |
| GO:0098644 | complex of collagen trimers | 0.00 | 0.00 |
| GO:0098562 | cytoplasmic side of membrane | 0.00 | 0.00 |
| GO:0030665 | clathrin-coated vesicle membrane | 0.00 | 0.00 |
| GO:0030880 | RNA polymerase complex | 0.00 | 0.00 |
| GO:0008287 | protein serine/threonine phosphatase complex | 0.00 | 0.00 |
| GO:1903293 | phosphatase complex | 0.00 | 0.00 |
| GO:0000228 | nuclear chromosome | 0.00 | 0.00 |
| GO:0031305 | integral component of mitochondrial inner membrane | 0.00 | 0.00 |
| GO:0098636 | protein complex involved in cell adhesion | 0.00 | 0.00 |
| GO:0012507 | ER to Golgi transport vesicle membrane | 0.00 | 0.00 |
| GO:0097431 | mitotic spindle pole | 0.00 | 0.00 |
| GO:0016234 | inclusion body | 0.00 | 0.00 |
| GO:0032154 | cleavage furrow | 0.00 | 0.00 |
| GO:0031248 | protein acetyltransferase complex | 0.00 | 0.00 |
| GO:0043202 | lysosomal lumen | 0.00 | 0.00 |
| GO:1902493 | acetyltransferase complex | 0.00 | 0.00 |
| GO:0098576 | lumenal side of membrane | 0.00 | 0.01 |
| GO:0009897 | external side of plasma membrane | 0.00 | 0.01 |
| GO:0000123 | histone acetyltransferase complex | 0.00 | 0.01 |
| GO:0008305 | integrin complex | 0.00 | 0.01 |
| GO:0044233 | mitochondria-associated endoplasmic reticulum membrane | 0.00 | 0.01 |
| GO:0034399 | nuclear periphery | 0.00 | 0.01 |
| GO:0030863 | cortical cytoskeleton | 0.00 | 0.01 |
| GO:0017119 | Golgi transport complex | 0.00 | 0.01 |
| GO:0016363 | nuclear matrix | 0.00 | 0.01 |
| GO:0042571 | immunoglobulin complex, circulating | 0.00 | 0.01 |
| GO:0022624 | proteasome accessory complex | 0.00 | 0.01 |
| GO:0030119 | AP-type membrane coat adaptor complex | 0.00 | 0.01 |
| GO:0031091 | platelet alpha granule | 0.00 | 0.01 |
| GO:0012510 | trans-Golgi network transport vesicle membrane | 0.00 | 0.01 |
| GO:0031011 | Ino80 complex | 0.00 | 0.01 |
| GO:0009898 | cytoplasmic side of plasma membrane | 0.00 | 0.01 |
| GO:0000307 | cyclin-dependent protein kinase holoenzyme complex | 0.00 | 0.01 |
| GO:0016591 | RNA polymerase II, holoenzyme | 0.00 | 0.01 |
| GO:0031234 | extrinsic component of cytoplasmic side of plasma membrane | 0.00 | 0.01 |
| GO:1902554 | serine/threonine protein kinase complex | 0.00 | 0.01 |
| GO:0022625 | cytosolic large ribosomal subunit | 0.00 | 0.01 |
| GO:0030681 | multimeric ribonuclease P complex | 0.00 | 0.01 |
| GO:1902911 | protein kinase complex | 0.01 | 0.01 |
| GO:0000118 | histone deacetylase complex | 0.01 | 0.01 |
| GO:0043296 | apical junction complex | 0.01 | 0.01 |
| GO:0008023 | transcription elongation factor complex | 0.01 | 0.01 |
| GO:0070993 | translation preinitiation complex | 0.01 | 0.01 |
| GO:0016235 | aggresome | 0.01 | 0.01 |
| GO:0005811 | lipid droplet | 0.01 | 0.01 |
| GO:0033116 | endoplasmic reticulum-Golgi intermediate compartment membrane | 0.01 | 0.02 |
| GO:0019005 | SCF ubiquitin ligase complex | 0.01 | 0.02 |
| GO:1904115 | axon cytoplasm | 0.01 | 0.02 |
| GO:0005903 | brush border | 0.01 | 0.02 |
| GO:0016282 | eukaryotic 43S preinitiation complex | 0.01 | 0.02 |
| GO:0042599 | lamellar body | 0.01 | 0.02 |
| GO:0042613 | MHC class II protein complex | 0.01 | 0.02 |
| GO:0030990 | intraciliary transport particle | 0.01 | 0.02 |
| GO:0005912 | adherens junction | 0.01 | 0.02 |
| GO:0022627 | cytosolic small ribosomal subunit | 0.01 | 0.02 |
| GO:0000812 | Swr1 complex | 0.01 | 0.02 |
| GO:0030130 | clathrin coat of trans-Golgi network vesicle | 0.01 | 0.02 |
| GO:1990531 | phospholipid-translocating ATPase complex | 0.01 | 0.02 |
| GO:1904724 | tertiary granule lumen | 0.01 | 0.02 |
| GO:0034708 | methyltransferase complex | 0.01 | 0.02 |
| GO:0030669 | clathrin-coated endocytic vesicle membrane | 0.01 | 0.02 |
| GO:0120111 | neuron projection cytoplasm | 0.01 | 0.02 |
| GO:0035869 | ciliary transition zone | 0.01 | 0.02 |
| GO:0033178 | proton-transporting two-sector ATPase complex, catalytic domain | 0.01 | 0.02 |
| GO:1990752 | microtubule end | 0.01 | 0.03 |
| GO:0031463 | Cul3-RING ubiquitin ligase complex | 0.01 | 0.03 |
| GO:0030118 | clathrin coat | 0.01 | 0.03 |
| GO:0032040 | small-subunit processome | 0.01 | 0.03 |
| GO:0035578 | azurophil granule lumen | 0.01 | 0.03 |
| GO:0000152 | nuclear ubiquitin ligase complex | 0.01 | 0.03 |
| GO:0030687 | preribosome, large subunit precursor | 0.01 | 0.03 |
| GO:0035371 | microtubule plus-end | 0.01 | 0.03 |
| GO:0030427 | site of polarized growth | 0.01 | 0.03 |
| GO:0019898 | extrinsic component of membrane | 0.01 | 0.03 |
| GO:0000314 | organellar small ribosomal subunit | 0.01 | 0.03 |
| GO:0005763 | mitochondrial small ribosomal subunit | 0.01 | 0.03 |
| GO:0046930 | pore complex | 0.01 | 0.03 |
| GO:0000791 | euchromatin | 0.01 | 0.03 |
| GO:0030173 | integral component of Golgi membrane | 0.01 | 0.03 |
| GO:0005583 | fibrillar collagen trimer | 0.01 | 0.03 |
| GO:0005797 | Golgi medial cisterna | 0.01 | 0.03 |
| GO:0098643 | banded collagen fibril | 0.01 | 0.03 |
| GO:0005689 | U12-type spliceosomal complex | 0.02 | 0.03 |
| GO:0005902 | microvillus | 0.02 | 0.03 |
| GO:0098800 | inner mitochondrial membrane protein complex | 0.02 | 0.03 |
| GO:0000793 | condensed chromosome | 0.02 | 0.03 |
| GO:0000178 | exosome (RNase complex) | 0.02 | 0.03 |
| GO:0005838 | proteasome regulatory particle | 0.02 | 0.03 |
| GO:0031093 | platelet alpha granule lumen | 0.02 | 0.03 |
| GO:0031228 | intrinsic component of Golgi membrane | 0.02 | 0.03 |
| GO:0030137 | COPI-coated vesicle | 0.02 | 0.04 |
| GO:0031258 | lamellipodium membrane | 0.02 | 0.04 |
| GO:0045171 | intercellular bridge | 0.02 | 0.04 |
| GO:0035577 | azurophil granule membrane | 0.02 | 0.04 |
| GO:0033290 | eukaryotic 48S preinitiation complex | 0.02 | 0.04 |
| GO:0005871 | kinesin complex | 0.02 | 0.04 |
| GO:0005963 | magnesium-dependent protein serine/threonine phosphatase complex | 0.02 | 0.04 |
| GO:0036019 | endolysosome | 0.02 | 0.04 |
| GO:0043596 | nuclear replication fork | 0.02 | 0.04 |
| GO:0030426 | growth cone | 0.02 | 0.04 |
| GO:0005795 | Golgi stack | 0.02 | 0.04 |
| GO:0002178 | palmitoyltransferase complex | 0.02 | 0.04 |
| GO:0017101 | aminoacyl-tRNA synthetase multienzyme complex | 0.02 | 0.04 |
| GO:0034663 | endoplasmic reticulum chaperone complex | 0.02 | 0.04 |
| GO:0072562 | blood microparticle | 0.02 | 0.04 |
| GO:0005791 | rough endoplasmic reticulum | 0.03 | 0.05 |
| GO:0045296 | cadherin binding | 0.00 | 0.00 |
| GO:0003712 | transcription coregulator activity | 0.00 | 0.00 |
| GO:0004712 | protein serine/threonine/tyrosine kinase activity | 0.00 | 0.00 |
| GO:0140297 | DNA-binding transcription factor binding | 0.00 | 0.00 |
| GO:0106310 | protein serine kinase activity | 0.00 | 0.00 |
| GO:0005085 | guanyl-nucleotide exchange factor activity | 0.00 | 0.00 |
| GO:0044389 | ubiquitin-like protein ligase binding | 0.00 | 0.00 |
| GO:0004674 | protein serine/threonine kinase activity | 0.00 | 0.00 |
| GO:0140098 | catalytic activity, acting on RNA | 0.00 | 0.00 |
| GO:0031625 | ubiquitin protein ligase binding | 0.00 | 0.00 |
| GO:0003713 | transcription coactivator activity | 0.00 | 0.00 |
| GO:0051020 | GTPase binding | 0.00 | 0.00 |
| GO:0031267 | small GTPase binding | 0.00 | 0.00 |
| GO:0035091 | phosphatidylinositol binding | 0.00 | 0.00 |
| GO:0004860 | protein kinase inhibitor activity | 0.00 | 0.00 |
| GO:0019210 | kinase inhibitor activity | 0.00 | 0.00 |
| GO:0061629 | RNA polymerase II-specific DNA-binding transcription factor binding | 0.00 | 0.00 |
| GO:0005543 | phospholipid binding | 0.00 | 0.00 |
| GO:0016887 | ATP hydrolysis activity | 0.00 | 0.00 |
| GO:0032182 | ubiquitin-like protein binding | 0.00 | 0.00 |
| GO:0030695 | GTPase regulator activity | 0.00 | 0.00 |
| GO:0060589 | nucleoside-triphosphatase regulator activity | 0.00 | 0.00 |
| GO:0019207 | kinase regulator activity | 0.00 | 0.00 |
| GO:0002020 | protease binding | 0.00 | 0.00 |
| GO:0003779 | actin binding | 0.00 | 0.00 |
| GO:0140101 | catalytic activity, acting on a tRNA | 0.00 | 0.00 |
| GO:0019887 | protein kinase regulator activity | 0.00 | 0.00 |
| GO:0004386 | helicase activity | 0.00 | 0.00 |
| GO:0019902 | phosphatase binding | 0.00 | 0.00 |
| GO:0017124 | SH3 domain binding | 0.00 | 0.00 |
| GO:0005525 | GTP binding | 0.00 | 0.00 |
| GO:0003823 | antigen binding | 0.00 | 0.00 |
| GO:0019001 | guanyl nucleotide binding | 0.00 | 0.00 |
| GO:0032561 | guanyl ribonucleotide binding | 0.00 | 0.00 |
| GO:0008094 | ATP-dependent activity, acting on DNA | 0.00 | 0.00 |
| GO:0051015 | actin filament binding | 0.00 | 0.00 |
| GO:0019787 | ubiquitin-like protein transferase activity | 0.00 | 0.00 |
| GO:0004713 | protein tyrosine kinase activity | 0.00 | 0.00 |
| GO:0008186 | ATP-dependent activity, acting on RNA | 0.00 | 0.00 |
| GO:0043130 | ubiquitin binding | 0.00 | 0.00 |
| GO:0004842 | ubiquitin-protein transferase activity | 0.00 | 0.00 |
| GO:0019903 | protein phosphatase binding | 0.00 | 0.00 |
| GO:0030291 | protein serine/threonine kinase inhibitor activity | 0.00 | 0.00 |
| GO:0002039 | p53 binding | 0.00 | 0.00 |
| GO:0003724 | RNA helicase activity | 0.00 | 0.00 |
| GO:0001221 | transcription coregulator binding | 0.00 | 0.00 |
| GO:0003714 | transcription corepressor activity | 0.00 | 0.00 |
| GO:0048027 | mRNA 5'-UTR binding | 0.00 | 0.00 |
| GO:0008017 | microtubule binding | 0.00 | 0.00 |
| GO:0060090 | molecular adaptor activity | 0.00 | 0.00 |
| GO:0015631 | tubulin binding | 0.00 | 0.00 |
| GO:0008757 | S-adenosylmethionine-dependent methyltransferase activity | 0.00 | 0.00 |
| GO:1901981 | phosphatidylinositol phosphate binding | 0.00 | 0.00 |
| GO:0043022 | ribosome binding | 0.00 | 0.00 |
| GO:0019208 | phosphatase regulator activity | 0.00 | 0.00 |
| GO:0140272 | exogenous protein binding | 0.00 | 0.00 |
| GO:0016922 | nuclear receptor binding | 0.00 | 0.00 |
| GO:0030674 | protein-macromolecule adaptor activity | 0.00 | 0.00 |
| GO:0000287 | magnesium ion binding | 0.00 | 0.00 |
| GO:0140097 | catalytic activity, acting on DNA | 0.00 | 0.00 |
| GO:0001618 | virus receptor activity | 0.00 | 0.00 |
| GO:0001217 | DNA-binding transcription repressor activity | 0.00 | 0.00 |
| GO:0001227 | DNA-binding transcription repressor activity, RNA polymerase II-specific | 0.00 | 0.00 |
| GO:0047485 | protein N-terminus binding | 0.00 | 0.00 |
| GO:0004715 | non-membrane spanning protein tyrosine kinase activity | 0.00 | 0.00 |
| GO:0030374 | nuclear receptor coactivator activity | 0.00 | 0.00 |
| GO:0004527 | exonuclease activity | 0.00 | 0.00 |
| GO:0001222 | transcription corepressor binding | 0.00 | 0.00 |
| GO:0032266 | phosphatidylinositol-3-phosphate binding | 0.00 | 0.00 |
| GO:0003678 | DNA helicase activity | 0.00 | 0.00 |
| GO:0051059 | NF-kappaB binding | 0.00 | 0.00 |
| GO:0003688 | DNA replication origin binding | 0.00 | 0.00 |
| GO:0004722 | protein serine/threonine phosphatase activity | 0.00 | 0.01 |
| GO:0004721 | phosphoprotein phosphatase activity | 0.00 | 0.01 |
| GO:0019888 | protein phosphatase regulator activity | 0.00 | 0.01 |
| GO:0042169 | SH2 domain binding | 0.00 | 0.01 |
| GO:0051117 | ATPase binding | 0.00 | 0.01 |
| GO:0050660 | flavin adenine dinucleotide binding | 0.00 | 0.01 |
| GO:0043021 | ribonucleoprotein complex binding | 0.00 | 0.01 |
| GO:0003924 | GTPase activity | 0.00 | 0.01 |
| GO:0008022 | protein C-terminus binding | 0.00 | 0.01 |
| GO:0061659 | ubiquitin-like protein ligase activity | 0.00 | 0.01 |
| GO:0140035 | ubiquitination-like modification-dependent protein binding | 0.00 | 0.01 |
| GO:0016667 | oxidoreductase activity, acting on a sulfur group of donors | 0.00 | 0.01 |
| GO:0019838 | growth factor binding | 0.00 | 0.01 |
| GO:0005547 | phosphatidylinositol-3,4,5-trisphosphate binding | 0.00 | 0.01 |
| GO:0016776 | phosphotransferase activity, phosphate group as acceptor | 0.00 | 0.01 |
| GO:0031072 | heat shock protein binding | 0.00 | 0.01 |
| GO:0005178 | integrin binding | 0.00 | 0.01 |
| GO:0043236 | laminin binding | 0.00 | 0.01 |
| GO:0016627 | oxidoreductase activity, acting on the CH-CH group of donors | 0.00 | 0.02 |
| GO:0016765 | transferase activity, transferring alkyl or aryl (other than methyl) groups | 0.00 | 0.02 |
| GO:0009982 | pseudouridine synthase activity | 0.00 | 0.02 |
| GO:0016741 | transferase activity, transferring one-carbon groups | 0.00 | 0.02 |
| GO:0061630 | ubiquitin protein ligase activity | 0.00 | 0.02 |
| GO:0043015 | gamma-tubulin binding | 0.00 | 0.02 |
| GO:0016791 | phosphatase activity | 0.00 | 0.02 |
| GO:0042578 | phosphoric ester hydrolase activity | 0.00 | 0.02 |
| GO:0045309 | protein phosphorylated amino acid binding | 0.00 | 0.02 |
| GO:0042393 | histone binding | 0.00 | 0.02 |
| GO:0051536 | iron-sulfur cluster binding | 0.00 | 0.02 |
| GO:0051540 | metal cluster binding | 0.00 | 0.02 |
| GO:0015095 | magnesium ion transmembrane transporter activity | 0.00 | 0.02 |
| GO:0004861 | cyclin-dependent protein serine/threonine kinase inhibitor activity | 0.00 | 0.02 |
| GO:0035325 | Toll-like receptor binding | 0.00 | 0.02 |
| GO:0140036 | ubiquitin-dependent protein binding | 0.00 | 0.02 |
| GO:0005520 | insulin-like growth factor binding | 0.00 | 0.02 |
| GO:0043325 | phosphatidylinositol-3,4-bisphosphate binding | 0.00 | 0.02 |
| GO:0003735 | structural constituent of ribosome | 0.00 | 0.03 |
| GO:0008266 | poly(U) RNA binding | 0.00 | 0.03 |
| GO:0038024 | cargo receptor activity | 0.00 | 0.03 |
| GO:0016504 | peptidase activator activity | 0.00 | 0.03 |
| GO:0051219 | phosphoprotein binding | 0.00 | 0.03 |
| GO:0008170 | N-methyltransferase activity | 0.00 | 0.03 |
| GO:0008168 | methyltransferase activity | 0.00 | 0.03 |
| GO:0016796 | exonuclease activity, active with either ribo- or deoxyribonucleic acids and producing 5'-phosphomonoesters | 0.00 | 0.03 |
| GO:0000900 | mRNA regulatory element binding translation repressor activity | 0.00 | 0.03 |
| GO:0070402 | NADPH binding | 0.00 | 0.03 |
| GO:0008187 | poly-pyrimidine tract binding | 0.00 | 0.03 |
| GO:0008173 | RNA methyltransferase activity | 0.00 | 0.03 |
| GO:0034511 | U3 snoRNA binding | 0.00 | 0.03 |
| GO:0015036 | disulfide oxidoreductase activity | 0.00 | 0.03 |
| GO:0004529 | exodeoxyribonuclease activity | 0.00 | 0.03 |
| GO:0016895 | exodeoxyribonuclease activity, producing 5'-phosphomonoesters | 0.00 | 0.03 |
| GO:0050840 | extracellular matrix binding | 0.00 | 0.03 |
| GO:0004812 | aminoacyl-tRNA ligase activity | 0.01 | 0.04 |
| GO:0016875 | ligase activity, forming carbon-oxygen bonds | 0.01 | 0.04 |
| GO:0051539 | 4 iron, 4 sulfur cluster binding | 0.01 | 0.04 |
| GO:0016874 | ligase activity | 0.01 | 0.04 |
| GO:0004707 | MAP kinase activity | 0.01 | 0.04 |
| GO:1904680 | peptide transmembrane transporter activity | 0.01 | 0.04 |
| GO:0017116 | single-stranded DNA helicase activity | 0.01 | 0.04 |
| GO:0030544 | Hsp70 protein binding | 0.01 | 0.05 |
| GO:0015035 | protein-disulfide reductase activity | 0.01 | 0.05 |
| GO:0004300 | enoyl-CoA hydratase activity | 0.01 | 0.05 |
| GO:0016783 | sulfurtransferase activity | 0.01 | 0.05 |
| GO:0030957 | Tat protein binding | 0.01 | 0.05 |
| GO:0033204 | ribonuclease P RNA binding | 0.01 | 0.05 |
| GO:0042608 | T cell receptor binding | 0.01 | 0.05 |
| GO:0043139 | 5'-3' DNA helicase activity | 0.01 | 0.05 |
| GO:0071723 | lipopeptide binding | 0.01 | 0.05 |
| GO:0016417 | S-acyltransferase activity | 0.01 | 0.05 |
| GO:0071889 | 14-3-3 protein binding | 0.01 | 0.05 |
| GO:0019843 | rRNA binding | 0.01 | 0.05 |
| GO:0016538 | cyclin-dependent protein serine/threonine kinase regulator activity | 0.01 | 0.05 |
| GO:0000049 | tRNA binding | 0.01 | 0.05 |

**Abbreviations**: P-value: Probability; q-value: Adjusted P-value.
