# Supplementary material for: Gefitinib metabolism-related lncRNAs for the prediction of prognosis, tumor microenvironment and drug sensitivity in lung adenocarcinoma
Source: Sci Rep. 2024 May 6;14:10348. doi: 10.1038/s41598-024-61175-3 (PMC11074108; doi:10.1038/s41598-024-61175-3)
Supplement: Supplementary file 21 — Supplementary Table S7. [file 41598_2024_61175_MOESM21_ESM.docx]

**Table S7** All pathways of KEGG enrichment analysis.

| **ID** | **Description** | **P-value** | **q-value** |
| --- | --- | --- | --- |
| hsa05132 | Salmonella infection | 0.00 | 0.00 |
| hsa05166 | Human T-cell leukemia virus 1 infection | 0.00 | 0.00 |
| hsa05131 | Shigellosis | 0.00 | 0.00 |
| hsa05135 | Yersinia infection | 0.00 | 0.00 |
| hsa04933 | AGE-RAGE signaling pathway in diabetic complications | 0.00 | 0.00 |
| hsa04510 | Focal adhesion | 0.00 | 0.00 |
| hsa04144 | Endocytosis | 0.00 | 0.00 |
| hsa04145 | Phagosome | 0.00 | 0.00 |
| hsa05145 | Toxoplasmosis | 0.00 | 0.00 |
| hsa04666 | Fc gamma R-mediated phagocytosis | 0.00 | 0.00 |
| hsa05130 | Pathogenic Escherichia coli infection | 0.00 | 0.00 |
| hsa05169 | Epstein-Barr virus infection | 0.00 | 0.00 |
| hsa04140 | Autophagy - animal | 0.00 | 0.00 |
| hsa05235 | PD-L1 expression and PD-1 checkpoint pathway in cancer | 0.00 | 0.00 |
| hsa04810 | Regulation of actin cytoskeleton | 0.00 | 0.00 |
| hsa03250 | Viral life cycle - HIV-1 | 0.00 | 0.00 |
| hsa04380 | Osteoclast differentiation | 0.00 | 0.00 |
| hsa05203 | Viral carcinogenesis | 0.00 | 0.00 |
| hsa05223 | Non-small cell lung cancer | 0.00 | 0.00 |
| hsa05210 | Colorectal cancer | 0.00 | 0.00 |
| hsa04520 | Adherens junction | 0.00 | 0.00 |
| hsa04071 | Sphingolipid signaling pathway | 0.00 | 0.00 |
| hsa04658 | Th1 and Th2 cell differentiation | 0.00 | 0.00 |
| hsa04668 | TNF signaling pathway | 0.00 | 0.00 |
| hsa04010 | MAPK signaling pathway | 0.00 | 0.00 |
| hsa05100 | Bacterial invasion of epithelial cells | 0.00 | 0.00 |
| hsa04218 | Cellular senescence | 0.00 | 0.00 |
| hsa05211 | Renal cell carcinoma | 0.00 | 0.00 |
| hsa05212 | Pancreatic cancer | 0.00 | 0.00 |
| hsa05222 | Small cell lung cancer | 0.00 | 0.00 |
| hsa01232 | Nucleotide metabolism | 0.00 | 0.00 |
| hsa05205 | Proteoglycans in cancer | 0.00 | 0.00 |
| hsa04110 | Cell cycle | 0.00 | 0.00 |
| hsa04064 | NF-kappa B signaling pathway | 0.00 | 0.00 |
| hsa04660 | T cell receptor signaling pathway | 0.00 | 0.00 |
| hsa05418 | Fluid shear stress and atherosclerosis | 0.00 | 0.00 |
| hsa04115 | p53 signaling pathway | 0.00 | 0.00 |
| hsa05170 | Human immunodeficiency virus 1 infection | 0.00 | 0.00 |
| hsa05133 | Pertussis | 0.00 | 0.00 |
| hsa05171 | Coronavirus disease - COVID-19 | 0.00 | 0.00 |
| hsa04150 | mTOR signaling pathway | 0.00 | 0.00 |
| hsa05146 | Amoebiasis | 0.00 | 0.00 |
| hsa04141 | Protein processing in endoplasmic reticulum | 0.00 | 0.00 |
| hsa04142 | Lysosome | 0.00 | 0.00 |
| hsa05165 | Human papillomavirus infection | 0.00 | 0.00 |
| hsa00520 | Amino sugar and nucleotide sugar metabolism | 0.00 | 0.00 |
| hsa05120 | Epithelial cell signaling in Helicobacter pylori infection | 0.00 | 0.00 |
| hsa04066 | HIF-1 signaling pathway | 0.00 | 0.00 |
| hsa00562 | Inositol phosphate metabolism | 0.00 | 0.00 |
| hsa01524 | Platinum drug resistance | 0.00 | 0.00 |
| hsa05220 | Chronic myeloid leukemia | 0.00 | 0.00 |
| hsa05216 | Thyroid cancer | 0.00 | 0.00 |
| hsa04611 | Platelet activation | 0.00 | 0.00 |
| hsa05417 | Lipid and atherosclerosis | 0.00 | 0.00 |
| hsa05167 | Kaposi sarcoma-associated herpesvirus infection | 0.00 | 0.00 |
| hsa04659 | Th17 cell differentiation | 0.00 | 0.00 |
| hsa04146 | Peroxisome | 0.00 | 0.00 |
| hsa04662 | B cell receptor signaling pathway | 0.00 | 0.00 |
| hsa04137 | Mitophagy - animal | 0.00 | 0.00 |
| hsa04120 | Ubiquitin mediated proteolysis | 0.00 | 0.00 |
| hsa05213 | Endometrial cancer | 0.00 | 0.00 |
| hsa04015 | Rap1 signaling pathway | 0.00 | 0.00 |
| hsa05161 | Hepatitis B | 0.00 | 0.00 |
| hsa04070 | Phosphatidylinositol signaling system | 0.00 | 0.00 |
| hsa05215 | Prostate cancer | 0.00 | 0.00 |
| hsa05152 | Tuberculosis | 0.00 | 0.00 |
| hsa05140 | Leishmaniasis | 0.00 | 0.00 |
| hsa04360 | Axon guidance | 0.00 | 0.00 |
| hsa03060 | Protein export | 0.00 | 0.00 |
| hsa03050 | Proteasome | 0.00 | 0.00 |
| hsa00310 | Lysine degradation | 0.00 | 0.00 |
| hsa01523 | Antifolate resistance | 0.00 | 0.01 |
| hsa05208 | Chemical carcinogenesis - reactive oxygen species | 0.00 | 0.01 |
| hsa00280 | Valine, leucine and isoleucine degradation | 0.00 | 0.01 |
| hsa01250 | Biosynthesis of nucleotide sugars | 0.00 | 0.01 |
| hsa04530 | Tight junction | 0.00 | 0.01 |
| hsa04062 | Chemokine signaling pathway | 0.00 | 0.01 |
| hsa05221 | Acute myeloid leukemia | 0.00 | 0.01 |
| hsa05164 | Influenza A | 0.00 | 0.01 |
| hsa04670 | Leukocyte transendothelial migration | 0.00 | 0.01 |
| hsa03040 | Spliceosome | 0.00 | 0.01 |
| hsa05416 | Viral myocarditis | 0.00 | 0.01 |
| hsa05012 | Parkinson disease | 0.00 | 0.01 |
| hsa01522 | Endocrine resistance | 0.00 | 0.01 |
| hsa04910 | Insulin signaling pathway | 0.00 | 0.01 |
| hsa04012 | ErbB signaling pathway | 0.00 | 0.01 |
| hsa04613 | Neutrophil extracellular trap formation | 0.00 | 0.01 |
| hsa04657 | IL-17 signaling pathway | 0.00 | 0.01 |
| hsa04370 | VEGF signaling pathway | 0.00 | 0.01 |
| hsa04928 | Parathyroid hormone synthesis, secretion and action | 0.00 | 0.01 |
| hsa05225 | Hepatocellular carcinoma | 0.01 | 0.01 |
| hsa01240 | Biosynthesis of cofactors | 0.01 | 0.01 |
| hsa05014 | Amyotrophic lateral sclerosis | 0.01 | 0.01 |
| hsa03440 | Homologous recombination | 0.01 | 0.01 |
| hsa04216 | Ferroptosis | 0.01 | 0.01 |
| hsa05219 | Bladder cancer | 0.01 | 0.01 |
| hsa03430 | Mismatch repair | 0.01 | 0.01 |
| hsa04722 | Neurotrophin signaling pathway | 0.01 | 0.01 |
| hsa05010 | Alzheimer disease | 0.01 | 0.01 |
| hsa04072 | Phospholipase D signaling pathway | 0.01 | 0.02 |
| hsa04210 | Apoptosis | 0.01 | 0.02 |
| hsa04919 | Thyroid hormone signaling pathway | 0.01 | 0.02 |
| hsa01200 | Carbon metabolism | 0.01 | 0.02 |
| hsa00510 | N-Glycan biosynthesis | 0.01 | 0.02 |
| hsa05020 | Prion disease | 0.01 | 0.02 |
| hsa00071 | Fatty acid degradation | 0.01 | 0.02 |
| hsa04926 | Relaxin signaling pathway | 0.01 | 0.02 |
| hsa00230 | Purine metabolism | 0.01 | 0.02 |
| hsa05415 | Diabetic cardiomyopathy | 0.01 | 0.02 |
| hsa05160 | Hepatitis C | 0.01 | 0.02 |
| hsa04625 | C-type lectin receptor signaling pathway | 0.01 | 0.02 |
| hsa05231 | Choline metabolism in cancer | 0.01 | 0.02 |
| hsa04936 | Alcoholic liver disease | 0.01 | 0.02 |
| hsa05162 | Measles | 0.01 | 0.02 |
| hsa04917 | Prolactin signaling pathway | 0.01 | 0.02 |
| hsa03018 | RNA degradation | 0.02 | 0.02 |
| hsa04512 | ECM-receptor interaction | 0.02 | 0.02 |
| hsa05134 | Legionellosis | 0.02 | 0.03 |
| hsa00410 | beta-Alanine metabolism | 0.02 | 0.03 |
| hsa00564 | Glycerophospholipid metabolism | 0.02 | 0.03 |
| hsa05214 | Glioma | 0.02 | 0.03 |
| hsa04912 | GnRH signaling pathway | 0.02 | 0.03 |
| hsa05323 | Rheumatoid arthritis | 0.02 | 0.03 |
| hsa00330 | Arginine and proline metabolism | 0.02 | 0.03 |
| hsa04330 | Notch signaling pathway | 0.02 | 0.03 |
| hsa05202 | Transcriptional misregulation in cancer | 0.02 | 0.03 |
| hsa05321 | Inflammatory bowel disease | 0.02 | 0.03 |
| hsa04664 | Fc epsilon RI signaling pathway | 0.02 | 0.03 |
| hsa04310 | Wnt signaling pathway | 0.02 | 0.03 |
| hsa05022 | Pathways of neurodegeneration - multiple diseases | 0.02 | 0.04 |
| hsa04932 | Non-alcoholic fatty liver disease | 0.03 | 0.04 |
| hsa04130 | SNARE interactions in vesicular transport | 0.03 | 0.04 |
| hsa01521 | EGFR tyrosine kinase inhibitor resistance | 0.03 | 0.04 |
| hsa04014 | Ras signaling pathway | 0.03 | 0.04 |
| hsa03013 | Nucleocytoplasmic transport | 0.03 | 0.04 |
| hsa05142 | Chagas disease | 0.03 | 0.04 |
| hsa04714 | Thermogenesis | 0.03 | 0.04 |
| hsa05322 | Systemic lupus erythematosus | 0.03 | 0.04 |
| hsa04612 | Antigen processing and presentation | 0.03 | 0.04 |
| hsa00480 | Glutathione metabolism | 0.03 | 0.04 |
| hsa04621 | NOD-like receptor signaling pathway | 0.03 | 0.04 |
| hsa00640 | Propanoate metabolism | 0.03 | 0.04 |
| hsa00340 | Histidine metabolism | 0.03 | 0.04 |
| hsa05340 | Primary immunodeficiency | 0.04 | 0.05 |
| hsa05163 | Human cytomegalovirus infection | 0.04 | 0.05 |
| hsa04211 | Longevity regulating pathway | 0.04 | 0.05 |

**Abbreviations:** P-value: Probability; **q-value**: Adjusted P-value.
