# Supplementary material for: Gefitinib metabolism-related lncRNAs for the prediction of prognosis, tumor microenvironment and drug sensitivity in lung adenocarcinoma
Source: Sci Rep. 2024 May 6;14:10348. doi: 10.1038/s41598-024-61175-3 (PMC11074108; doi:10.1038/s41598-024-61175-3)
Supplement: Supplementary file 22 — Supplementary Table S8. [file 41598_2024_61175_MOESM22_ESM.docx]

**Table S8** Results of GSEA in the high-risk group.

| **Category** | **NES** | **P-value** |
| --- | --- | --- |
| **BIOCRATA** |  |  |
| BIOCARTA_NUCLEARRS_PATHWAY | 1.96 | 0.00 |
| BIOCARTA_AGR_PATHWAY | 1.95 | 0.00 |
| BIOCARTA_ARAP_PATHWAY | 1.90 | 0.00 |
| BIOCARTA_TGFB_PATHWAY | 1.82 | 0.01 |
| BIOCARTA_CERAMIDE_PATHWAY | 1.71 | 0.02 |
| BIOCARTA_P38MAPK_PATHWAY | 1.71 | 0.03 |
| BIOCARTA_CXCR4_PATHWAY | 1.66 | 0.03 |
| BIOCARTA_PTDINS_PATHWAY | 1.62 | 0.04 |
| **GO** |  |  |
| GOCC_GAP_JUNCTION | 0.64 | 0.00 |
| GOBP_NEGATIVE_REGULATION_OF_STEM_CELL_PROLIFERATION | 0.66 | 0.00 |
| GOBP_NEGATIVE_REGULATION_OF_MIRNA_METABOLIC_PROCESS | 0.74 | 0.00 |
| GOBP_REGULATION_OF_VIRAL_INDUCED_CYTOPLASMIC_PATTERN_RECOGNITION_RECEPTOR_SIGNALING_PATHWAY | 0.70 | 0.00 |
| GOBP_CYTOPLASMIC_PATTERN_RECOGNITION_RECEPTOR_SIGNALING_PATHWAY_IN_RESPONSE_TO_VIRUS | 0.68 | 0.00 |
| GOBP_REGULATION_OF_RIG_I_SIGNALING_PATHWAY | 0.72 | 0.00 |
| GOBP_MESODERMAL_CELL_DIFFERENTIATION | 0.61 | 0.00 |
| GOBP_RESPONSE_TO_PROGESTERONE | 0.61 | 0.00 |
| GOBP_REGULATION_OF_HAIR_CYCLE | 0.59 | 0.00 |
| GOBP_TELOMERE_MAINTENANCE_VIA_RECOMBINATION | 0.71 | 0.00 |
| GOBP_RIG_I_SIGNALING_PATHWAY | 0.70 | 0.00 |
| GOMF_GAP_JUNCTION_CHANNEL_ACTIVITY | 0.64 | 0.00 |
| GOBP_MICROVILLUS_ASSEMBLY | 0.69 | 0.00 |
| GOMF_OXIDOREDUCTASE_ACTIVITY_ACTING_ON_PAIRED_DONORS_WITH_INCORPORATION_OR_REDUCTION_OF_MOLECULAR_OXYGEN_REDUCED_FLAVIN_OR_FLAVOPROTEIN_AS_ONE_DONOR_AND_INCORPORATION_OF_ONE_ATOM_OF_OXYGEN | 0.57 | 0.00 |
| GOBP_EPITHELIAL_STRUCTURE_MAINTENANCE | 0.59 | 0.00 |
| GOBP_MICROVILLUS_ORGANIZATION | 0.70 | 0.00 |
| GOBP_OLIGOSACCHARIDE_BIOSYNTHETIC_PROCESS | 0.57 | 0.00 |
| GOBP_REGULATION_OF_SECONDARY_METABOLIC_PROCESS | 0.67 | 0.00 |
| GOBP_PLATELET_MORPHOGENESIS | 0.70 | 0.00 |
| GOBP_RESPONSE_TO_IMMOBILIZATION_STRESS | 0.61 | 0.00 |
| GOBP_LIPID_PHOSPHORYLATION | 0.65 | 0.00 |
| GOMF_WIDE_PORE_CHANNEL_ACTIVITY | 0.60 | 0.00 |
| GOBP_INNER_EAR_RECEPTOR_CELL_STEREOCILIUM_ORGANIZATION | 0.55 | 0.00 |
| GOBP_AMINE_CATABOLIC_PROCESS | 0.60 | 0.00 |
| GOCC_CONNEXIN_COMPLEX | 0.64 | 0.01 |
| GOMF_SOLUTE_POTASSIUM_ANTIPORTER_ACTIVITY | 0.63 | 0.01 |
| GOMF_METAL_CATION_MONOATOMIC_CATION_ANTIPORTER_ACTIVITY | 0.57 | 0.01 |
| GOBP_POSITIVE_REGULATION_OF_PROTEIN_MATURATION | 0.57 | 0.01 |
| GOCC_MYOSIN_COMPLEX | 0.54 | 0.01 |
| GOBP_WOUND_HEALING_SPREADING_OF_EPIDERMAL_CELLS | 0.70 | 0.01 |
| GOBP_CYTOPLASMIC_PATTERN_RECOGNITION_RECEPTOR_SIGNALING_PATHWAY | 0.57 | 0.01 |
| GOBP_PHOTORECEPTOR_CELL_MAINTENANCE | 0.53 | 0.01 |
| GOBP_CELLULAR_RESPONSE_TO_VIRUS | 0.52 | 0.01 |
| GOBP_NEGATIVE_REGULATION_OF_RECEPTOR_INTERNALIZATION | 0.63 | 0.01 |
| GOMF_PHOSPHOLIPID_SCRAMBLASE_ACTIVITY | 0.60 | 0.01 |
| GOBP_NEGATIVE_REGULATION_OF_GLYCOPROTEIN_METABOLIC_PROCESS | 0.51 | 0.01 |
| GOBP_NEGATIVE_REGULATION_OF_KERATINOCYTE_PROLIFERATION | 0.63 | 0.01 |
| GOMF_AROMATASE_ACTIVITY | 0.64 | 0.01 |
| GOMF_CARGO_ADAPTOR_ACTIVITY | 0.69 | 0.01 |
| GOBP_POSITIVE_REGULATION_OF_AMYLOID_BETA_FORMATION | 0.59 | 0.01 |
| GOCC_BRUSH_BORDER | 0.51 | 0.01 |
| GOBP_NEGATIVE_REGULATION_OF_CELL_MATRIX_ADHESION | 0.58 | 0.01 |
| GOBP_RETINAL_METABOLIC_PROCESS | 0.56 | 0.01 |
| GOBP_NEGATIVE_REGULATION_OF_EPITHELIAL_CELL_MIGRATION | 0.45 | 0.01 |
| GOMF_ARACHIDONIC_ACID_MONOOXYGENASE_ACTIVITY | 0.67 | 0.01 |
| GOBP_SECONDARY_METABOLITE_BIOSYNTHETIC_PROCESS | 0.54 | 0.01 |
| GOBP_REGULATION_OF_HAIR_FOLLICLE_DEVELOPMENT | 0.59 | 0.01 |
| GOBP_EPOXYGENASE_P450_PATHWAY | 0.68 | 0.01 |
| GOBP_REGULATION_OF_MUSCLE_ORGAN_DEVELOPMENT | 0.59 | 0.01 |
| GOMF_MUSCLE_ALPHA_ACTININ_BINDING | 0.66 | 0.01 |
| GOBP_NEGATIVE_REGULATION_OF_OSTEOBLAST_DIFFERENTIATION | 0.52 | 0.01 |
| GOCC_FILAMENTOUS_ACTIN | 0.61 | 0.01 |
| GOBP_REGULATION_OF_RUFFLE_ASSEMBLY | 0.60 | 0.01 |
| GOMF_SODIUM_PROTON_ANTIPORTER_ACTIVITY | 0.69 | 0.01 |
| GOBP_POSITIVE_REGULATION_OF_TRANSFORMING_GROWTH_FACTOR_BETA_PRODUCTION | 0.60 | 0.01 |
| GOCC_ACTIN_FILAMENT_BUNDLE | 0.65 | 0.01 |
| GOCC_FILOPODIUM_TIP | 0.64 | 0.01 |
| GOMF_ARACHIDONIC_ACID_EPOXYGENASE_ACTIVITY | 0.71 | 0.01 |
| GOBP_POSITIVE_REGULATION_OF_EPIDERMIS_DEVELOPMENT | 0.52 | 0.01 |
| GOBP_NEGATIVE_REGULATION_OF_PROTEIN_IMPORT_INTO_NUCLEUS | 0.62 | 0.01 |
| GOBP_NEGATIVE_REGULATION_OF_TYPE_I_INTERFERON_MEDIATED_SIGNALING_PATHWAY | 0.69 | 0.01 |
| GOCC_ACTOMYOSIN | 0.65 | 0.01 |
| GOBP_EPIDERMIS_DEVELOPMENT | 0.44 | 0.01 |
| GOBP_NEGATIVE_REGULATION_OF_GLUCOSE_IMPORT | 0.61 | 0.01 |
| GOBP_SULFATION | 0.56 | 0.01 |
| GOBP_NEGATIVE_REGULATION_OF_DEFENSE_RESPONSE_TO_VIRUS | 0.63 | 0.01 |
| GOBP_NEGATIVE_REGULATION_OF_SIGNALING_RECEPTOR_ACTIVITY | 0.46 | 0.01 |
| GOBP_POSITIVE_REGULATION_OF_RUFFLE_ASSEMBLY | 0.72 | 0.01 |
| GOBP_ENDODERM_FORMATION | 0.56 | 0.01 |
| GOCC_NUCLEAR_OUTER_MEMBRANE | 0.59 | 0.01 |
| GOBP_INNER_EAR_RECEPTOR_CELL_DEVELOPMENT | 0.48 | 0.01 |
| GOCC_CLUSTER_OF_ACTIN_BASED_CELL_PROJECTIONS | 0.44 | 0.02 |
| GOBP_ACTIN_FILAMENT_BASED_TRANSPORT | 0.65 | 0.02 |
| GOCC_BASEMENT_MEMBRANE | 0.55 | 0.02 |
| GOCC_TRANS_GOLGI_NETWORK_TRANSPORT_VESICLE_MEMBRANE | 0.64 | 0.02 |
| GOMF_METAL_CATION_PROTON_ANTIPORTER_ACTIVITY | 0.61 | 0.02 |
| GOMF_PHOSPHATIDIC_ACID_BINDING | 0.59 | 0.02 |
| GOBP_BONE_GROWTH | 0.54 | 0.02 |
| GOMF_PROTEIN_TYROSINE_KINASE_ACTIVATOR_ACTIVITY | 0.53 | 0.02 |
| GOMF_SPHINGOLIPID_TRANSPORTER_ACTIVITY | 0.59 | 0.02 |
| GOBP_EPIBOLY | 0.64 | 0.02 |
| GOBP_LENS_FIBER_CELL_DIFFERENTIATION | 0.52 | 0.02 |
| GOBP_NEGATIVE_REGULATION_OF_CHONDROCYTE_DIFFERENTIATION | 0.60 | 0.02 |
| GOBP_REGULATION_OF_ANOIKIS | 0.57 | 0.02 |
| GOBP_KERATINOCYTE_PROLIFERATION | 0.52 | 0.02 |
| GOMF_STRUCTURAL_CONSTITUENT_OF_MUSCLE | 0.55 | 0.02 |
| GOBP_TERPENOID_METABOLIC_PROCESS | 0.45 | 0.02 |
| GOBP_RUFFLE_ORGANIZATION | 0.56 | 0.02 |
| GOBP_ZYMOGEN_ACTIVATION | 0.49 | 0.02 |
| GOBP_NEGATIVE_REGULATION_OF_VIRAL_PROCESS | 0.56 | 0.02 |
| GOBP_PROTEIN_LOCALIZATION_TO_CELL_CELL_JUNCTION | 0.67 | 0.02 |
| GOBP_REGULATION_OF_INTEGRIN_ACTIVATION | 0.67 | 0.02 |
| GOBP_CELL_DIFFERENTIATION_INVOLVED_IN_EMBRYONIC_PLACENTA_DEVELOPMENT | 0.61 | 0.02 |
| GOCC_LAMELLIPODIUM_MEMBRANE | 0.66 | 0.02 |
| GOBP_KERATAN_SULFATE_METABOLIC_PROCESS | 0.62 | 0.02 |
| GOBP_REGULATION_OF_WATER_LOSS_VIA_SKIN | 0.59 | 0.02 |
| GOBP_NEGATIVE_REGULATION_OF_RECEPTOR_MEDIATED_ENDOCYTOSIS | 0.50 | 0.02 |
| GOBP_MYOBLAST_PROLIFERATION | 0.50 | 0.02 |
| GOBP_NEGATIVE_REGULATION_OF_VIRAL_GENOME_REPLICATION | 0.60 | 0.02 |
| GOBP_MAINTENANCE_OF_GASTROINTESTINAL_EPITHELIUM | 0.60 | 0.02 |
| GOBP_BLOOD_VESSEL_REMODELING | 0.50 | 0.02 |
| GOMF_VOLTAGE_GATED_ANION_CHANNEL_ACTIVITY | 0.63 | 0.02 |
| GOBP_ERBB_SIGNALING_PATHWAY | 0.48 | 0.02 |
| GOBP_BLOOD_VESSEL_ENDOTHELIAL_CELL_MIGRATION | 0.45 | 0.02 |
| GOBP_REGULATION_OF_BLOOD_VESSEL_ENDOTHELIAL_CELL_MIGRATION | 0.44 | 0.02 |
| GOBP_BUNDLE_OF_HIS_CELL_TO_PURKINJE_MYOCYTE_COMMUNICATION | 0.60 | 0.02 |
| GOMF_ACTIN_FILAMENT_BINDING | 0.51 | 0.02 |
| GOBP_NEGATIVE_REGULATION_OF_CHEMOKINE_PRODUCTION | 0.55 | 0.02 |
| GOBP_SKIN_DEVELOPMENT | 0.44 | 0.02 |
| GOBP_REGULATION_OF_MEMBRANE_PROTEIN_ECTODOMAIN_PROTEOLYSIS | 0.59 | 0.02 |
| GOBP_PYRIDINE_NUCLEOTIDE_BIOSYNTHETIC_PROCESS | 0.54 | 0.02 |
| GOBP_RETINOIC_ACID_METABOLIC_PROCESS | 0.53 | 0.02 |
| GOMF_CYCLASE_REGULATOR_ACTIVITY | 0.62 | 0.02 |
| GOMF_ACTIN_BINDING | 0.48 | 0.02 |
| GOBP_POSITIVE_REGULATION_OF_FATTY_ACID_OXIDATION | 0.58 | 0.02 |
| GOBP_MEGAKARYOCYTE_DIFFERENTIATION | 0.47 | 0.02 |
| GOBP_VITAMIN_CATABOLIC_PROCESS | 0.63 | 0.02 |
| GOBP_AMINOGLYCAN_BIOSYNTHETIC_PROCESS | 0.49 | 0.02 |
| GOBP_NEGATIVE_REGULATION_OF_NIK_NF_KAPPAB_SIGNALING | 0.51 | 0.02 |
| GOMF_CADHERIN_BINDING_INVOLVED_IN_CELL_CELL_ADHESION | 0.73 | 0.02 |
| GOMF_GALACTOSYLTRANSFERASE_ACTIVITY | 0.57 | 0.02 |
| GOBP_SUPPRESSION_OF_VIRAL_RELEASE_BY_HOST | 0.69 | 0.02 |
| GOBP_MESODERMAL_CELL_FATE_COMMITMENT | 0.58 | 0.02 |
| GOBP_POSITIVE_REGULATION_OF_OXIDATIVE_STRESS_INDUCED_CELL_DEATH | 0.54 | 0.02 |
| GOBP_REGULATION_OF_MIRNA_TRANSCRIPTION | 0.56 | 0.02 |
| GOMF_RETINAL_BINDING | 0.54 | 0.02 |
| GOBP_ANGIOTENSIN_ACTIVATED_SIGNALING_PATHWAY | 0.61 | 0.02 |
| GOBP_ENDOTHELIAL_CELL_MIGRATION | 0.43 | 0.02 |
| GOBP_MICROTUBULE_ORGANIZING_CENTER_LOCALIZATION | 0.57 | 0.02 |
| GOBP_POSITIVE_REGULATION_OF_SODIUM_ION_TRANSMEMBRANE_TRANSPORT | 0.59 | 0.02 |
| GOCC_FILOPODIUM_MEMBRANE | 0.62 | 0.02 |
| GOCC_ACTIN_FILAMENT | 0.52 | 0.02 |
| GOMF_EPIDERMAL_GROWTH_FACTOR_RECEPTOR_BINDING | 0.52 | 0.02 |
| GOBP_AROMATIC_AMINO_ACID_FAMILY_CATABOLIC_PROCESS | 0.66 | 0.02 |
| GOCC_CORTICAL_ACTIN_CYTOSKELETON | 0.56 | 0.02 |
| GOBP_GLYCOSYL_COMPOUND_BIOSYNTHETIC_PROCESS | 0.62 | 0.02 |
| GOBP_ADHERENS_JUNCTION_ORGANIZATION | 0.57 | 0.02 |
| GOBP_MYELIN_MAINTENANCE | 0.61 | 0.02 |
| GOBP_NEGATIVE_REGULATION_OF_MUSCLE_HYPERTROPHY | 0.50 | 0.02 |
| GOBP_BRANCHING_INVOLVED_IN_MAMMARY_GLAND_DUCT_MORPHOGENESIS | 0.55 | 0.02 |
| GOCC_SPERM_PRINCIPAL_PIECE | 0.50 | 0.03 |
| GOBP_TISSUE_MIGRATION | 0.43 | 0.03 |
| GOMF_CALCIUM_ION_TRANSMEMBRANE_TRANSPORTER_ACTIVITY | 0.40 | 0.03 |
| GOCC_PODOSOME | 0.66 | 0.03 |
| GOBP_REGULATION_OF_EPITHELIAL_CELL_MIGRATION | 0.43 | 0.03 |
| GOBP_ESTROGEN_METABOLIC_PROCESS | 0.47 | 0.03 |
| GOBP_APICAL_PROTEIN_LOCALIZATION | 0.65 | 0.03 |
| GOBP_EPIDERMAL_CELL_DIFFERENTIATION | 0.42 | 0.03 |
| GOCC_CLATHRIN_VESICLE_COAT | 0.63 | 0.03 |
| GOBP_MYELOID_DENDRITIC_CELL_DIFFERENTIATION | 0.57 | 0.03 |
| GOBP_KERATINOCYTE_DIFFERENTIATION | 0.45 | 0.03 |
| GOBP_RUFFLE_ASSEMBLY | 0.54 | 0.03 |
| GOBP_INTERFERON_BETA_PRODUCTION | 0.56 | 0.03 |
| GOMF_ENDOPEPTIDASE_ACTIVITY | 0.35 | 0.03 |
| GOMF_SECONDARY_ACTIVE_MONOCARBOXYLATE_TRANSMEMBRANE_TRANSPORTER_ACTIVITY | 0.56 | 0.03 |
| GOBP_DNA_DAMAGE_RESPONSE_SIGNAL_TRANSDUCTION_BY_P53_CLASS_MEDIATOR_RESULTING_IN_CELL_CYCLE_ARREST | 0.55 | 0.03 |
| GOBP_FORMATION_OF_PRIMARY_GERM_LAYER | 0.49 | 0.03 |
| GOBP_MOLTING_CYCLE | 0.44 | 0.03 |
| GOCC_GOLGI_CISTERNA_MEMBRANE | 0.46 | 0.03 |
| GOBP_NEGATIVE_REGULATION_OF_PROTEIN_LOCALIZATION_TO_CELL_PERIPHERY | 0.57 | 0.03 |
| GOBP_GLAND_MORPHOGENESIS | 0.44 | 0.03 |
| GOBP_REGULATION_OF_STRIATED_MUSCLE_CONTRACTION | 0.41 | 0.03 |
| GOBP_NEGATIVE_REGULATION_OF_AMYLOID_PRECURSOR_PROTEIN_BIOSYNTHETIC_PROCESS | 0.51 | 0.03 |
| GOBP_POSITIVE_REGULATION_OF_ERBB_SIGNALING_PATHWAY | 0.51 | 0.03 |
| GOBP_XENOBIOTIC_METABOLIC_PROCESS | 0.43 | 0.03 |
| GOCC_CELL_DIVISION_SITE | 0.55 | 0.03 |
| GOBP_SARCOMERE_ORGANIZATION | 0.51 | 0.03 |
| GOBP_PLASMA_MEMBRANE_ORGANIZATION | 0.43 | 0.03 |
| GOBP_POSITIVE_REGULATION_OF_MEMBRANE_PROTEIN_ECTODOMAIN_PROTEOLYSIS | 0.62 | 0.03 |
| GOMF_MICROFILAMENT_MOTOR_ACTIVITY | 0.54 | 0.03 |
| GOBP_VITAMIN_BIOSYNTHETIC_PROCESS | 0.53 | 0.03 |
| GOMF_PROTEASE_BINDING | 0.47 | 0.03 |
| GOBP_UTERUS_DEVELOPMENT | 0.58 | 0.03 |
| GOBP_POSITIVE_REGULATION_OF_ACTIN_FILAMENT_POLYMERIZATION | 0.52 | 0.03 |
| GOCC_MICROVILLUS | 0.45 | 0.03 |
| GOCC_MICROVILLUS_MEMBRANE | 0.56 | 0.03 |
| GOBP_WHITE_FAT_CELL_DIFFERENTIATION | 0.60 | 0.03 |
| GOBP_PYRIDINE_CONTAINING_COMPOUND_BIOSYNTHETIC_PROCESS | 0.52 | 0.03 |
| GOBP_MEMBRANE_BIOGENESIS | 0.53 | 0.03 |
| GOBP_EPHRIN_RECEPTOR_SIGNALING_PATHWAY | 0.50 | 0.03 |
| GOBP_REGULATION_OF_MYOBLAST_PROLIFERATION | 0.50 | 0.03 |
| GOBP_POSITIVE_REGULATION_OF_T_CELL_RECEPTOR_SIGNALING_PATHWAY | 0.66 | 0.03 |
| GOBP_POSITIVE_REGULATION_OF_I_KAPPAB_KINASE_NF_KAPPAB_SIGNALING | 0.47 | 0.03 |
| GOBP_REGULATION_OF_MIRNA_METABOLIC_PROCESS | 0.51 | 0.03 |
| GOCC_TRANS_GOLGI_NETWORK_TRANSPORT_VESICLE | 0.56 | 0.03 |
| GOBP_KERATINOCYTE_MIGRATION | 0.57 | 0.03 |
| GOBP_POSITIVE_REGULATION_OF_CARDIOCYTE_DIFFERENTIATION | 0.63 | 0.03 |
| GOCC_ADHERENS_JUNCTION | 0.50 | 0.03 |
| GOCC_ACTIN_CYTOSKELETON | 0.46 | 0.03 |
| GOBP_REGULATION_OF_PLASMA_MEMBRANE_ORGANIZATION | 0.66 | 0.03 |
| GOBP_NEGATIVE_REGULATION_OF_BLOOD_VESSEL_ENDOTHELIAL_CELL_MIGRATION | 0.43 | 0.03 |
| GOBP_BRANCHING_INVOLVED_IN_SALIVARY_GLAND_MORPHOGENESIS | 0.58 | 0.03 |
| GOCC_GAMMA_TUBULIN_COMPLEX | 0.66 | 0.03 |
| GOBP_PHOSPHATIDIC_ACID_METABOLIC_PROCESS | 0.51 | 0.03 |
| GOBP_EMBRYONIC_SKELETAL_JOINT_DEVELOPMENT | 0.62 | 0.03 |
| GOBP_RESPONSE_TO_GONADOTROPIN | 0.48 | 0.03 |
| GOCC_CELL_CORTEX | 0.45 | 0.03 |
| GOMF_PHOSPHATIDYLGLYCEROL_BINDING | 0.62 | 0.03 |
| GOBP_ENDODERM_DEVELOPMENT | 0.50 | 0.03 |
| GOBP_REGULATION_OF_CELL_MIGRATION_INVOLVED_IN_SPROUTING_ANGIOGENESIS | 0.44 | 0.03 |
| GOBP_ERBB2_SIGNALING_PATHWAY | 0.59 | 0.03 |
| GOBP_REGULATION_OF_GLYCOPROTEIN_METABOLIC_PROCESS | 0.40 | 0.03 |
| GOBP_EXOCRINE_SYSTEM_DEVELOPMENT | 0.48 | 0.03 |
| GOBP_POSITIVE_REGULATION_OF_CARDIAC_MUSCLE_CELL_PROLIFERATION | 0.52 | 0.03 |
| GOBP_CELL_MATRIX_ADHESION | 0.47 | 0.04 |
| GOMF_CHOLESTEROL_BINDING | 0.47 | 0.04 |
| GOBP_RETINOL_METABOLIC_PROCESS | 0.47 | 0.04 |
| GOBP_NEGATIVE_REGULATION_OF_CELL_SUBSTRATE_ADHESION | 0.51 | 0.04 |
| GOMF_SEMAPHORIN_RECEPTOR_BINDING | 0.60 | 0.04 |
| GOMF_DEATH_RECEPTOR_BINDING | 0.60 | 0.04 |
| GOBP_REGULATION_OF_VIRAL_TRANSCRIPTION | 0.61 | 0.04 |
| GOBP_REGULATION_OF_CARDIAC_MUSCLE_CELL_ACTION_POTENTIAL | 0.49 | 0.04 |
| GOBP_MEMBRANE_PROTEIN_ECTODOMAIN_PROTEOLYSIS | 0.52 | 0.04 |
| GOBP_LINOLEIC_ACID_METABOLIC_PROCESS | 0.52 | 0.04 |
| GOBP_CELLULAR_RESPONSE_TO_MECHANICAL_STIMULUS | 0.48 | 0.04 |
| GOBP_ORGANIC_HYDROXY_COMPOUND_CATABOLIC_PROCESS | 0.41 | 0.04 |
| GOBP_NEGATIVE_REGULATION_OF_NUCLEOCYTOPLASMIC_TRANSPORT | 0.54 | 0.04 |
| GOMF_ANTIPORTER_ACTIVITY | 0.45 | 0.04 |
| GOBP_POSITIVE_REGULATION_OF_EPIDERMAL_CELL_DIFFERENTIATION | 0.49 | 0.04 |
| GOBP_CELL_CELL_JUNCTION_ORGANIZATION | 0.46 | 0.04 |
| GOMF_ORGANIC_ACID_SODIUM_SYMPORTER_ACTIVITY | 0.45 | 0.04 |
| GOCC_CLATHRIN_COAT | 0.56 | 0.04 |
| GOMF_SODIUM_CHANNEL_REGULATOR_ACTIVITY | 0.46 | 0.04 |
| GOMF_CARD_DOMAIN_BINDING | 0.60 | 0.04 |
| GOBP_REGULATION_OF_PLATELET_DERIVED_GROWTH_FACTOR_RECEPTOR_SIGNALING_PATHWAY | 0.53 | 0.04 |
| GOBP_FUCOSE_METABOLIC_PROCESS | 0.57 | 0.04 |
| GOBP_MORPHOGENESIS_OF_AN_EPITHELIAL_SHEET | 0.52 | 0.04 |
| GOBP_REGULATION_OF_TELOMERE_CAPPING | 0.58 | 0.04 |
| GOBP_AMEBOIDAL_TYPE_CELL_MIGRATION | 0.41 | 0.04 |
| GOBP_ASYMMETRIC_CELL_DIVISION | 0.58 | 0.04 |
| GOBP_NEGATIVE_REGULATION_OF_CYTOKINE_PRODUCTION | 0.38 | 0.04 |
| GOBP_ANOIKIS | 0.54 | 0.04 |
| GOBP_REGULATION_OF_ENDOTHELIAL_CELL_MIGRATION | 0.42 | 0.04 |
| GOBP_NEGATIVE_REGULATION_OF_BLOOD_VESSEL_ENDOTHELIAL_CELL_PROLIFERATION_INVOLVED_IN_SPROUTING_ANGIOGENESIS | 0.50 | 0.04 |
| GOBP_REGULATION_OF_STEM_CELL_PROLIFERATION | 0.41 | 0.04 |
| GOBP_ENAMEL_MINERALIZATION | 0.51 | 0.04 |
| GOMF_PHOSPHATIDYLINOSITOL_4_5_BISPHOSPHATE_BINDING | 0.48 | 0.04 |
| GOBP_NEGATIVE_REGULATION_OF_STEM_CELL_DIFFERENTIATION | 0.58 | 0.04 |
| GOBP_REGULATION_OF_ACTIN_FILAMENT_BASED_MOVEMENT | 0.46 | 0.04 |
| GOBP_POSITIVE_REGULATION_OF_SODIUM_ION_TRANSMEMBRANE_TRANSPORTER_ACTIVITY | 0.59 | 0.04 |
| GOBP_NEGATIVE_REGULATION_OF_TYPE_I_INTERFERON_PRODUCTION | 0.54 | 0.04 |
| GOBP_REGULATION_OF_CARDIAC_MUSCLE_CONTRACTION | 0.41 | 0.04 |
| GOBP_POSITIVE_REGULATION_OF_MUSCLE_CELL_APOPTOTIC_PROCESS | 0.47 | 0.04 |
| GOMF_SOLUTE_CATION_ANTIPORTER_ACTIVITY | 0.50 | 0.04 |
| GOBP_RESPONSE_TO_VIRUS | 0.43 | 0.04 |
| GOBP_WATER_HOMEOSTASIS | 0.43 | 0.04 |
| GOBP_BENZENE_CONTAINING_COMPOUND_METABOLIC_PROCESS | 0.52 | 0.04 |
| GOMF_CELL_CELL_ADHESION_MEDIATOR_ACTIVITY | 0.52 | 0.04 |
| GOCC_RUFFLE | 0.50 | 0.04 |
| GOBP_SALIVARY_GLAND_DEVELOPMENT | 0.53 | 0.04 |
| GOBP_WOUND_HEALING | 0.44 | 0.04 |
| GOBP_REGULATION_OF_STRIATED_MUSCLE_TISSUE_DEVELOPMENT | 0.58 | 0.04 |
| GOBP_MUCOPOLYSACCHARIDE_METABOLIC_PROCESS | 0.46 | 0.04 |
| GOBP_SKELETAL_MUSCLE_CELL_PROLIFERATION | 0.54 | 0.04 |
| GOBP_RETINA_HOMEOSTASIS | 0.38 | 0.04 |
| GOBP_BASEMENT_MEMBRANE_ORGANIZATION | 0.60 | 0.04 |
| GOBP_CD40_SIGNALING_PATHWAY | 0.62 | 0.04 |
| GOBP_PROTEIN_PROCESSING | 0.39 | 0.04 |
| GOBP_REGULATION_OF_SMAD_PROTEIN_SIGNAL_TRANSDUCTION | 0.50 | 0.04 |
| GOBP_ENDODERMAL_CELL_DIFFERENTIATION | 0.56 | 0.04 |
| GOBP_REGULATION_OF_CHONDROCYTE_DIFFERENTIATION | 0.47 | 0.04 |
| GOBP_NIK_NF_KAPPAB_SIGNALING | 0.41 | 0.04 |
| GOBP_PROSTATE_GLAND_MORPHOGENESIS | 0.51 | 0.04 |
| GOBP_NEGATIVE_REGULATION_OF_FAT_CELL_DIFFERENTIATION | 0.47 | 0.04 |
| GOMF_CALCIUM_DEPENDENT_PROTEIN_BINDING | 0.46 | 0.04 |
| GOBP_NUCLEOBASE_CONTAINING_SMALL_MOLECULE_BIOSYNTHETIC_PROCESS | 0.61 | 0.04 |
| GOBP_NEGATIVE_REGULATION_OF_SMOOTH_MUSCLE_CELL_MIGRATION | 0.49 | 0.04 |
| GOBP_REGULATION_OF_PROTEIN_MATURATION | 0.43 | 0.04 |
| GOBP_REGULATION_OF_MORPHOGENESIS_OF_AN_EPITHELIUM | 0.44 | 0.04 |
| GOBP_POSITIVE_REGULATION_OF_EPITHELIAL_CELL_MIGRATION | 0.45 | 0.04 |
| GOMF_CELL_ADHESION_MEDIATOR_ACTIVITY | 0.50 | 0.04 |
| GOCC_CLEAVAGE_FURROW | 0.54 | 0.04 |
| GOMF_METALLOAMINOPEPTIDASE_ACTIVITY | 0.51 | 0.04 |
| GOBP_DEFENSE_RESPONSE_TO_SYMBIONT | 0.45 | 0.04 |
| GOBP_NEGATIVE_REGULATION_OF_CELL_MIGRATION_INVOLVED_IN_SPROUTING_ANGIOGENESIS | 0.47 | 0.04 |
| GOMF_CADHERIN_BINDING | 0.54 | 0.04 |
| GOBP_HEPARIN_METABOLIC_PROCESS | 0.59 | 0.04 |
| GOBP_NEGATIVE_REGULATION_OF_RESPONSE_TO_CYTOKINE_STIMULUS | 0.43 | 0.04 |
| GOBP_POSITIVE_REGULATION_OF_NON_CANONICAL_WNT_SIGNALING_PATHWAY | 0.57 | 0.04 |
| GOBP_REGULATION_OF_PLASMINOGEN_ACTIVATION | 0.60 | 0.04 |
| GOCC_DNA_REPAIR_COMPLEX | 0.60 | 0.04 |
| GOBP_ACTIVATION_OF_NF_KAPPAB_INDUCING_KINASE_ACTIVITY | 0.58 | 0.04 |
| GOBP_POSITIVE_REGULATION_OF_ENDOTHELIAL_CELL_MIGRATION | 0.45 | 0.04 |
| GOBP_REGULATION_OF_SUBSTRATE_ADHESION_DEPENDENT_CELL_SPREADING | 0.52 | 0.04 |
| GOBP_CHONDROCYTE_DIFFERENTIATION_INVOLVED_IN_ENDOCHONDRAL_BONE_MORPHOGENESIS | 0.58 | 0.05 |
| GOCC_CELL_CELL_CONTACT_ZONE | 0.44 | 0.05 |
| GOBP_ACTIN_FILAMENT_SEVERING | 0.65 | 0.05 |
| GOBP_PLASMA_MEMBRANE_TUBULATION | 0.65 | 0.05 |
| GOBP_GOLGI_TO_VACUOLE_TRANSPORT | 0.61 | 0.05 |
| GOMF_PROLINE_RICH_REGION_BINDING | 0.63 | 0.05 |
| GOMF_SECONDARY_ACTIVE_TRANSMEMBRANE_TRANSPORTER_ACTIVITY | 0.35 | 0.05 |
| GOBP_TRANSFORMING_GROWTH_FACTOR_BETA_PRODUCTION | 0.52 | 0.05 |
| GOBP_MAMMARY_GLAND_DUCT_MORPHOGENESIS | 0.51 | 0.05 |
| GOCC_CORTICAL_CYTOSKELETON | 0.50 | 0.05 |
| GOBP_INTERFERON_GAMMA_MEDIATED_SIGNALING_PATHWAY | 0.57 | 0.05 |
| GOBP_REGULATION_OF_ERBB_SIGNALING_PATHWAY | 0.46 | 0.05 |
| GOCC_CENTRIOLAR_SATELLITE | 0.46 | 0.05 |
| GOMF_PHOSPHATIDYLINOSITOL_3_KINASE_BINDING | 0.55 | 0.05 |
| GOBP_REGULATION_OF_T_CELL_DIFFERENTIATION_IN_THYMUS | 0.50 | 0.05 |
| GOCC_I_BAND | 0.45 | 0.05 |
| GOBP_POSITIVE_REGULATION_OF_CELL_MIGRATION_INVOLVED_IN_SPROUTING_ANGIOGENESIS | 0.51 | 0.05 |
| GOCC_MYOSIN_II_COMPLEX | 0.55 | 0.05 |
| GOBP_CHONDROCYTE_DIFFERENTIATION | 0.44 | 0.05 |
| GOMF_LAMININ_BINDING | 0.58 | 0.05 |
| GOBP_PIGMENTATION | 0.43 | 0.05 |
| GOBP_POSITIVE_REGULATION_OF_AMYLOID_PRECURSOR_PROTEIN_CATABOLIC_PROCESS | 0.50 | 0.05 |
| GOMF_MAGNESIUM_ION_TRANSMEMBRANE_TRANSPORTER_ACTIVITY | 0.60 | 0.05 |
| GOBP_CELL_MIGRATION_INVOLVED_IN_SPROUTING_ANGIOGENESIS | 0.44 | 0.05 |
| GOCC_ACTIN_BASED_CELL_PROJECTION | 0.40 | 0.05 |
| GOMF_UDP_GLYCOSYLTRANSFERASE_ACTIVITY | 0.42 | 0.05 |
| GOBP_ACTIN_FILAMENT_ORGANIZATION | 0.44 | 0.05 |
| GOBP_FAT_SOLUBLE_VITAMIN_METABOLIC_PROCESS | 0.43 | 0.05 |
| GOBP_POSITIVE_REGULATION_OF_SMOOTH_MUSCLE_CELL_DIFFERENTIATION | 0.55 | 0.05 |
| **KEGG** |  |  |
| KEGG_O_GLYCAN_BIOSYNTHESIS | 1.90 | 0.01 |
| **PID** |  |  |
| PID_EPHB_FWD_PATHWAY | 1.84 | 0.01 |
| PID_ARF6_TRAFFICKING_PATHWAY | 1.83 | 0.01 |
| PID_ERBB_NETWORK_PATHWAY | 1.68 | 0.02 |
| PID_A6B1_A6B4_INTEGRIN_PATHWAY | 1.82 | 0.02 |
| PID_INTEGRIN_A9B1_PATHWAY | 1.76 | 0.03 |
| PID_FAK_PATHWAY | 1.78 | 0.03 |
| PID_ARF6_DOWNSTREAM_PATHWAY | 1.69 | 0.03 |
| PID_ECADHERIN_KERATINOCYTE_PATHWAY | 1.70 | 0.03 |
| PID_NECTIN_PATHWAY | 1.66 | 0.04 |
| PID_DNA_PK_PATHWAY | 1.59 | 0.04 |
| PID_UPA_UPAR_PATHWAY | 1.68 | 0.04 |
| PID_ECADHERIN_STABILIZATION_PATHWAY | 1.68 | 0.04 |
| PID_ARF6_PATHWAY | 1.62 | 0.04 |
| PID_CDC42_REG_PATHWAY | 1.68 | 0.05 |
| PID_CD40_PATHWAY | 1.65 | 0.05 |
| PID_INTEGRIN_CS_PATHWAY | 1.64 | 0.05 |
| PID_TAP63_PATHWAY | 1.69 | 0.05 |
| **REACTOME** |  |  |
| REACTOME_CELL_JUNCTION_ORGANIZATION | 1.88 | 0.00 |
| REACTOME_EPHRIN_SIGNALING | 1.87 | 0.00 |
| REACTOME_BLOOD_GROUP_SYSTEMS_BIOSYNTHESIS | 1.90 | 0.01 |
| REACTOME_RND3_GTPASE_CYCLE | 1.80 | 0.01 |
| REACTOME_GAP_JUNCTION_TRAFFICKING_AND_REGULATION | 1.77 | 0.01 |
| REACTOME_ANCHORING_FIBRIL_FORMATION | 1.71 | 0.01 |
| REACTOME_RHOF_GTPASE_CYCLE | 1.79 | 0.01 |
| REACTOME_TNF_RECEPTOR_SUPERFAMILY_TNFSF_MEMBERS_MEDIATING_NON_CANONICAL_NF_KB_PATHWAY | 1.75 | 0.01 |
| REACTOME_XENOBIOTICS | 1.73 | 0.01 |
| REACTOME_CELL_CELL_COMMUNICATION | 1.75 | 0.01 |
| REACTOME_NEPHRIN_FAMILY_INTERACTIONS | 1.71 | 0.01 |
| REACTOME_GAP_JUNCTION_ASSEMBLY | 1.69 | 0.02 |
| REACTOME_CELL_EXTRACELLULAR_MATRIX_INTERACTIONS | 1.76 | 0.02 |
| REACTOME_RHOG_GTPASE_CYCLE | 1.81 | 0.02 |
| REACTOME_RHOV_GTPASE_CYCLE | 1.75 | 0.02 |
| REACTOME_KERATAN_SULFATE_KERATIN_METABOLISM | 1.65 | 0.02 |
| REACTOME_LAMININ_INTERACTIONS | 1.76 | 0.02 |
| REACTOME_KERATAN_SULFATE_BIOSYNTHESIS | 1.69 | 0.02 |
| REACTOME_EPH_EPHRIN_SIGNALING | 1.71 | 0.02 |
| REACTOME_G_ALPHA_12_13_SIGNALLING_EVENTS | 1.70 | 0.02 |
| REACTOME_RHOB_GTPASE_CYCLE | 1.71 | 0.03 |
| REACTOME_SEMA4D_IN_SEMAPHORIN_SIGNALING | 1.70 | 0.03 |
| REACTOME_EPH_EPHRIN_MEDIATED_REPULSION_OF_CELLS | 1.65 | 0.03 |
| REACTOME_SIGNALING_BY_PDGF | 1.69 | 0.03 |
| REACTOME_APOPTOTIC_FACTOR_MEDIATED_RESPONSE | 1.63 | 0.03 |
| REACTOME_SIGNALING_BY_ACTIVIN | 1.57 | 0.03 |
| REACTOME_PRE_NOTCH_PROCESSING_IN_GOLGI | 1.62 | 0.03 |
| REACTOME_CASPASE_ACTIVATION_VIA_DEATH_RECEPTORS_IN_THE_PRESENCE_OF_LIGAND | 1.64 | 0.03 |
| REACTOME_SIGNAL_TRANSDUCTION_BY_L1 | 1.65 | 0.03 |
| REACTOME_RHO_GTPASES_ACTIVATE_ROCKS | 1.65 | 0.03 |
| REACTOME_INTERLEUKIN_20_FAMILY_SIGNALING | 1.61 | 0.04 |
| REACTOME_O_LINKED_GLYCOSYLATION_OF_MUCINS | 1.56 | 0.04 |
| REACTOME_THE_CANONICAL_RETINOID_CYCLE_IN_RODS_TWILIGHT_VISION | 1.51 | 0.04 |
| REACTOME_RHOA_GTPASE_CYCLE | 1.69 | 0.04 |
| REACTOME_NRAGE_SIGNALS_DEATH_THROUGH_JNK | 1.65 | 0.04 |
| REACTOME_SEMA4D_INDUCED_CELL_MIGRATION_AND_GROWTH_CONE_COLLAPSE | 1.65 | 0.04 |
| REACTOME_DEGRADATION_OF_THE_EXTRACELLULAR_MATRIX | 1.65 | 0.04 |
| REACTOME_VITAMIN_B5_PANTOTHENATE_METABOLISM | 1.62 | 0.04 |
| REACTOME_SIGNALING_BY_ERBB2_IN_CANCER | 1.56 | 0.04 |
| REACTOME_MET_PROMOTES_CELL_MOTILITY | 1.62 | 0.05 |
| REACTOME_REGULATED_NECROSIS | 1.58 | 0.05 |
| REACTOME_CDC42_GTPASE_CYCLE | 1.68 | 0.05 |
| REACTOME_ELASTIC_FIBRE_FORMATION | 1.65 | 0.05 |
| REACTOME_NON_INTEGRIN_MEMBRANE_ECM_INTERACTIONS | 1.66 | 0.05 |
| **WIKIPATH** |  |  |
| WP_KYNURENINE_PATHWAY_AND_LINKS_TO_CELL_SENESCENCE | 0.00 | 0.00 |
| WP_NEPHROTIC_SYNDROME | 0.00 | 0.47 |
| WP_OSX_AND_MIRNAS_IN_TOOTH_DEVELOPMENT | 0.00 | 0.34 |
| WP_NUCLEAR_RECEPTORS_IN_LIPID_METABOLISM_AND_TOXICITY | 0.00 | 0.39 |
| WP_REGUCALCIN_IN_PROXIMAL_TUBULE_EPITHELIAL_KIDNEY_CELLS | 0.00 | 0.39 |
| WP_PROSURVIVAL_SIGNALING_OF_NEUROPROTECTIN_D1 | 0.00 | 0.47 |
| WP_GLUCOCORTICOID_RECEPTOR_PATHWAY | 0.01 | 0.55 |
| WP_HYPOTHESIZED_PATHWAYS_IN_PATHOGENESIS_OF_CARDIOVASCULAR_DISEASE | 0.01 | 0.32 |
| WP_ARRHYTHMOGENIC_RIGHT_VENTRICULAR_CARDIOMYOPATHY | 0.01 | 0.42 |
| WP_PRIMARY_FOCAL_SEGMENTAL_GLOMERULOSCLEROSIS_FSGS | 0.01 | 0.38 |
| WP_MFAP5_EFFECT_ON_PERMEABILITY_AND_MOTILITY_OF_ENDOTHELIAL_CELLS_VIA_CYTOSKELETON_REARRANGEMENT | 0.01 | 0.38 |
| WP_FOCAL_ADHESION | 0.01 | 0.44 |
| WP_EICOSANOID_METABOLISM_VIA_CYCLOOXYGENASES_COX | 0.02 | 0.38 |
| WP_REGULATION_OF_APOPTOSIS_BY_PARATHYROID_HORMONERELATED_PROTEIN | 0.02 | 0.37 |
| WP_SRF_AND_MIRS_IN_SMOOTH_MUSCLE_DIFFERENTIATION_AND_PROLIFERATION | 0.02 | 0.37 |
| WP_VITAMIN_D_RECEPTOR_PATHWAY | 0.02 | 0.37 |
| WP_TNFRELATED_WEAK_INDUCER_OF_APOPTOSIS_TWEAK_SIGNALING_PATHWAY | 0.02 | 0.38 |
| WP_NUCLEAR_RECEPTORS_METAPATHWAY | 0.02 | 0.38 |
| WP_INTEGRINMEDIATED_CELL_ADHESION | 0.03 | 0.37 |
| WP_NEOVASCULARISATION_PROCESSES | 0.03 | 0.38 |
| WP_MIRNA_TARGETS_IN_ECM_AND_MEMBRANE_RECEPTORS | 0.03 | 0.33 |
| WP_MECHANOREGULATION_AND_PATHOLOGY_OF_YAPTAZ_VIA_HIPPO_AND_NONHIPPO_MECHANISMS | 0.03 | 0.43 |
| WP_TRYPTOPHAN_CATABOLISM_LEADING_TO_NAD_PRODUCTION | 0.03 | 0.39 |
| WP_2873_ARYL_HYDROCARBON_RECEPTOR_PATHWAY | 0.03 | 0.38 |
| WP_GLYCEROLIPIDS_AND_GLYCEROPHOSPHOLIPIDS | 0.03 | 0.40 |
| WP_APOPTOSIS_MODULATION_BY_HSP70 | 0.03 | 0.38 |
| WP_OXIDATION_BY_CYTOCHROME_P450 | 0.03 | 0.41 |
| WP_CANONICAL_AND_NONCANONICAL_TGFB_SIGNALING | 0.03 | 0.39 |
| WP_PREGNANE_X_RECEPTOR_PATHWAY | 0.03 | 0.42 |
| WP_ALPHA_6_BETA_4_SIGNALING_PATHWAY | 0.04 | 0.43 |
| WP_IL17_SIGNALING_PATHWAY | 0.04 | 0.40 |
| WP_TGFBETA_SIGNALING_IN_THYROID_CELLS_FOR_EPITHELIALMESENCHYMAL_TRANSITION | 0.04 | 0.39 |
| WP_APOPTOSIS | 0.04 | 0.38 |
| WP_TAMOXIFEN_METABOLISM | 0.04 | 0.41 |
| WP_NOVEL_INTRACELLULAR_COMPONENTS_OF_RIGILIKE_RECEPTOR_PATHWAY | 0.04 | 0.39 |
| WP_THYROXINE_THYROID_HORMONE_PRODUCTION | 0.04 | 0.42 |
| WP_COMMON_PATHWAYS_UNDERLYING_DRUG_ADDICTION | 0.05 | 0.41 |
| WP_15Q133_COPY_NUMBER_VARIATION_SYNDROME | 0.05 | 0.43 |
| WP_MIRNA_REGULATION_OF_P53_PATHWAY_IN_PROSTATE_CANCER | 0.05 | 0.43 |

**Abbreviations:** GSEA: Gene set enrichment analysis; NES: Standardized enrichment score; P-value: Probability.
