# Supplementary material for: Gefitinib metabolism-related lncRNAs for the prediction of prognosis, tumor microenvironment and drug sensitivity in lung adenocarcinoma
Source: Sci Rep. 2024 May 6;14:10348. doi: 10.1038/s41598-024-61175-3 (PMC11074108; doi:10.1038/s41598-024-61175-3)
Supplement: Supplementary file 23 — Supplementary Table S9. [file 41598_2024_61175_MOESM23_ESM.docx]

**Table S9** Results of GSEA in the low-risk group.

| **Category** | **NES** | **P-value** |
| --- | --- | --- |
| **GO** |  |  |
| GOCC_TERMINAL_BOUTON | -1.77425 | 0.00641 |
| GOCC_MHC_CLASS_II_PROTEIN_COMPLEX | -1.68943 | 0.015968 |
| GOMF_MRNA_REGULATORY_ELEMENT_BINDING_TRANSLATION_REPRESSOR_ACTIVITY | -1.70012 | 0.018557 |
| GOCC_IMMUNOGLOBULIN_COMPLEX_CIRCULATING | -1.86756 | 0.027197 |
| GOBP_NEGATIVE_REGULATION_OF_B_CELL_MEDIATED_IMMUNITY | -1.61161 | 0.028866 |
| GOBP_IMMUNOGLOBULIN_PRODUCTION | -1.6965 | 0.031381 |
| GOCC_IMMUNOGLOBULIN_COMPLEX | -1.88297 | 0.031915 |
| GOCC_AXON_INITIAL_SEGMENT | -1.57595 | 0.03268 |
| GOBP_NEUROMUSCULAR_SYNAPTIC_TRANSMISSION | -1.63406 | 0.034483 |
| GOBP_DOPAMINE_TRANSPORT | -1.63581 | 0.03617 |
| GOBP_HUMORAL_IMMUNE_RESPONSE_MEDIATED_BY_CIRCULATING_IMMUNOGLOBULIN | -1.79596 | 0.037344 |
| GOBP_B_CELL_RECEPTOR_SIGNALING_PATHWAY | -1.74091 | 0.038544 |
| GOBP_COMPLEMENT_ACTIVATION | -1.77141 | 0.038627 |
| GOBP_REGULATION_OF_B_CELL_ACTIVATION | -1.64983 | 0.041667 |
| GOBP_MONOAMINE_TRANSPORT | -1.51628 | 0.042373 |
| GOMF_IMMUNOGLOBULIN_RECEPTOR_BINDING | -1.78443 | 0.042463 |
| GOMF_IMMUNOGLOBULIN_BINDING | -1.62923 | 0.044266 |
| GOBP_PHAGOCYTOSIS_RECOGNITION | -1.80534 | 0.044681 |
| GOMF_ANTIGEN_BINDING | -1.73043 | 0.046316 |
| GOBP_DOPAMINE_SECRETION | -1.51606 | 0.049462 |
| **WP** |  |  |
| WP_SYNAPTIC_VESICLE_PATHWAY | -1.49353 | 0.070281 |
| WP_PHOTODYNAMIC_THERAPYINDUCED_UNFOLDED_PROTEIN_RESPONSE | -1.508 | 0.082353 |
| WP_NICOTINE_EFFECT_ON_DOPAMINERGIC_NEURONS | -1.35262 | 0.126068 |
| WP_LIPID_METABOLISM_IN_SENESCENT_CELLS | -1.31955 | 0.153101 |
| WP_GENES_TARGETED_BY_MIRNAS_IN_ADIPOCYTES | -1.3158 | 0.165067 |
| WP_CYTOPLASMIC_RIBOSOMAL_PROTEINS | -1.41593 | 0.211417 |
| WP_PURINERGIC_SIGNALING | -1.17719 | 0.260355 |
| WP_PATHOGENESIS_OF_SARSCOV2_MEDIATED_BY_NSP9NSP10_COMPLEX | -1.24238 | 0.270217 |
| WP_VITAMIN_DSENSITIVE_CALCIUM_SIGNALING_IN_DEPRESSION | -1.13592 | 0.280242 |
| WP_DOPAMINERGIC_NEUROGENESIS | -1.14528 | 0.288845 |
| WP_NOTCH1_REGULATION_OF_ENDOTHELIAL_CELL_CALCIFICATION | -1.17419 | 0.288931 |
| WP_FOXP3_IN_COVID19 | -1.16581 | 0.313492 |
| WP_PEPTIDE_GPCRS | -1.11787 | 0.315369 |
| WP_LEUCINE_ISOLEUCINE_AND_VALINE_METABOLISM | -1.16729 | 0.320565 |
| WP_MICROGLIA_PATHOGEN_PHAGOCYTOSIS_PATHWAY | -1.16765 | 0.322449 |
| WP_HIPPOCAMPAL_SYNAPTOGENESIS_AND_NEUROGENESIS | -1.0943 | 0.361829 |
| WP_MAJOR_RECEPTORS_TARGETED_BY_EPINEPHRINE_AND_NOREPINEPHRINE | -1.02591 | 0.411881 |
| WP_NEUROGENESIS_REGULATION_IN_THE_OLFACTORY_EPITHELIUM | -1.01634 | 0.434615 |
| WP_EXTRAFOLLICULAR_B_CELL_ACTIVATION_BY_SARSCOV2 | -1.01427 | 0.442 |
| WP_ALLOGRAFT_REJECTION | -0.97356 | 0.465619 |
| WP_UREA_CYCLE_AND_METABOLISM_OF_AMINO_GROUPS | -0.95872 | 0.479208 |
| WP_CHOLESTEROL_METABOLISM_WITH_BLOCH_AND_KANDUTSCHRUSSELL_PATHWAYS | -0.94571 | 0.5 |
| WP_LIPID_METABOLISM_PATHWAY | -0.94078 | 0.518304 |
| WP_BDNFTRKB_SIGNALING | -0.9388 | 0.52552 |
| WP_METHIONINE_DE_NOVO_AND_SALVAGE_PATHWAY | -0.93273 | 0.541414 |
| WP_DEGRADATION_PATHWAY_OF_SPHINGOLIPIDS_INCLUDING_DISEASES | -0.90998 | 0.552529 |
| WP_MITOCHONDRIAL_COMPLEX_III_ASSEMBLY | -0.88247 | 0.561441 |
| WP_SPHINGOLIPID_METABOLISM_INTEGRATED_PATHWAY | -0.88467 | 0.565637 |
| WP_PRION_DISEASE_PATHWAY | -0.87286 | 0.574757 |
| WP_SUDDEN_INFANT_DEATH_SYNDROME_SIDS_SUSCEPTIBILITY_PATHWAYS | -0.91994 | 0.581532 |
| WP_IMATINIB_AND_CHRONIC_MYELOID_LEUKEMIA | -0.89604 | 0.584139 |
| WP_THERMOGENESIS | -0.85203 | 0.626984 |
| WP_RENINANGIOTENSINALDOSTERONE_SYSTEM_RAAS | -0.89187 | 0.627413 |
| WP_TYROBP_CAUSAL_NETWORK_IN_MICROGLIA | -0.7736 | 0.63189 |
| WP_PROXIMAL_TUBULE_TRANSPORT | -0.88651 | 0.632495 |
| WP_TUMOR_SUPPRESSOR_ACTIVITY_OF_SMARCB1 | -0.82407 | 0.637236 |
| WP_FATTY_ACID_BETAOXIDATION | -0.83712 | 0.638945 |
| WP_TYPE_II_DIABETES_MELLITUS | -0.84682 | 0.660853 |
| WP_RETINOBLASTOMA_GENE_IN_CANCER | -0.73834 | 0.673428 |
| WP_FATTY_ACID_BIOSYNTHESIS | -0.78961 | 0.682635 |
| WP_SEROTONIN_AND_ANXIETY | -0.84125 | 0.699605 |
| WP_SPHINGOLIPID_METABOLISM_IN_SENESCENCE | -0.78049 | 0.709369 |
| WP_ENDOPLASMIC_RETICULUM_STRESS_RESPONSE_IN_CORONAVIRUS_INFECTION | -0.76759 | 0.711321 |
| WP_CHOLESTEROL_SYNTHESIS_DISORDERS | -0.71 | 0.724138 |
| WP_2586_ARYL_HYDROCARBON_RECEPTOR_PATHWAY | -0.75853 | 0.730845 |
| WP_ELECTRON_TRANSPORT_CHAIN_OXPHOS_SYSTEM_IN_MITOCHONDRIA | -0.66856 | 0.73431 |
| WP_SPHINGOLIPID_PATHWAY | -0.76338 | 0.735521 |
| WP_IL2_SIGNALING_PATHWAY | -0.72381 | 0.750996 |
| WP_DRAVET_SYNDROME | -0.73993 | 0.754864 |
| WP_MITOCHONDRIAL_COMPLEX_I_ASSEMBLY_MODEL_OXPHOS_SYSTEM | -0.67474 | 0.758763 |
| WP_TRANSSULFURATION_ONECARBON_METABOLISM_AND_RELATED_PATHWAYS | -0.76757 | 0.770093 |
| WP_RETT_SYNDROME_CAUSING_GENES | -0.71378 | 0.797665 |
| WP_PHOSPHODIESTERASES_IN_NEURONAL_FUNCTION | -0.74677 | 0.802789 |
| WP_MAP3K1_ROLE_IN_PROMOTING_AND_BLOCKING_GONADAL_DETERMINATION | -0.71134 | 0.804 |
| WP_LTF_DANGER_SIGNAL_RESPONSE_PATHWAY | -0.64879 | 0.81336 |
| WP_PHYSIOLOGICAL_AND_PATHOLOGICAL_HYPERTROPHY_OF_THE_HEART | -0.69405 | 0.814672 |
| WP_SPHINGOLIPID_METABOLISM_OVERVIEW | -0.70001 | 0.817308 |
| WP_EUKARYOTIC_TRANSCRIPTION_INITIATION | -0.63437 | 0.818548 |
| WP_CLASSICAL_PATHWAY_OF_STEROIDOGENESIS_WITH_GLUCOCORTICOID_AND_MINERALOCORTICOID_METABOLISM | -0.78918 | 0.824458 |
| WP_EICOSANOID_SYNTHESIS | -0.70511 | 0.825147 |
| WP_DNA_MISMATCH_REPAIR | -0.57626 | 0.833333 |
| WP_ETHANOL_EFFECTS_ON_HISTONE_MODIFICATIONS | -0.70218 | 0.83953 |
| WP_G1_TO_S_CELL_CYCLE_CONTROL | -0.5458 | 0.88843 |
| WP_GASTRIC_CANCER_NETWORK_2 | -0.53856 | 0.89759 |
| WP_PI3KAKTMTOR_SIGNALING_PATHWAY_AND_THERAPEUTIC_OPPORTUNITIES | -0.61462 | 0.897683 |
| WP_OXIDATIVE_PHOSPHORYLATION | -0.48979 | 0.910448 |
| WP_UNFOLDED_PROTEIN_RESPONSE | -0.58615 | 0.927481 |
| WP_COMPLEMENT_ACTIVATION | -0.52749 | 0.950199 |
| WP_STATIN_INHIBITION_OF_CHOLESTEROL_PRODUCTION | -0.5301 | 0.959596 |
| WP_COVID19_ADVERSE_OUTCOME_PATHWAY | -0.51787 | 0.962451 |
| WP_FARNESOID_X_RECEPTOR_PATHWAY | -0.54504 | 0.981964 |

**Abbreviations:** GSEA: Gene set enrichment analysis; NES: Standardized enrichment score; P-value: Probability.
