# Supplementary material for: Gefitinib metabolism-related lncRNAs for the prediction of prognosis, tumor microenvironment and drug sensitivity in lung adenocarcinoma
Source: Sci Rep. 2024 May 6;14:10348. doi: 10.1038/s41598-024-61175-3 (PMC11074108; doi:10.1038/s41598-024-61175-3)
Supplement: Supplementary file 24 — Supplementary Legends. [file 41598_2024_61175_MOESM24_ESM.docx]

**Supplementary Data**

**Figure S1** Screening of lncRNA related to gefitinib metabolism. (A) Sankey plot of co-expression of gefitinib-related genes and lncRNAs; (B) Coefficient distribution plot of Lasso regression analysis; (C) Cross Validation Curve for Lasso Regression Analysis.

**Figure S2** Risk score plot of the model. (A) Overview of models in the training group; (B) Overview of models in the verification group; (C) Overview of models in the entire group.

**Figure S3** Validation of model effect. (A) Nomogram constructed by clinicopathological features; (B) 1-year calibration curve of nomogram; (C) 3-year calibration curve of nomogram; (D) 5-year calibration of nomogram Curve; (E)Comparison of risk score model and clinicopathological feature model.

**Figure S4** Correlation of clinical features. (A) Relationship between age and risk score; (B) Relationship between gender and risk score; (C) Relationship between stage and risk score; (D) Relationship between T stage and risk score; (E) Relationship between N stage and risk score.

**Figure S5** KM curves validated by clinical subgroup data. (A) <65; (B) >65; (C) Male; (D) Female; (E) White; (F) Asian; (G) Black or African American; (H) Stage I; (I) Stage II ;(J) Stage III; (K) Stage IV; (L) Stage T1; (M) Stage T2; (N) Stage T3; (O) Stage T4; (P) Stage N0; (Q) Stage N1; (R) Stage N2; (S) Stage M0; (T) Stage M1.

**Figure S6** Gene PCA analysis. (A) PCA analysis of all genes in LUAD patients; (B) PCA analysis of genes related to gefitinib metabolism; (C) PCA analysis of gefitinib metabolism-related lncRNAs; (D) PCA analysis of lncRNAs related to gefitinib metabolism after grouping.

**Figure S7** GSEA enrichment analysis. (A) GSEA analysis of GO database; (B) GSEA analysis of Reactome database; (C) GSEA analysis of Biocarta databases; (D) GSEA analysis of PID database; (E) GSEA analysis of WIKI Pathway database.

**Figure S8** Tumor immune microenvironment analysis. (A)Seven kinds of immune algorithm immune cell infiltration heat map; (B) CIBERSOFT algorithm immune cell infiltration differential analysis; (C) Immune-related pathway enrichment analysis in the training group; (D) Immune-related pathway enrichment analysis in the verification group; (E) Immune-related pathway enrichment analysis in the entire group.

**Figure S9** Immune Evasion Analysis Between High and Low Risk Groups. (A) TIDE score in the training group; (B) TIDE score in the validation group; (C) TIDE score in the entire group; (D) MDSC score in the training group; (E) MDSC score in the validation group; (F) MDSC score in the entire group; (G) Exclusion score in the training group; (H) Exclusion score in the validation group; (I) Exclusion score in the entire group; (J) CAF score in the training group; (K) CAF score in the validation group; (L) CAF score in the entire group; (M) IFNG score in the training group; (N) IFNG score in the validation group; (O) IFNG score in the entire group.

**Figure S10** Immune Evasion Analysis Between High and Low Risk Groups. (A) TAMM2 score in the training group; (B) TAMM2 score in the validation group; (C) TAMM2 score in the entire group; (D) MSI score in the training group; (E) MSI score in the validation group; (F) MSI score in the entire group; (G) Merck18 score in the training group; (H) Merck18 score in the validation group; (I) Merck18 score in the entire group; (J) Dysfunction score in the training group; (K) Dysfunction score in the validation group; (L) Dysfunction score in the entire group; (M) CD8 score in the training group; (N) CD8 score in the validation group; (O) CD8 score in the entire group; (P) CD274 score in the training group; (Q) CD274 score in the validation group; (R) CD274 score in the entire group.

**Figure S11** TMB analysis. (A) Differences in TMB between high and low risk groups in the training group; (B) Differences in TMB between high and low risk groups in the validation group; (C) Differences in TMB between high and low risk groups in the entire group; (D) The KM curve of the high and low TMB group in the training group; (E) The KM curve of the high and low TMB group in the validation group; (F) The KM curve of the high and low TMB group in the entire group; (G) survival curves for the interaction of risk score with H-TMB and L-TMB in the training group; (H) survival curves for the interaction of risk score with H-TMB and L-TMB in the validation group; (I) survival curves for the interaction of risk score with H-TMB and L-TMB in the entire group.

**Figure S12** Chemotherapy Drug Prediction. (A) Differences of IC50 of SCH772948_1564 in high and low risk groups; (B) Differences of IC50 of ERK_6604_1714 in high and low risk groups; (C) Differences of IC50 of Selumetinib_1736 in high and low risk groups; (E) Correlation plot of risk score and IC50 of SCH772948_1564; (F) Correlation plot of risk score and IC50 of ERK_6604_1714; (G) Correlation plot of risk score and IC50 of SCH772948_1564.

**Figure S13** IMvigor210 Bladder Cancer Model Validation. (A) KM curve of target lncRNAs high and low expression group; (B) ROC curve of the prediction model constructed by the target lncRNAs; (C) Target gene expression difference analysis of IMvigor210 bladder cancer model.

**Figure S14** Stem Cell Index Clinical Correlation. (A) KM curves of mRNAsi high expression and low expression subgroups; (B) differences in mRNAsi between tumor samples and normal samples; (C) Correlation of mRNAsi with gender; (D) Correlation of mRNAsi with Stage T; (E) Correlation of mRNAsi with Stage M; (F) Correlation of mRNAsi with grade.

**Table S1** Primer sequences for nine GMLncs.

**Table S2** The specific information of 128 GMlncs screened after univariate cox analysis on Train cohort.

**Table S3** Genetic information after multivariate cox analysis.

**Table S4** Related phenotypes and potential target proteins of 9 GMLncRNAs.

**Table S5** Results of independent prognostic analyses.

**Table S6** All pathways of GO enrichment analysis.

**Table S7** All pathways of KEGG enrichment analysis.

**Table S8** Results of GSEA in the high-risk group.

**Table S9** Results of GSEA in the low-risk group.
